# Revisiting the role of behavior-mediated structuring in the survival of populations in hostile environments

**DOI:** 10.1038/s42003-023-05731-z

**Published:** 2024-01-12

**Authors:** Simran Sandhu, Victor Mikheev, Anna Pasternak, Jouni Taskinen, Andrew Morozov

**Affiliations:** 1 School of Computing and Mathematical Sciences, University of Leicester, LE1 7RH Leicester, UK; 2 Institute of Ecology and Evolution, Russian Academy of Sciences, Moscow, Russia; 3 Institute of Oceanology, Russian Academy of Sciences, Moscow, Russia; 4 Department of Biological and Environmental Science, University of Jyväskylä, Jyväskylä, Finland

**Keywords:** Computational models, Behavioural ecology, Evolutionary theory, Agroecology, Ecological modelling

## Abstract

Increasing the population density of target species is a major goal of ecosystem and agricultural management. This task is especially challenging in hazardous environments with a high abundance of natural enemies such as parasites and predators. Safe locations with lower mortality have been long considered a beneficial factor in enhancing population survival, being a promising tool in commercial fish farming and restoration of threatened species. Here we challenge this opinion and revisit the role of behavior structuring in a hostile environment in shaping the population density. We build a mathematical model, where individuals are structured according to their defensive tactics against natural enemies. The model predicts that although each safe zone enhances the survival of an individual, for an insufficient number of such zones, the entire population experiences a greater overall mortality. This is a result of the interplay of emergent dynamical behavioral structuring and strong intraspecific competition for safe zones. Non-plastic structuring in individuals’ boldness reduces the mentioned negative effects. We demonstrate emergence of non-plastic behavioral structuring: the evolutionary branching of a monomorphic population into a dimorphic one with bold/shy strains. We apply our modelling approach to explore fish farming of salmonids in an environment infected by trematode parasites.

## Abstract (web summary)

A theoretical model, based on empirical data, predicts that population structuring according to defensive tactics used against natural enemies enhances the population mortality, whereas nonplastic (permanent) structuring reduces this negative effect.

## Introduction

Animal populations often inhabit hazardous environments characterized by pronounced mortality due to a high presence of natural enemies, parasites and predators. A common goal in agricultural and ecosystem management is to enhance the population density of certain target species, which can be challenging in hazardous environments^1^. Among other factors, the availability of local safe zones such as refuges or shelters, with lower mortality and/or higher reproduction rates, seems to be beneficial for population proliferation^2^. For example, the creation of artificial shelters in aquatic farms, or, generally, promotion of heterogeneity in natural aquatic systems is believed to be an efficient practical tool to reduce the stress of fish and protect them from parasites and predators^3,4^. Overall, one can consider safe zones as a sort of generalized resource; therefore providing an extra vital resource to a population is expected to produce a positive impact as compared to the scenario without this resource (all other conditions being equal). Here we question this conventional ecological wisdom, and we argue that the presence of safe zones, being largely beneficial at the scale of an individual, can be harmful at the scale of the entire population. Using a theoretical model with realistic parameters, describing salmonids-parasites interactions, we uncover a counter-intuitive scenario, where the behavioral structuring in a heterogeneous environment has a negative impact on the overall survival and proliferation of the population.

Animal populations are often behaviorally structured, in terms of the strategies individuals use to compete with each other and evade their natural enemies^5^. Such structuring can be dynamic, when individuals can use different strategies under different circumstances. For example, boldness in the pumpkinseed sunfish (*Lepomis gibbosus*) disappears when fish are in social and ecological isolation^6^. Within a single population, individuals can split into groups that implement distinct behavioral strategies. For example, for many fish species, a part of the individuals can use shoaling (grouping)^7^, whereas the other part of the population can show strong territoriality (sheltering)^8^. On the other hand, structured behavior can also be permanent over the lifetime of an organism: individuals can highly vary in their rates of exploration of the environment, risk-taking willingness, reactivity, aggressiveness, or overall activity^9–12^. Although behavioral structuring (dynamic or/and permanent) is now well-recognized in the ecological literature^13,14^, it is still unclear how this could mediate intraspecific competition for vital resources when competition comes at a high cost and how this could affect the population density. It is also unknown how dynamic behavioral structuring triggered by external stimuli, such as natural enemies and/or environmental heterogeneity (e.g. the presence of safe zones), interacts with permanent behavioral structuring to shape the size of the entire population.

To assess the role of behavioral structuring in population proliferation, we build a generic mathematical model involving several time scales: behavioral, demographic and evolutionary. We are interested in whether adding safe zones (shelters) for individuals in a hostile environment formed by natural enemies (parasites and/or predators) is always beneficial. Using the theoretical model with realistic parameters related to fish farming, we show that the dynamical behavioral structuring caused by spatial heterogeneity mostly lowers the population density, in the case where the available safe zones are insufficient. The permanent structuring of the population in terms of boldness, aggressiveness, or reactivity has the opposite effect. The mentioned positive effect seems to be generic, observed both in clonal and non-clonal scenarios of the inheritance of behavioral traits. The model predicts that permanent behavioral structuring can arise from an initially uniform (monomorphic) population as a result of disruptive evolution via evolutionary branching.

As a practical application of our theory, we consider the interaction between salmonid fish and trematode parasites in the presence of artificial shelters in fish farms. The dynamical behavioral structuring in salmonids includes shoaling (grouping) and sheltering (territoriality)^7,8^: both tactics, besides the direct effect of antipredator defense, reduce stress and the ventilation rate in fish^15–17^. Salmonids prefer shelters to shoals making shelters very contestable^8,18^. Fighting for shelter is costly due to the threat of predation and acquisition of extra parasites resulting in higher mortality^8,19,20^. We apply our theoretical model to the system comprising rainbow trout *Oncorhynchus mykiss*, widely grown in fish farms, and the common trematode parasite, eye-fluke *Diplostomum pseudospathaceum*. For this system, we also experimentally explore the missed so far connection between permanent behavioral structuring of rainbow individuals (reactivity) with vulnerability to infection by *D. pseudospathaceum* as well as the consequences of using various antipredator strategies for parasite acquisition.

We conclude that installing an insufficient number of shelters in aquaculture and restoration programs would be counter-productive in terms of having a higher parasite acquisition, and a decrease in the overall population size. The proposed theoretical framework can be also applied to better understand the role of behavioral structuring on the population dynamics of some coral fish^21^ as well as freshwater fish^22^.

## Results

We developed a theoretical model (see Methods) exploring the role of intraspecific competition within a behaviorally structured population that resides in a hostile environment, containing parasites. The model uses parameters describing interaction between salmonids and their trematode parasites (see Methods). The meaning of the variables and model parameters is summarized in Table 1. Using the model, we assess the population density under different scenarios.

**Table 1:** Definitions of model variables, functions, parameters, units as well as their ranges and default values for the rainbow trout—parasites system.

Component	Meaning	Formulation, parameter range, unit, and default value (DEF)
*B*_*i*_	Boldness of individuals of strain *i*	0≤*B*_*i*_≤1, dimensionless
*T*_*i*_	Individuals of strain *i* currently showing behavior T (sheltering)	*individuals*
*S*_*i*_	Individuals of strain *i* currently showing behavior S (shoaling)	*individuals*
*F*_*i*_	Total number of individuals of strain *i*	*individuals*
*K*	Carrying capacity of the population	10^3^ < *K* < 2.5 ⋅ 10^4^ *individuals*, DEF: *K* = 10^4^ *individuals*
*N*	Total number of shelters	0≤*N*≤*K*, *shelters*
*ω*(*B*_*i*_, *B*_*j*_)	Probability that an individual in the shoal of strain *i* attempts to invade a shelter occupied by an individual of strain *j*	$\omega ({B}_{i},{B}_{j})=\frac{{e}^{-{\delta }_{\omega }({B}_{j}-{B}_{i})}}{1+{e}^{-{\delta }_{\omega }({B}_{j}-{B}_{i})}}$
*δ*_*ω*_	Characteristic coefficient in the probability function *ω*(*B*_*i*_, *B*_*j*_)	*δ*_*ω*_ > 20, DEF: *δ*_*ω*_ = 30, dimensionless
*I*(*B*_*i*_, *B*_*j*_, *T*_*j*_)	Rate at which shoal individuals of strain *i* attempt to invade shelters of territorial individuals of strain *j*	*I*(*B*_*i*_, *T*_*j*_) = *I*_0_*ν*(*B*_*i*_)*ω*(*B*_*i*_, *B*_*j*_)*T*_*j*_
*I*_0_	Maximal search rate for shelters	*I*_0_ ∈ [4, 28]*year*^−1^, DEF:*I*_0_ = 15 year^−1^
*ν*(*B*_*i*_)	Dependence of the search rate for shelters on boldness *i*	$\nu (B)=\frac{{B}_{i}^{\mu }}{{B}_{i}^{\mu }+{B}_{\nu }^{\mu }}$
*B*_*ν*_, *μ*	Parameters characterizing *ν*(*B*_*i*_)	2 < *μ* < 8 DEF *μ* = 5; 0.2 < *B*_*ν*_ < 0.7 DEF: *B*_*ν*_ = 0.5, dimensionless
*R*(*B*_*i*_, **F**^a^)	Redistribution of offspring of strain *i*	$R({B}_{i},{{{{{{{\bf{F}}}}}}}})=\sum {A}_{j}{F}_{j}\exp \left(-\frac{{({B}_{j}-{B}_{i})}^{2}}{{D}_{w}}\right)$, where *A*_*j*_ are normalizing constants
*D*_*w*_	Width of the kernel *R*(*B*_*i*_, **F**)	1 ⋅ 10^−6^≤*D*_*w*_≤1 ⋅ 10^6^, dimensionless
$b(\mathop{\sum }\nolimits_{j = 1}^{n}{F}_{j})$	Per capita growth rate of all strains	$b(\mathop{\sum }\nolimits_{j = 1}^{n}{F}_{j})={b}_{0}\left(1-\frac{\mathop{\sum }\nolimits_{j = 1}^{n}{F}_{j}}{K}\right)$
*b*_0_	Maximal per capita birth rate	1.6 < *b*_0_ < 5*year*^−1^, DEF: *b*_0_ = 2 year^−1^
*D*	Reduction in the cost of fighting when defending a shelter	0.2 < *D* < 1 DEF: *D* = 0.4, dimensionless
*m*	Natural background mortality rate	0.07 < *m*_0_ < 0.25 year^−1^ DEF: *m*_0_ = 0.13 yea*r*^−1^
Δ*m*_*S*_(*B*_*i*_)	Parasite/predator mortality for shoaling individuals of strain *i*	Δ*m*_*S*_(*B*_*i*_) = Δ_*S*_(1 − *ϵB*_*i*_), 0.1 < Δ_*S*_ < 2.5 year^−1^, DEF: Δ_*S*_ = 0.9 year^−1^
Δ*m*_*T*_(*B*_*i*_)	Parasite/predator mortality for sheltering individuals of strain *i*	Δ*m*_*T*_(*B*_*i*_) = Δ_*T*_(1 − *ϵB*_*i*_), 0.05 < Δ_*T*_ < 1.6 year^−1^, DEF: Δ_*T*_ = 0.45 year^−1^
*m*_*p*_(*B*_*i*_)	Extra mortality due to competitions for shelters between resident and invading shoal individuals	*m*_*p*_(*B*_*i*_) = *ν*_*m*_(1 − *ϵB*_*i*_)
*ν*_*m*_	Maximal mortality rate due to contests for shelters (per shelter)	0.0005 < *ν*_*m*_ < 0.5year^−1^, DEF: *ν*_*m*_ = 0.02 year^−1^
*ϵ*	Parameter, incorporating dependence on mortality rate on boldness	0.075 < *ϵ* < 1, DEF: *ϵ* = 0.1, dimensionless

The considered spatial area is 1*ha*. The estimates of model parameters are discussed in the subsection ‘Estimation of model parameters’ of the ‘Methods’ section. Italic and bold highlighting styles correspond to scalar and vector (multi-variable) quantities, respectively.

^a^Bold variable **F** is a vector with components *F*_*i*_.

### Monomorphic population

**Fig. 1:**
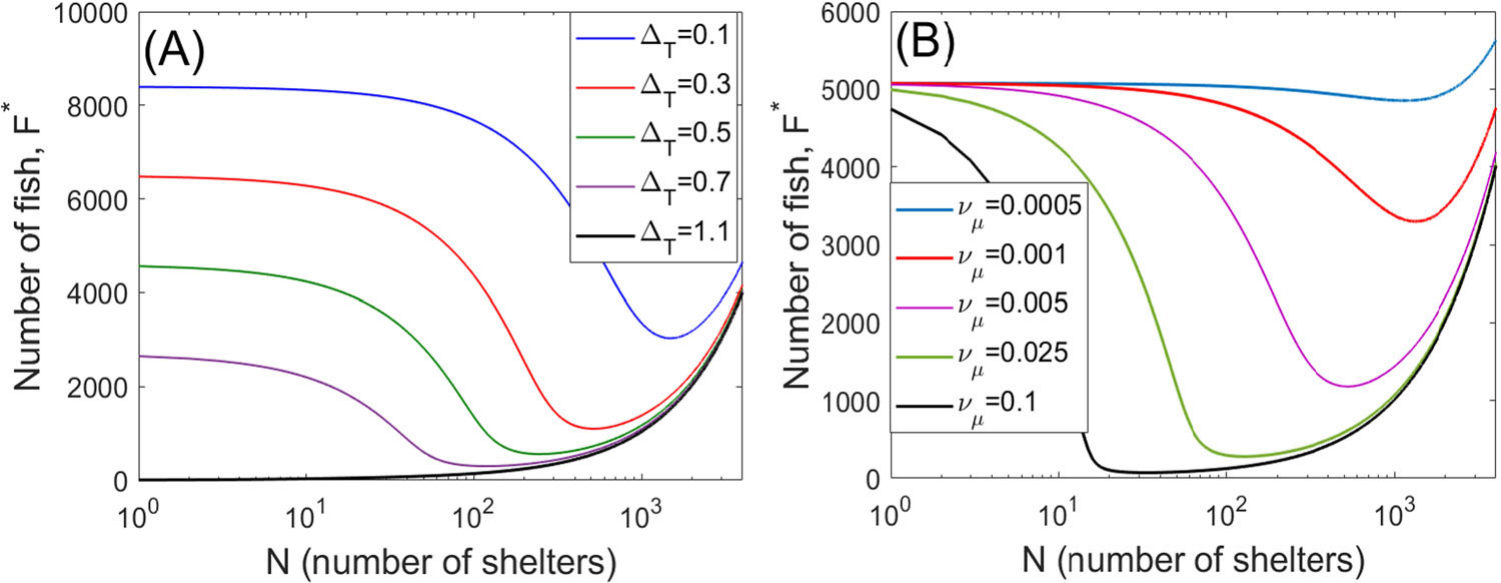
Dependence of the equilibrium population density F^*^ (measured in individuals) on the number of shelters *N* in a behaviorally monomorphic population. Panel (**A**) shows the graphs F^*^(*N*) obtained for different mortality pressures due to variation of the abundance of natural enemies, described by the parameter Δ_*T*_ (measured in year^−1^). Here we assume that the parameters Δ_*T*_ and *ν*_*m*_, accounting for the mortality due to natural enemies, vary proportionally with Δ_*T*_, since they incorporate the abundance of the natural enemies. The coefficients of proportionality are computed using the default values of parameters (see Table 1). Panel (**B**) shows the graphs F^*^(*N*) constructed for different values of the parasite acquisition rate *ν*_*μ*_ (measured in year^−1^) due to fighting for shelters. The considered spatial area is 1 ha. The other parameters are the same as in Table 1.

First, we explore the scenario, where all individuals are identical in terms of their permanent behavioral structuring, however, the population is heterogeneous dynamically in terms of defence behavior. We are interested in the dependence of the equilibrium population density F^*^ on the number of available safe zones (shelters) *N* for different levels of threat from natural enemies (parasites and predators) in the system. Fig. 1A displays the dependence of the population density F^*^ on shelter numbers *N* for various levels of abundance of natural enemies. Since the abundance of the natural enemies affects three related parameters Δ_*T*_, Δ_*S*_ and *ν*_*m*_ (mortality rates), we consider that variation of one parameter (e.g. Δ_*T*_) results in a proportional change of the other two. The figure shows that for larger levels of top-down regulation leading to higher mortality (Δ_*T*_ = 1.1 year^−1^) individuals cannot survive in shoals and the population size approximately equals the number of shelters, F^*^ ≈ *N*. In this case, installing artificial shelters in the environment would therefore be highly beneficial. For lower levels of natural enemies, we observe an initial decrease in F^*^ when installing few shelters. For a further increase in *N*, after passing the minimal density, the population density starts increasing and, eventually, for large *N* it becomes larger than its value in the absence of shelters.

In Fig. 1B, the amount of natural enemies in the system is fixed, with the cost of contesting shelters *ν*_*μ*_ being varied. Note that the parameter *ν*_*μ*_ combines the effects of the cost of fighting and the frequency of finding a shelter (see Methods). At low *ν*_*μ*_, adding shelters in the environment only results in a small drop in F^*^. Higher values of *ν*_*μ*_ (high costs of a contest or/and fast rates of finding a shelter) result in a pronounced drop in the population density after adding shelters. Our simulations of the model for other values of parameters do not qualitatively change the patterns shown in Fig. 1 (see Supplementary Note 8, Figs. S6–S12). Overall, the model suggests that only the creation of a sufficiently high number of shelters in the environment would increase the population density compared to the homogeneous habitat (*N* = 0). The detrimental impact of introducing an insufficient number of shelters is related to the conflict between individuals contesting shelters: fighting for shelters results in higher mortality imposed by parasites and predators. However, increasing the number of shelters at some point can compensate the negative effect of contests: the losses due to fighting for shelters become smaller than the benefits of staying in shelters.

### Polymorphic population

**Fig. 2:**
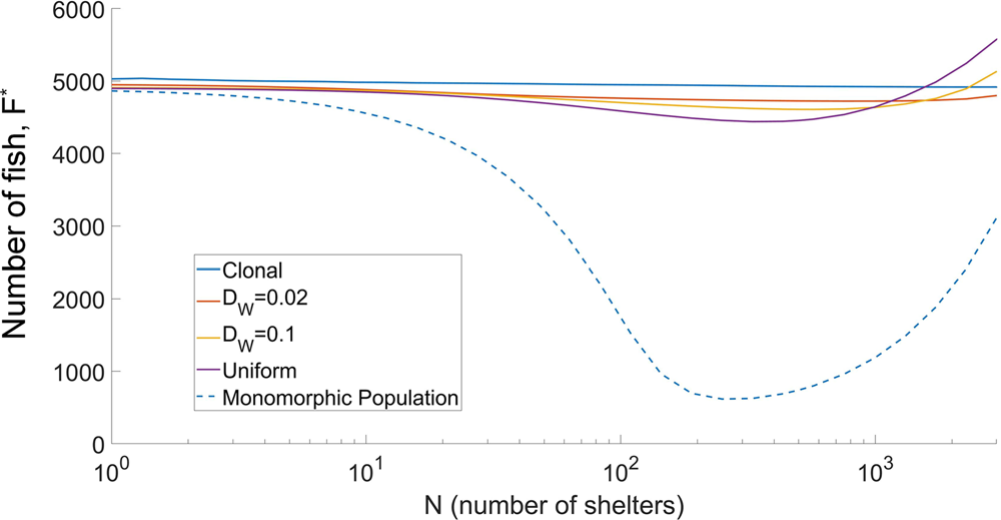
Dependence of the total population density ${F}^{* }=\sum {F}_{i}^{* }$ (measured in individuals) on the number of shelters *N* in a population of 20 strains of different, uniformly spaced, boldness of equal initial density (with *B* = 0.5 for the monomorphic population). The curves are obtained for varying values of *D*_*w*_. Clonal reproduction is assumed when *D*_*w*_ ≤ 10^−6^ and uniform reproduction is assumed when *D*_*w*_ ≥ 10^6^. Equilibrium densities are modeled using Eq. (7) (the demographic model) along with the solutions to the fast system given by (4); the model parameters are as given in Table 1. The initial distribution of individuals across boldness cohorts is assumed to be uniform. The considered spatial area is 1*ha*.

Now we consider a population that combines a permanent behavior structuring, which we denote by the term ‘boldness’, with dynamical structuring in terms of defence strategy. The total population density at equilibrium ${F}^{* }=\sum {F}_{i}^{* }$ is plotted as a function of the number of shelters *N* (see Fig. 2). In the example, we consider *n* = 20 different strains of boldness for varying values of *D*_*w*_, describing the strength of heredity of the boldness trait. For comparison, we also show the curve corresponding to the monomorphic population with boldness *B* = 0.5. One can see that in a behaviorally polymorphic population, adding shelters does not cause as drastic a drop in the population density compared to the monomorphic scenario. For a clonal reproduction scenario, the introduction of shelters did not affect the total population density. With uniform genetic mixing, the effect of adding shelters becomes beneficial for some large numbers of shelters (>25% of the carrying capacity) since the population density is larger than in a fully homogeneous environment.

**Fig. 3:**
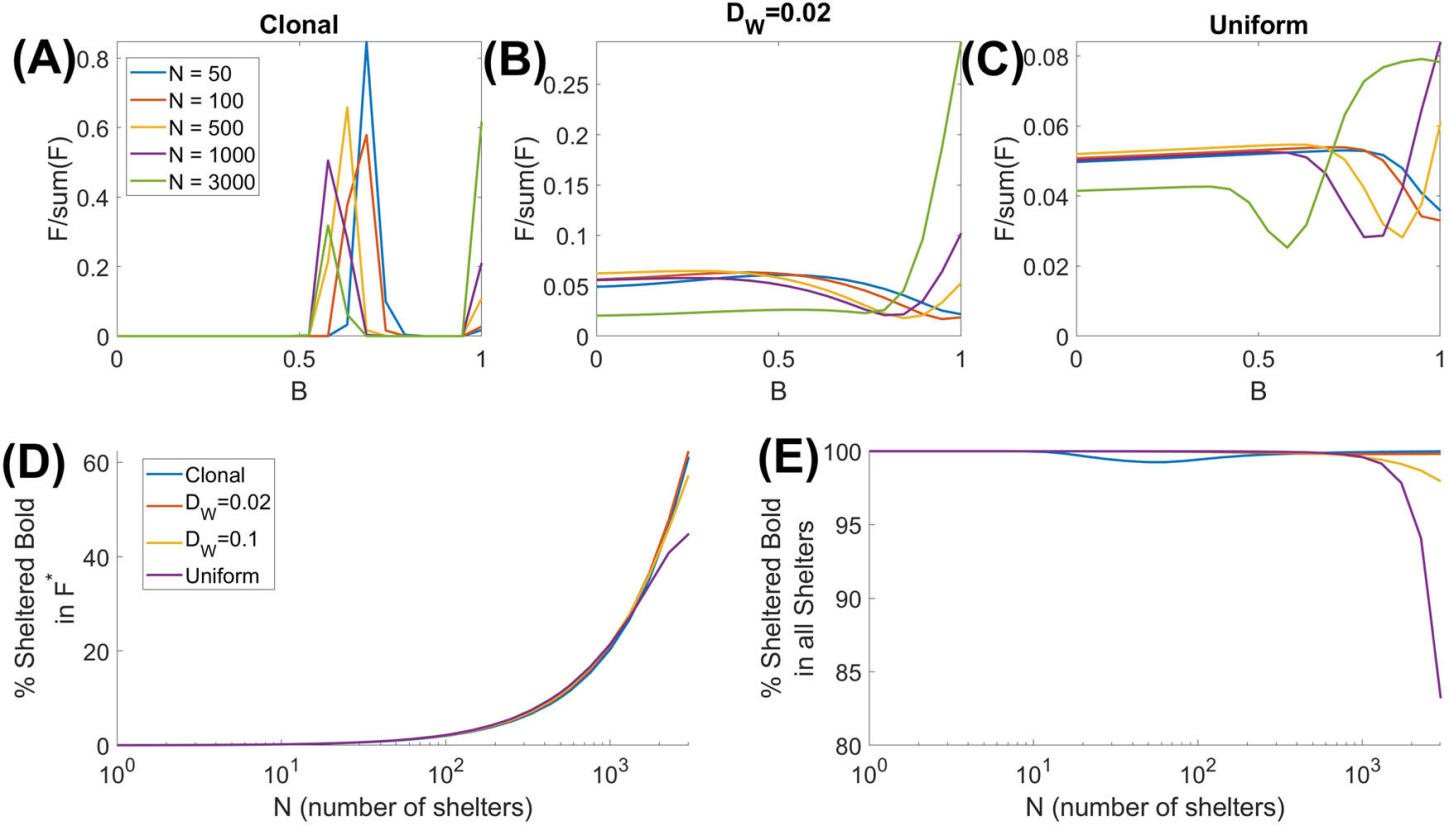
Distributions of boldness within the fish population for different mutation scenarios. Normalized distributions of boldness for various numbers of shelters *N* in the system in the cases where the redistribution of offspring is clonal (**A**), using a Gaussian mutation kernel with width *D*_*w*_ = 0.02 (**B**), and the uniform genetic mixing (**C**). **D** The proportion of the bold individuals (defined as *B* > 0.7) using territorial tactics (staying in shelters) calculated for the distributions in the upper panel. **E** Proportions of all sheltered individuals who are bold. All model parameters and settings are as described in Fig. 2.

The fact that a large drop in F^*^ is not observed in a polymorphic population—contrary to the monomorphic scenario—can be elucidated by plotting the normalized distributions of boldness for varying values of *N* and *D*_*w*_. The results are shown in Fig. 3. For clonal reproduction (Fig. 3A), the introduction of shelters in an initially homogeneous habitat triggers the emergence of two peaks in the distribution: one corresponding to intermediate values of boldness and the other one having the maximal boldness 1. Simulations show that individuals from the less-bold cohort mostly stay in the shoal, whereas shelters are occupied by the cohort with maximal boldness. The boldness of this shoal-based strain is the maximal boldness such that individuals can coexist in the shoal without competing with the bolder cohort for their shelter. This lack of competition between the shoal-based and the shelter-based individuals drastically drops the mortality as compared to the scenario with a monomorphic population, where all shoal-based individuals are constantly in competition for shelter. This is because shy individuals avoid contests with bold ones. Analytical computation using a simplified model confirms that for cohorts with distinctly different boldness, the total population density F^*^ is independent of *N* (Supplementary Note 6).

For a non-clonal inheritance of boldness, distributions of boldness become more even (see Fig. 3B). This is especially true for uniform genetic mixing (Fig. 3C). For a small number of shelters (*N* ≪ F^*^(0)), individuals with a high degree of boldness are rare. Adding more shelters generally results in the emergence of a gap between bold and shy cohorts, with a larger proportion of bold individuals. We also find that shelters are mostly occupied by bold individuals (see Fig. 3D, E). This reduces the negative effects of competition for shelters. An important feature of the distributions in Fig. 3B, C is the absence of a single dominant cohort for values of *N*, which corresponded to the drop in F^*^ in a monomorphic population. This largely reduces the mortality caused by contests for shelters in a polymorphic population. The eventual dominance by high-boldness cohorts for very large *N* (e.g. >25% of the carrying capacity) does not have pronounced negative impacts on the population density since in this case most of the population would use territorial tactics and stay in shelters. The lower peaks are dampened due to reproductive mutations, governed by the value of *D*_*w*_, if mutations occur often (e.g. for *D*_*w*_ = 0.02) then selection cannot maintain the two distinct strains of boldness.

When shelters become abundant in the environment, the total population density increases when genetic mixing is pronounced, especially for uniform mixing (Fig. 2). The reason for this is that for large *N*, the proportion of the population with bold behavior becomes dominant; those individuals -mostly occupying shelters- largely contribute to the reproduction of the shy cohorts, which in turn, mostly use the shoaling tactics. As a result, the per capita growth rate of shy cohorts increases, whereas their contribution to the production of bolder individuals decreases. This leads to an increase in the total population density. The above reasoning is confirmed by analytical computation in a simplified model with uniform mixing of two strains (Supplementary Note 6). The effect of increasing F^*^ for large *N*, however, is not observed in a structured population with clonal reproduction (Supplementary Note 6, Fig. S4). Extensive simulation of the model shows that variation of key model parameters does not qualitatively change the patterns obtained for the polymorphic population (see Supplementary Note 8, Figs. S13–S17).

### Impact of behavioral structuring on the success of natural enemies

**Fig. 4:**
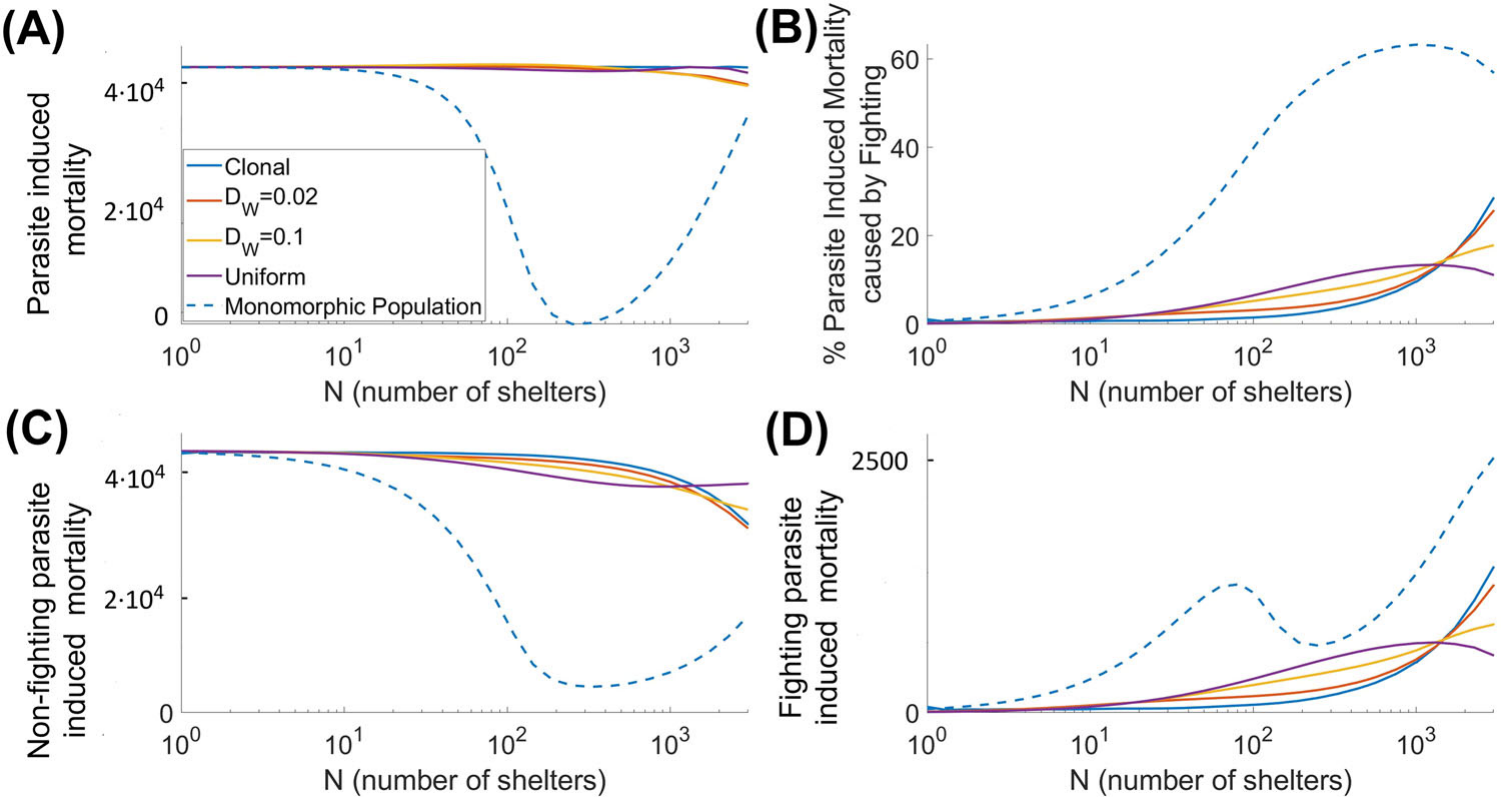
Dependence of parasite-induced mortality rates on the number of shelters *N*, with multiple boldness strains, for varying values of *D*_*w*_. The dashed curve shows the parasite-induced mortality in a single-strain population. **A** Total parasite-induced mortality (measured as individuals lost per year). **B** Relative contribution of fighting for shelters to the overall mortality; (**C**) and (**D**) present, respectively, the non-fighting and the fighting-related parasite-induced mortality rates (measured as individuals lost per year). All model parameters and settings are as described in Fig. 2.

We briefly explore the effects of behavioral structuring in the population on the success of its natural enemies. Here we focus on the proliferation of parasites in the environment. We do not model the dynamics of parasites explicitly but quantify the success of parasites using parasite-induced mortality as a proxy for parasite fitness. Figure 4 shows the dependence of the total parasite-induced mortality on the number of shelters for both monomorphic and polymorphic populations. For convenience, we separately plot the parasite-induced mortality due to fighting for shelters and the mortality, induced by parasites, when staying in the shoal or shelters.

Although the parasite-induced mortality due to contesting shelters is higher for a monomorphic population (Fig. 4B, D), the total parasite-induced mortality is larger for a polymorphic population (Fig. 4A). This occurs since the total number of potential hosts exploited by parasites is smaller for a monomorphic population due to a dramatic drop in the overall host population density. The proportionality between the fighting parasite-induced mortality and population density (or potential hosts) can explain the intermediate drop in the mortality for the monomorphic population (dashed blue curve in Fig. 4D). This signifies that having bold and shy individuals within a population should increase the transmission of parasites in the system, thus increasing their reproduction success. For a monomorphic population, a reduction in the number of shelters may reduce the total amount of parasites in the environment.

### Evolutionary branching as a possible scenario of behavioral structuring

**Fig. 5:**
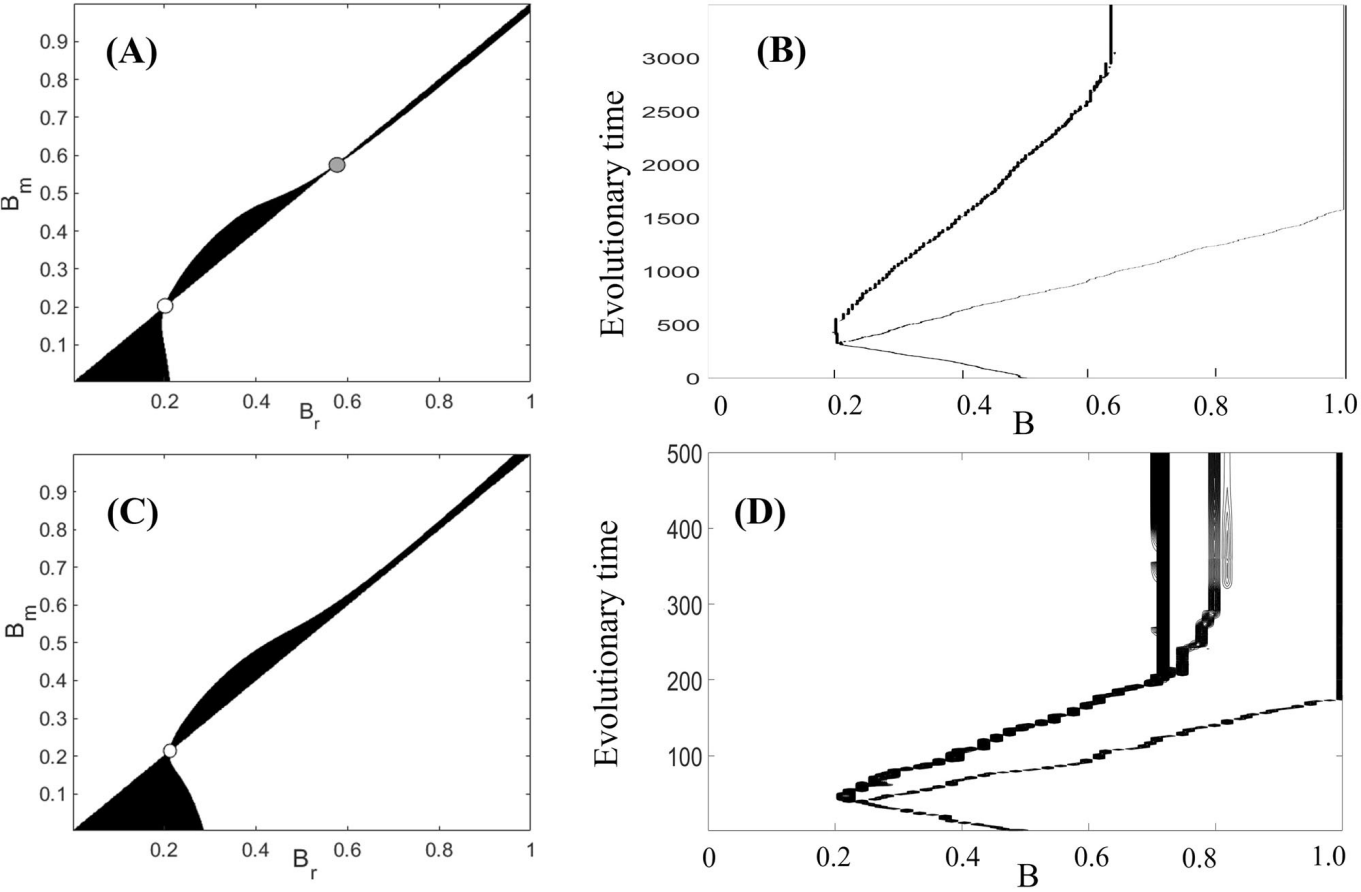
Evolution of boldness via the adaptive dynamics framework. **A**, **C** Pairwise Invasibility Plots (PIP) describing the invasion fitness of a rare mutant (*B*_*m*_) into the population of a resident strain (*B*_*r*_). The white regions represent a positive invasion fitness (*λ* > 0) and therefore a successful invasion, whereas the black regions represent a negative invasion fitness (*λ* < 0) and an unsuccessful invasion. White circles represent branching points, a gray-filled circle represents a repeller. **B**, **D** Direct numerical simulations demonstrate evolutionary branching in the system. The population densities of various strains were modeled using Eq. (7) along with the solutions to the system given by (4). We start with a single strain of boldness with *B* = 0.5. Panels (**A**, **B**) are constructed for *ϵ* = 0.1, panels (**C**, **D**) are constructed for *ϵ* = 0.5. All other parameters are as given in Table 1.

Next, using mathematical modeling, we address the fundamental question about a possible scenario of emergence of bold/shy strains as a result of evolution. In fact, evolution of boldness is largely related to expression of particular receptors in the brain of the fish, which is considered to be fast and energetically cheap process^23,24^. Boldness is related to other traits, such as exploration, defence, foraging, and other behaviors. Therefore, by considering evolution of boldness, we take into account evolution of the above mentioned traits, which substantially influence fitness and mortality. For the sake of simplicity, we assume clonal reproduction and implement the adaptive dynamics framework^25,26^ outlined in Supplementary Note 5 (see also Methods). We start with an initially monomorphic population characterized by a single boldness strain *B*. The expression for invasion fitness, measuring the success of mutations, is derived in Supplementary Note 5. Using the invasion fitness, we construct a Pairwise Invasibility Plot (PIP) to reveal any evolutionarily singular points. A typical PIP is shown in Fig. 5A. The figure shows two possible evolutionarily singularities at which the gradient of invasion fitness vanishes, the larger of which is an evolutionary repeller, the smaller of which is convergence stable but not evolutionarily stable, suggesting evidence of branching behavior whereby the initially monomorphic population becomes dimorphic and separates into shy and bold cohorts^27–29^.

We simulated the evolution of boldness, starting with a single strain of intermediate boldness *B* = 0.5. The evolutionary outcome is shown in Fig. 5B, where the vertical axis denotes evolutionary time, as represented by the succession of mutation events in the system. The boldness initially evolves as a monomorphic population towards the smaller evolutionary singularity (*B* = 0.2). However, having reached the neighborhood of this point, the monomorphic strain exhibits branching behavior, and two distinct branches of coexisting boldness strains emerge. Further evolution results in the bolder strain achieving its maximum boldness of 1, whereas the shyer branch evolves to the boldness of approximately *B* = 0.6. Note that this behavior is observed only when the initial boldness is less than the evolutionary repellor in Fig. 5A, otherwise, we observe a monomorphic population evolving to the maximal boldness. The mechanism seen here is possible for a small and intermediate, as compared to F^*^(0), numbers *N*. This suggests evolutionary branching as a possible explanation for the emergence of several strains in the population when the competition for shelters is strong. We checked the influence of the relation between mortality and boldness (measured by *ϵ*) on evolutionary branching. We found (see Supplementary Note 8, Figs. S22–S25) that branching behavior is possible for both negative and positive *ϵ*, as well as in the absence of such a relation (*ϵ* = 0).

In our simulations, the evolutionary branch with the highest boldness approaches its maximal possible level of *B* = 1. A more accurate (but more sophisticated) model should include negative effects of being ‘too bold’. For example, bold animals often have increased risk of predation due to underestimation of risk, also their reproduction rate may be reduced due to very frequent contests with conspecies^30^. This can be incorporated in the model by multiplying the corresponding term by a function, which would abruptly reduce the fitness close to the boundary *B* = 1. Our preliminary simulation shows that the evolutionary trajectory will never reach the critical boundary in this case. However, to avoid unjustified complexity, we prefer to use the original simplified model predicting the trajectory approaching the highest values of boldness.

For abundant sheltering (*N* is of the same order of magnitude as F^*^(0)), branching behavior does not occur. Instead, a single strain evolves towards the trait with maximal boldness; in this case, we observe a severe reduction in the equilibrium population F^*^ as in the monomorphic population. Even in the absence of a branching point, however, starting from a configuration of multiple initial strains can lead to the coexistence of bold and shy strains (e.g. Fig. 3). Thus, for large *N*, the system is evolutionary bi-stable, as the outcome will depend on the initial presence of strains.

### Empirical case study: interactions between rainbow trout *Oncorhynchus mykiss* and trematodes

**Fig. 6:**
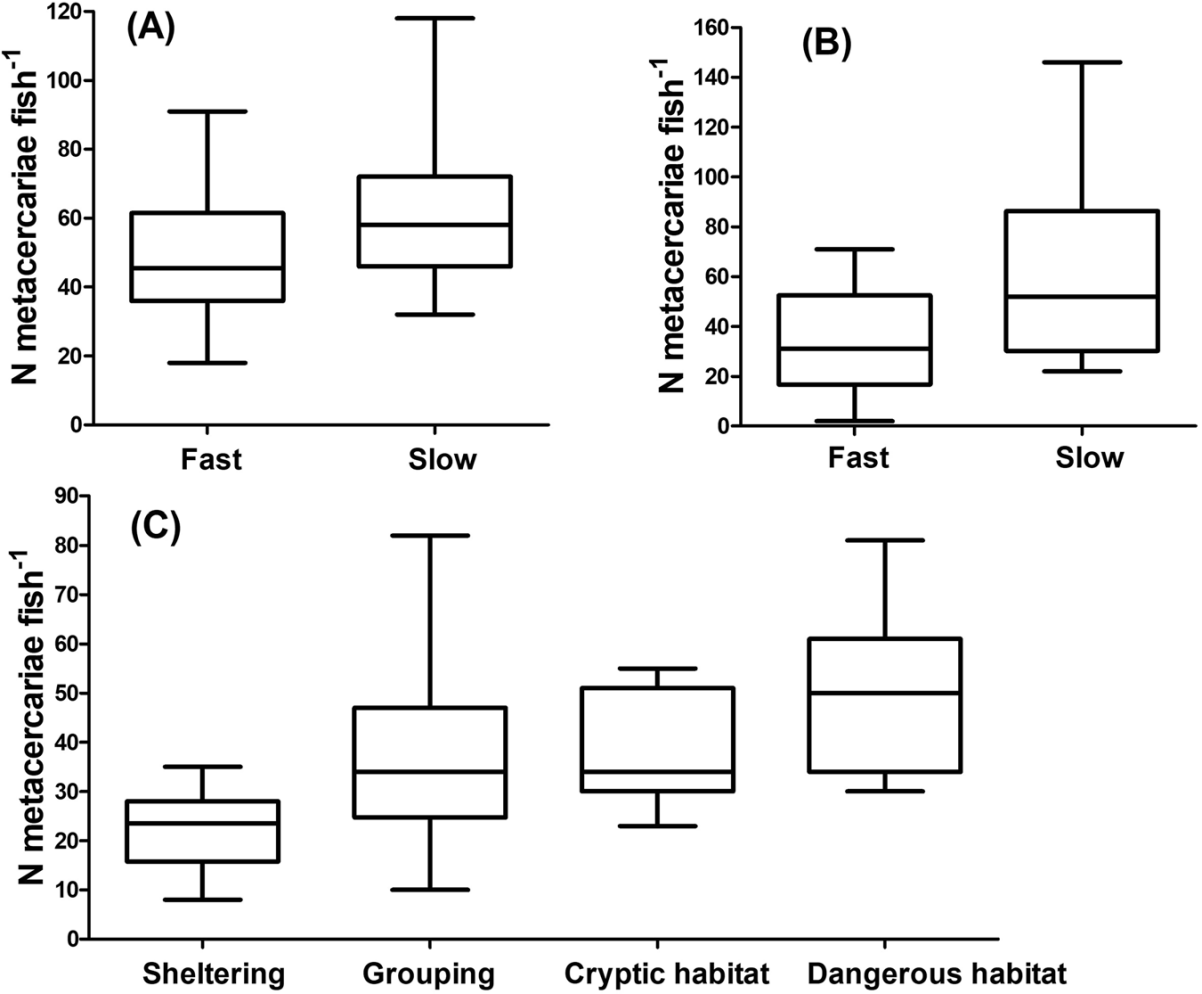
Experimental results (presented as box and whiskers plots) on interactions between rainbow trout *Oncorhynchus* and its parasite eye-fluke *D. pseudospathaceum*. **A** Isolated slow fish received more infection of *D. pseudospathaceum* than isolated fast fish (Mann-Whitney U-test, *p* = 0.013). **B** When kept in groups in a structured habitat (two interconnected compartments: one containing parasites, another free of them), slow fish received more infection (Mann–Whitney *U* test, *p* = 0.036). In both cases, fish were exposed to the same average concentration of parasites. **C** Infection load by *D. pseudospathaceum* metacercariae increases with an increase of threat anticipation by the fish. Sheltering (Sh)—solitary fish possessing a cover shelter. Grouping (Gr)—a group of 5 fish in a tank with a light bottom. Cryptic habitat (Cr)—solitary fish in a tank with a dark bottom. Dangerous habitat (Da) - solitary fish in a tank with a light bottom without shelter. ANOVA showed a pronounced effect of the anticipated threat on parasite acquisition (*p* < 0.0001). Pairwise comparisons (Tukey HSD test) showed that all pairs except Gr - Cr were significantly different (Da - Sh: *p* = 0.0001; Da - Gr: *p* = 0.015; Da - Cr: *p* = 0.050; Cr - Gr: *p* = 0.969; Cr - Sh: *p* = 0.001; Gr - Sh: *p* = 0.005). In total, 160 fish were used: 100 fish in the group test, 60 in the test with solitary fish: 20 with shelters, 20 without shelters and a dark-bottomed tank, 20 without shelters and a light-bottomed tank. For details on statistical analysis see Methods.
(ii) We consider four types of habitats with increasing levels of anticipated threat by fish (a description of the habitats can be found in Supplementary Note 3). Figure 6C shows that infection load with *D. pseudospathaceum* metacercariae gradually increases as fish anticipate more threats from a hostile environment, with the best defence behavior being to stay in a covered shelter. The smallest infection load is observed when individuals stay in a covered dark shelter, while parasite load increases for fish staying in a group in the open water and adopting shoal tactics. The largest parasite load is observed for solitary fish when the bottom is light. Our observations indicate that the gradual increase in parasite load is due to an increased stress response in the fish with higher anticipation of threat, resulting in a higher ventilation rate^31^.

A straightforward application of the above theory can be commercial fish farming, which is often affected by parasite contamination in the environment. We explore a particular case study of the interaction between rainbow trout and trematode parasites as well as the role of artificial shelters installed in fish farms. The biological system of rainbow trout *Oncorhynchus mykiss* and its trematode parasite, eye-fluke *Diplostomum pseudospathaceum*, is briefly discussed in the Methods section along with experimental design. Previous studies indicate a high cost of fish fighting for shelters^4^. Here we experimentally explore a few key aspects of this system which have remained unaddressed so far, namely: (i) whether parasite acquisition consistently varies between individual fish with permanent behavioral structuring (measured in terms of reactivity of individuals) infected individually and in groups, and (ii) difference in infection rates between fish possessing a shelter and groups in open water (this is related to dynamic behavioral structuring). Measuring the reactivity of individual fish is discussed in Supplementary Note 2. In short, fish were divided into two groups, ‘fast’ and ‘slow’; more reactive fish faster resumed their activity in a novel environment. Our novel experimental findings are the following:

(i) For both individual and group scenarios of infection, fast (more reactive) individuals received a lower, on average, parasite load compared to slow (less reactive) specimens. The results are, graphically, presented in Fig. 6A, B, where we show the total amount of parasites (measured in terms of the number of metacercariae in the eyes) received by each category of fish. We also present separately the outcome (intensity of parasitism) of the first and the second rounds of infection of each particular individual fish used in the experiments (see Supplementary Note 3, Fig. S3). This demonstrates an overall consistency of the vulnerability of individuals to parasite infection: there is a positive correlation between the number of parasites received in the first and second infection of the experiment.

From our empirical study, we conclude that (a) slow (shy) fish, on average, receive more parasites than fast (bold) fish. In other words, there is a negative correlation between reactivity and the parasite load. A possible explanation is that slow fish are more often and easily stressed by various biotic and abiotic factors, e.g., exposure to a new environment. The main mechanism is that when stressed, fish increase their ventilation rate and more cercariae can be transported through the gills^31^ (more detail are provided in the Discussion section). We found that (b) sheltering defensive strategy provides better protection against parasites as compared to shoaling. We show, as well, that (c) for an individual fish, vulnerability to parasite infection remains relatively constant over time.

The above experimental results, as well as some previous empirical research (see refs. ^4,31^), provide support for the implementation of our theoretical model to mimic rainbow trout-trematode interactions in fish farms. The parameters of the model are discussed in Methods. We should stress, however, that linking the experiments and the model requires the suggestion that an increased parasite load signifies increased mortality via predation. This suggestion is confirmed by the previously published evidence that parasites enhance the susceptibility of fish to predation (e.g. by piscivorous birds) through host manipulation (e.g.^32^). Therefore, we can conclude that the mortality in a shelter should be lower than that in the shoal (Δ_*S*_ > Δ_*T*_), and there is a negative correlation between parasite-induced mortality and the reactivity of individuals, interpreted as boldness (*ϵ* > 0). This information was used in parameterization of the theoretical model.

## Discussion

Our theoretical model shows that in a hazardous environment, the dynamical structuring of a population in terms of using different behavioral tactics, where one tactic requires access to some limited resource, may have negative consequences on the population’s survival. Even if the resource itself is highly beneficial to each user, insufficient level of this resource may have a negative impact at the population level due to strong intraspecific competition. Therefore, the provision of extra vital resources in insufficient amounts could act as poison for the proliferation and success of the population. Only in a very hostile environment, where population persistence would not be possible without adding a particular resource (e.g. safe zones) would the use of such a resource be justified. For fish-parasite interactions, the vital resource includes shelters which provide an individual with a physical defence against predators but also substantially reduce the stress of the organism^16,31^, which in turn, results in a lower infection load (see Fig. 6C). Therefore, the model predicts that installing an insufficient amount of shelters in fish farms will be counter-productive since it will increase the exposure of the population to natural enemies (parasites and predators).

We apply our theoretical model to explore the interaction between salmonids and their parasites. However, we also expect that similar mechanisms should be generic for other fish-parasite interactions. The main mechanism driving the intense infection of fish in a heterogeneous environment is the dynamical structuring of the population in terms of their antipredator behavior: using either shoaling or sheltering. The negative effect of dynamical structuring in fish is amplified by a ‘ghost’ of predation: individuals prefer shelters to the shoal even in the physical absence of predators, and when shelters provide a higher risk of infection than the surrounding environment^4^. Therefore, only in the case where a large proportion of the entire fish population in a farm is sheltered can one expect a positive response to the use of shelters. Alternatively, when the level of parasites in a fish farm is very low, adding shelters becomes always beneficial.

Another ecological application is the restoration of endangered natural fish populations in shallow water habitats, which serve as nursery grounds for various young fish species with flexible behavior, including salmonids in the freshwater phase. When an insufficient number of contestable resources (shelters, food patches) are introduced, fish start fighting for them, making themselves more vulnerable to natural enemies. Habitat enrichment is an important part of the restoration of nursery grounds for juvenile salmonids and marine fish^3,33–35^, thus our study suggests a hypothesis (to be tested empirically) that only an optimal pattern of habitat enrichment can facilitate the availability of vital resources to fish without increasing vulnerability to predators and parasites. The proposed theoretical framework can be used to explain the observed population density of some species of coral fish^21^, which contest refuges in corals. The model predicts that a gradual decrease in shelters due to the degradation of a coral reef would result in a severe drop in population density.

Finally, one may arguably apply the theoretical approach to modeling the social dynamics of humans, competing amongst each other for scarce jobs in certain prestigious companies and/or organizations (in place of shelters) in a hostile financial climate and under societal pressure. In this case, the shoal should be understood as a less desirable job or profession (e.g. a scholar can move from science to industry) due to disappointment and high competition with colleagues. Mortality should be understood as a person giving up their profession entirely (i.e. retirement or moving to a completely new career field).

Allowing for individual behavioral variability gives different outcomes to a single strain scenario. The detrimental effects on the population density due to an insufficient number of shelters are largely compensated for permanent behavioral structuring in boldness, or more generally, a behavioral syndrome around such structuring can efficiently mediate the negative effects of dynamical structuring in defense tactics. The key factor in the interplay between permanent and dynamical structuring is the boldness-based hierarchy of competitive dominance: shy individuals do not contest shelters occupied by bold ones, largely decreasing the number of contests for shelters and lowering the mortality of individuals staying in the shoal. This mechanism of mortality reduction seems to be generic, holding even when bold and shy individuals are only different in terms of their competitive dominance but are identical regarding their mortality rates. Our results lead us to conclude, however, that even for a population with distinct boldness cohorts, a sufficiently large number of shelters needs to be installed, or provided by the environment, before the population density will be effectively increased. For a polymorphic population, however, this critical number of shelters should be smaller than for a monomorphic one. The existence of a shy/bold axis of behavioral variation has been reported in a large number of species in their responses to different external stimuli, such as exploration for resources, mating, or avoidance of natural enemies^36–38^. In some fish species, boldness is amplified by the presence of predators and parasites but disappears when fish are in social and ecological isolation^6,39^. Our model predicts that increased boldness in the presence of natural enemies should act as a mechanism for reducing population mortality.

Previously, it was shown that the structuring of a population, in terms of boldness, can have a wide range of ecological consequences, including stationary population dynamics, such as stability or stationary densities^40,41^. Bolder behavior might stabilize the system through an increase in competition^40^, but it has also been suggested that bolder strains may destabilize an equilibrium due to their fast-changing dynamics^42^. Behavioral structuring may also have potential impacts on equilibrium density: interaction rates, such as fighting, may increase as bolder individuals are more active^40,43^, but intraspecific competition may also decrease if the distinct strains use different resources and habitats^41^. Kendall and collaborators^14^ considered a theoretical model describing the dynamics of a behaviorally heterogeneous population comprising two strains with different boldness. Their main conclusion was that in low-predation environments, the equilibrium abundance of the population will be smaller than in environments with higher predation. The main mechanism of the increase of the population density in a highly hostile environment seen in^14^ is a reduction in aggression by bold cohort individuals in the presence of large numbers of predators. Our simulation shows the opposite outcomes for variation of density with a gradual increase of parasite/predator levels in the environment. The mismatch between the model predictions can be potentially explained by the difference in model structures. We consider the same reproduction rate for all individuals, the reproduction kernel in our model is assumed to be frequency independent, and we assume a hierarchy of competitive dominance when modeling contests between individuals of different cohorts.

Previous studies revealed possible mechanisms for the emergence of diversity in animal personality traits, such as boldness, aggressiveness, or risk-taking behavior. Trade-offs between current and future reproduction potential have been shown to lead to behavioral structuring in the long term^44^. Another possible mechanism is the variability of metabolic rates for different individuals^45^. Behavioral structuring can be a consequence of a variable or noisy environment: differing behavioral traits can be a response to external stimuli, with some individuals being far more responsive to these changes than others^46,47^. A combination of both metabolic rates and responses to external stimuli has also been proposed to drive the emergence of behavioral traits, termed the Pace-Of-Life Syndrome (POLS) hypothesis: individuals with a fast POLS will grow faster and die earlier than those with a slower POLS due to a more intense metabolism and a higher mortality risk^48^. Finally, it has been suggested that individual differences in trust and trustworthiness can be explained by the feedback in cooperation among individuals driven by communication and ‘social awareness’^49,50^.

To the best of our knowledge, our system reveals a novel mechanism for the emergence and maintenance of behavioral structuring. The proposed scenario is the result of the interplay of three factors: (i) the spatial heterogeneity, generating two different types of anti-predator defence: shoaling and territorial behavior; (ii) strong intraspecific competition for a better spatial resource: shelters; (iii) the boldness hierarchy determines the outcome of competition for a shelter. The first two factors are amplified by a high level of natural enemies such as parasites and predators. Intensive intraspecific competition has, so far, rarely been considered a major factor in the coexistence of bold and shy strains in the literature. Importantly, the branching behavior, which results in the emergence of two or more types of coexisting strains, seems to be generic since it is observed for both *ϵ* > 0 and *ϵ* < 0, and in the case with *ϵ* = 0 (Supplementary Note 8, Figs. S22–S25).

Assume, for simplicity, that inheritance in the population is clonal. The main evolutionary force pushing the initially monomorphic population to the branching point (see Fig. 5) is strong intraspecific competition over shelters resulting in high overall mortality. The eventual switch to the dimorphic state results in a larger total population density as a consequence of the drop in the mean mortality rate within the population: competition for shelters becomes largely reduced. The mechanism assuring stable long-term coexistence of the distinct shy and bold cohorts is of particular interest since, regardless of the defence strategy used, shy cohorts have lower fitness than bolder ones. The main reason why shy individuals can still successfully persist and even reach high proportions in the total population is the emergence of density-dependent mortality in bold cohorts. Such density-dependent mortality occurs due to the heterogeneity of the environment: bold individuals in the shoal constantly challenge their bold conspecifics residing in shelters. This results in an extra mortality for bold cohorts, which is not suffered by shy individuals. Although possessing a higher fitness in a homogeneous habitat, bold individuals become victims of their superiority in the heterogeneous environment.

A key question is about sensitivity of our theoretical results to the variation of parameters and model functions. Extensive computer simulations showed that moderate deviation from the realistic default values of parameters listed in Table 1 does not alter our key findings (Supplementary Note 4). Therefore, the patterns presented in Figs. 1–5 are robust in the sense that they can be observed within a wide range of parameters. We should stress that here by the robustness we understand the occurrence of a pronounced (>10−20%) decrease in the population size F^*^ after adding shelters in the system (for a monomorphic population) and only small (<5%) variation in the fish numbers in the case of a polymorphic population. To explore the generality of our results, we also use analytical tools, which, for a monomorphic and a dimorphic populations, predict a drop in the population size after adding shelters (Supplementary Notes 6 and 8). Including the dependence of the per capita reproduction rate on boldness (as in^14^) does not affect the case of clonal reproduction, and one can prove this fact analytically in a similar way as in Supplementary Note 6. We found that for non-clonal reproduction, assuming a higher reproduction rate in bold individuals results in a reduction in the number of shelters required to have a beneficial effect for large *N*. However, at small and intermediate *N*, the generic pattern of the dependence F^*^(*N*) remains. Finally, we stress that our central assumption is that the boldness hierarchy determines the outcome of competition for a shelter, so by omitting this assumption the model would predict a distinct result, which is beyond the scope of this paper.

We also briefly checked the robustness of our model to the variation of the most uncertain model function, *ν*(*B*), which describes the dependence of the search rate on boldness. We considered the two following non-sigmoid forms: (i) *ν* = *B* (a linear function) and (ii) *ν* = *B*(1 + *B*_*μ*_)/(*B* + *B*_*μ*_) (a hyperbolic function). The results are partly provided in Supplementary Note 8, Figs. S18–21. We found that using these two non-sigmoid functional forms do not affect the previous results regarding the behavior of the total population size F^*^ for the polymorphic population. Also, simulations predict coexistence of bold and shy groups within a population. However, using non-sigmoid functional forms *ν*(*B*) predict different shapes of PIPs, in particular, we do not observe an evolutionary branching point for the hyperbolic function (see Supplementary Note 8, Figs. S26–S28). Therefore, the mutual coexistence of shy and bold strains for the scenario involving a decelerating function *ν*(*B*) should be explained via some other mechanisms, for example, via non-small genetic mutations, or others.

Our model of population dynamics operates on two different time scales, assuming an instantaneous exchange between the shoal and shelter compartment, and slow demographic processes (for modeling evolution we introduce a third, evolutionary time scale). Using a standard approach^51–53^, one can combine these two systems into a single model for variables *T*_*i*_ and *S*_*i*_ by multiplying the terms corresponding to demographic processes by some small parameter *ϵ*_0_ ≪ 1. When testing such a model, we found that provided *ϵ*_0_ < 0.1, the combined and reduced model predict similar results, which justifies the separation of time scales.

Among possible perspectives, we can cite the following. A notable extension would be modeling parasites as a dynamical variable rather than a static background variable, which would allow us to explore their role in regulating the population dynamics as well as the maintenance of behavioral structuring of the host population. The current model shows a polymorphic host population assures a higher parasitic transmission than a population with a single boldness strain. However, in a real-life ecosystem, the total amount of parasites should depend dynamically on the transmission rate making the resultant outcome uncertain. Another insightful perspective would be the inclusion of sexual dimorphism in behavioral traits such as boldness. An important feature of sexual dimorphism is that, even though individuals of a particular sex may not exhibit strong boldness, and thus not fight for shelter, they may have an essential influence on the distribution of behavioral traits and population dynamics through their offspring of the opposite sex. For example, in zebrafish *Danio rerio* males have significantly higher tendencies than females to adopt antipredator tactics in the presence of predators^54^. Finally, we should admit that for implementing the theoretical prediction to optimize fish farming one needs accurate values of parameters, in particular, this concerns mortality terms. This would require extensive experimental observation work, which we plan to conduct in the future.

## Methods

### Theoretical modeling framework

We developed a generic theoretical model to explore the role of intraspecific competition over a limited number of safe zones (shelters) in a behaviorally structured population in a hostile environment. The model is primarily focused on fish-parasite interactions. We consider that individuals within a population can adopt two anti-predator and anti-parasite tactics: (i) territoriality when they often reside within a shelter and guard it, or (ii) shoaling when they stay in a group. We stress that the shoaling compartment can be understood as an ensemble of a large number (e.g. hundreds) of independent shoals, which are homogeneously spread over space. The transition between the two behavioral states occurs via a short-term solitary phase, not modeled explicitly. We assume that the territorial strategy is more beneficial as it results in less mortality, as is the case study of the trout-parasite system.

Along with dynamic behavioral structuring (shoaling/sheltering), we consider a permanent behavioral structuring according to the level of boldness of each individual, assuming that boldness is positively correlated with reactivity, exploration, aggressiveness and risk-taking ability^9,55–57^. For example, in the empirical trout study, the fast fish would correspond to bold strains, whereas the slow ones would be less bold, i.e. shy strains. For each of the *n* different behavioral strains, we quantify the level of the boldness of strain *i* by the parameter *B*_*i*_, where 0≤*B*_*i*_≤1, with 1 being the highest possible boldness: this approach is used in the literature^30^. The existence of the highest possible boldness is due to physiological constraints for the life history traits of the given species. Each strain *i* can adopt two tactics, territoriality and shoaling, in numbers denoted by *T*_*i*_ and *S*_*i*_, respectively. These two components make up the population density of the strain *i*, i.e. *F*_*i*_ = *T*_*i*_ + *S*_*i*_. The total number of shelters, *N*, is fixed, and we assume that they are always fully occupied by some individuals, so the total number of individuals adopting territorial tactics is $T\equiv \mathop{\sum }\nolimits_{j = 1}^{n}{T}_{j}=N$.

An important feature of our model is the consideration of two distinct time scales corresponding to different types of processes: changes in the dynamical behavioral state of individuals (shoal/shelter) take place on a fast time scale, whereas demographic processes such as reproduction and mortality take place on a slow time scale. This approach of time scale separation is well-known in ecological modeling^51–53^. Note that evolutionary modeling adds an extra (third) time scale (see the end of this section).

First, let us consider the fast time scale on which the population density of each strain *F*_*i*_ remains constant. The exchange rate of individuals between *T*_*i*_ and *S*_*i*_ is described by the following function *M*_*i*_

1 $${M}_{i}=\mathop{\sum }\limits_{j=1}^{n}\left({S}_{i}I({B}_{i},{B}_{j},{T}_{j})-{S}_{j}I({B}_{j},{B}_{i},{T}_{i})\right).$$

The first term in the above expression describes individuals who enter a shelter from the shoal by successfully invading an occupied shelter. After the invasion of a shelter, the defeated shelter dweller loses its shelter and returns to the shoal, described by the second term of the equation. The function *I*(*B*_*i*_, *B*_*j*_, *T*_*j*_) is the rate at which an individual from the shoal with boldness *B*_*i*_ displaces an individual with boldness *B*_*j*_ occupying a shelter after a contest given that *T*_*j*_ individuals with boldness *B*_*j*_ are currently occupying shelters, the formulation of which is given further in Eq. (5). The fast exchange of individuals between shoals and shelters can be modeled by

2 $$\begin{array}{l}\frac{{{{{{\rm{d}}}}}}{T}_{i}}{{{{{{\rm{d}}}}}}t}={M}_{i};\quad \frac{{{{{{\rm{d}}}}}}{S}_{i}}{{{{{{\rm{d}}}}}}t}=-{M}_{i}.\end{array}$$

We assume that this process occurs instantaneously in comparison to the slow demographic processes. As such, we can fix the population sizes *F*_*i*_, so the values of *T*_*i*_ and *S*_*i*_ are given by the stationary state of (2) and are functions of *F*_*i*_, i.e. (*T*_*i*_(*F*_*i*_), *S*_*i*_(*F*_*i*_)), with the condition *T*_*i*_(*F*_*i*_) + *S*_*i*_(*F*_*i*_) = *F*_*i*_. The stationary states of (2) are determined by *M*_*i*_ = 0. Our numerical simulation shows that all stationary states are stable, see Supplementary Note 7, Fig. S5. They can be found by solving the following *n-*dimensional system

3 $${M}_{i}=0=({F}_{i}-{T}_{i}({F}_{i}))\mathop{\sum }\limits_{j=1}^{n}I({B}_{i},{B}_{j},{T}_{j}({F}_{j})) \\ -\mathop{\sum }\limits_{j=1}^{n}({F}_{j}-{T}_{j}({F}_{j}))I({B}_{j},{B}_{i},{T}_{i}({F}_{i}))$$

for *i* = 1: *n*, this follows directly from (1) and with *S*_*i*_(*F*_*i*_) = *F*_*i*_ − *T*_*i*_(*F*_*i*_). This can be simplified by setting ${T}_{n}({F}_{n})=N-\mathop{\sum }\nolimits_{j = 1}^{n-1}{T}_{j}({F}_{j})$, which follows directly from $T\equiv \mathop{\sum }\nolimits_{j = 1}^{n}{T}_{j}=N$, to following *n* − 1 dimensional system

4 $${M}_{i}= 0=	({F}_{i}-{T}_{i}({F}_{i}))\left(\mathop{\sum }\limits_{j=1}^{n-1}I({B}_{i},{B}_{j},{T}_{j}({F}_{j}))+I({B}_{i},{B}_{n},N-\mathop{\sum }\limits_{j=1}^{n-1}{T}_{j}({F}_{j}))\right)\\ 	-\mathop{\sum }\limits_{j=1}^{n-1}({F}_{j}-{T}_{j}({F}_{j}))I({B}_{j},{B}_{i},{T}_{i}({F}_{i}))-({F}_{n} \\ 	-(N-\mathop{\sum }\limits_{j=1}^{n-1}{T}_{j}({F}_{j})))I({B}_{n},{B}_{i},{T}_{i}({F}_{i}))$$

for *i* = 1: *n* − 1. Once the system is solved for *T*_*i*_(*F*_*i*_), *i* = 1: *n* − 1, we can determine ${T}_{n}({F}_{n})=N-\mathop{\sum }\nolimits_{j = 1}^{n-1}{T}_{j}({F}_{j})$ and, finally, *S*_*i*_(*F*_*i*_) = *F*_*i*_ − *T*_*i*_(*F*_*i*_).

We parameterize the function *I*(*B*_*i*_, *B*_*j*_, *T*_*j*_) as follows

5 $$I({B}_{i},{B}_{j},{T}_{j})={I}_{0}\nu ({B}_{i})\omega ({B}_{i},{B}_{j}){T}_{j},$$

where *I*_0_ describe the maximal rate at which a shoal individual encounters a single shelter, the term *ν*(*B*_*i*_) the role of boldness in the search for shelters (this function is discussed below). It is well-known that contests between a shy invader and a bold shelter occupant are usually rare in nature^58–62^. Therefore, we introduce a conditional probability *ω*(*B*_*i*_, *B*_*j*_) for shoal individuals with boldness *B*_*i*_ to invade a shelter occupied by an individual with boldness *B*_*j*_, provided the invader has already approached the shelter and visually assessed the boldness of the shelter’s occupier. Within this study, a sigmoid function is considered, defined as

6 $$\omega ({B}_{i},{B}_{j})=\frac{{e}^{-{\delta }_{\omega }({B}_{j}-{B}_{i})}}{1+{e}^{-{\delta }_{\omega }({B}_{j}-{B}_{i})}}.$$

In the case where *B*_*i*_ and *B*_*j*_ substantially differ from each other and *B*_*i*_ < *B*_*j*_ (a shy invader and a bold occupier), *B*_*i*_ will almost never attempt an invasion, so *ω*(*B*_*i*_, *B*_*j*_) ≈ 0. For *B*_*i*_ > *B*_*j*_ (a bold invader and a shy occupier), *B*_*i*_ will almost always attempt an invasion, so *ω*(*B*_*i*_, *B*_*j*_) ≈ 1. For *B*_*i*_ ≈ *B*_*j*_ we have *ω*(*B*_*i*_, *B*_*i*_) ≈ 0.5. 1/*δ*_*ω*_ gives the width of the transition layer, which is assumed to be narrow. Therefore, the considered sigmoid form with a sharp transition (a smooth version of a step-wise function) is a natural way to describe the absence of contests between a shy invader and a bold occupier.

On the slow demographic time scale, individuals reproduce and suffer mortality (either natural background mortality or due to parasitism or predation). The demographic model for the population density of the strain *i* is given by

7 $$\frac{{{{{{\rm{d}}}}}}{F}_{i}}{{{{{{\rm{d}}}}}}t}= 	b\left(\mathop{\sum }\limits_{j=1}^{n}{F}_{j}\right)R({B}_{i},{{{{{{{\bf{F}}}}}}}})-{m}_{0}{F}_{i}-\Delta {m}_{S}({B}_{i}){S}_{i}({F}_{i}) \\ 	-\Delta {m}_{T}({B}_{i}){T}_{i}({F}_{i})\\ 	-G({B}_{i},{{{{{{{\bf{S}}}}}}}}({{{{{{{\bf{F}}}}}}}}),{{{{{{{\bf{T}}}}}}}}({{{{{{{\bf{F}}}}}}}})),$$

where the bold symbols denote vectors with components corresponding to all strains (i.e. **F** = [*F*_1_, . . . *F*_*n*_], **T**(**F**) = [*T*_1_(*F*_1_), . . . , *T*_*n*_(*F*_*n*_)] and **S**(**F**) = **F** − **T**(**F**)).

The first term in (7) accounts for reproduction, where the kernel *R*(*B*_*i*_, **F**) governs the redistribution of offspring, as it is explained in detail below; $b(\mathop{\sum }\nolimits_{j = 1}^{n}{F}_{j})$ is described by the logistic function (of the form ${b}_{0}\left(1-\frac{\mathop{\sum }\nolimits_{j = 1}^{n}{F}_{j}}{K}\right)$). We do not consider age structuring so that the equations remain tractable. The next three terms account for mortality. *m*_0_*F*_*i*_ is the background mortality; the other rates stand for the additional mortality due to residing within the shoal and shelter (Δ*m*_*S*_(*B*_*i*_)*S*_*i*_(*F*_*i*_) and Δ*m*_*T*_(*B*_*i*_)*T*_*i*_(*F*_*i*_)). The last term *G*(*B*_*i*_, **S**(**F**), **T**(**F**)) represents extra mortality caused by competition for shelters. Individuals from the shoal constantly attempt to invade an occupied shelter, engaging in fights with shelter residents. For strain *i*, the number of contests for shelters per unit of time by individuals currently staying in a shoal is proportional to the sum of *S*_*i*_*I*(*B*_*i*_, *B*_*j*_, *T*_*j*_) over all possible types of residents *B*_*j*_ of the shelters. The mortality term due to contesting shelters is given by

8 $$G({B}_{i},{{{{{{{\bf{S}}}}}}}},{{{{{{{\bf{T}}}}}}}})={m}_{p}({B}_{i})\left(\nu ({B}_{i}){S}_{i}\mathop{\sum }\limits_{j=1}^{n}\omega ({B}_{i},{B}_{j}){T}_{j}+D{T}_{i}\mathop{\sum }\limits_{j=1}^{n}{S}_{j}\nu ({B}_{j})\omega ({B}_{j},{B}_{i})\right).$$

The term *m*_*p*_(*B*_*i*_) = *ν*_*μ*_(1 − *ϵB*_*i*_) describes the extra mortality rate due to fighting (counted per a single individual); *ν*_*μ*_ is the coefficient, combining effects of the cost of fighting and the search rate for shelters; the multiplier (1 − *ϵB*_*i*_) takes into account the dependence on the boldness in parasite acquisition (using a linear functional form is done for simplicity purposes); *ν*(*B*_*i*_) takes into account the role of boldness in the search of shelters (similar to the term *I*(*B*_*i*_, *B*_*j*_, *T*_*j*_)).

The parameter *D* < 1 describes the reduction in the cost of fighting when defending a shelter: the invader usually suffers a high parasitic cost, whereas the defending resident suffers a reduced cost. The requirement that *D* < 1 for the owners of shelters has empirical justification. For fish-parasite interactions, when fighting more aggressive dominants usually receive fewer parasites than the subordinates, which is related to differences in ventilation rate: elevated ventilation results in an increased infection rate^31^. Social status often affects ventilation rate via different mechanisms^16,63,64^. For example, the low social status of subordinate fish often experiences more stress which increases their metabolic rate^65^, while the dominants, as owners of the shelter, have lower maintenance metabolism^66^.

To describe relation between boldness *B* and extra mortality rates Δ*m*_*S*_(*B*_*i*_) and Δ*m*_*T*_(*B*_*i*_) we use the following linear parameterizations

$$\Delta {m}_{S}({B}_{i})={\Delta }_{S}(1-\epsilon {B}_{i});\Delta {m}_{T}({B}_{i})={\Delta }_{T}(1-\epsilon {B}_{i}),$$

where Δ_*S*_ and Δ_*T*_ are coefficients that depend on the level of natural enemies (predator or parasite) in the environment. The dimensionless parameter *ϵ* describes the variation of mortality rates with an increase in boldness. Positive values of 0 < *ϵ* < 1 signify a decrease of mortality with boldness; this scenario occurs in the here-considered case study of trout-parasite interactions (rough estimation based on our empirical study gives *ϵ* ∈ (0.075, 0.6), see the next subsection below). Some other biological systems also show a similar correlation^67^. On the other hand, it was reported that few other biological systems may show a positive correlation between mortality and boldness^14,30^. We also considered the cases, where *ϵ* = 0. Finally, we must stress that using a linear functional form was done for simplicity purposes only to be able to connect the model with our empirical data on trout-parasite interactions.

Following some previous studies^68^, the reproduction kernel *R*(*B*_*i*_, **F**) that describes the redistribution of offspring *B*_*i*_ around the boldness trait of the parent *B*_*j*_ is modeled using the Gaussian law. This way is used in mathematical modeling in quantitative genetics^69^

9 $$R({B}_{i},{{{{{{{\bf{F}}}}}}}})=\mathop{\sum}\limits_{j}{A}_{j}{F}_{j}\exp \left(-\frac{{({B}_{j}-{B}_{i})}^{2}}{{D}_{w}}\right),$$

where *A*_*j*_ is the normalizing constant, and *D*_*w*_ is the width of the kernel which determines the heritability of boldness. In particular, in the extreme cases where *D*_*w*_ ≫ 1, we have uniform mixing, whereas for *D*_*w*_ ≪ 1 we have clonal reproduction.

We assume that the dimensionless function *ν*(*B*), accounting for boldness in the search for shelters, is described via the sigmoid function

$$\nu (B)=\frac{{B}^{\mu }}{{B}^{\mu }+{B}_{\nu }^{\mu }}.$$

Here *μ* > 2 and 0.1 < *B*_*ν*_ < 0.7 are model parameters. Very shy individuals do not attempt to invade occupied shelters, with *ν*(0) = 0, while *ν*(*B*) is an increasing function of *B* with an inflection point at an intermediate value of *B*, so that individuals with maximal boldness *B* = 1 attempt to invade at a rate close to 1. Along with the above sigmoid function, we briefly explored the model predictions for two more scenarios, in particular for *ν*(*B*) = *B*, *ν*(*B*) = *B*(1 + *B*_*ν*_)/(*B*_*ν*_ + *B*), which are a linear and a hyperbolic functions, respectively.

For our system comprising trout and its parasites, we estimated model parameters using available empirical data (see the next subsection below). We summarize the meanings of all model variables, functions, and parameters, as their values and units in Table 1.

In the case where a single strain of a certain boldness is present (i.e. the population is monomorphic), the model equation for the total population density *F* substantially simplifies to become

$$\frac{{{{{{\rm{d}}}}}}F}{{{{{{\rm{d}}}}}}t}= 	b(F)F-\left(mF+\Delta {m}_{S}(F-N)+\Delta {m}_{T}N\right)\\ 	-{m}_{p}(F-N)N(1+D)\nu /2.$$

In Supplementary Note 4, it is shown how the above equation can be obtained directly using similar steps of reasoning as for the polymorphic population.

We also study the evolution of boldness using the adaptive dynamics framework^25,26,70^, which considers the long-term evolutionary outcome of the invasion of a rare mutant with boldness into the environment formed by a resident. The outcome is characterized by invasion fitness (defined as the long-term average growth rate of a rare invading mutant), where positive fitness indicates a successful invasion, with the mutant displacing the resident. This process occurs iteratively, with successive mutant invasions which, when successful, exclude the resident^27–29,70^. Pairwise Invasibility Plots^71^ (PIPs) are graphical illustrations of the invasion success of potential mutants, displaying all the mutant traits for which the invasion fitness is positive, i.e. a successful invasion, for each resident. These PIPs suggest the subsequent evolutionary behavior of an evolutionary singularity^27,29^. The singularities can either be stable (an evolutionary attractor), unstable (an evolutionary repellor), or a branching point. We stress that evolution modeling involves adding an extra timescale, the evolutionary time scale, which is much slower than the demographic one. More information on the adaptive dynamics framework is provided in Supplementary Note 5.

The corresponding computer codes for numerical simulations are available in ref. ^72^.

### Biological system and experimental design

Rainbow trout, *Oncorhynchus mykiss*, a commonly farmed fish across the globe, is known to serve as a suitable intermediate host for a trematode *Diplostomum pseudospathaceum*^73,74^. The entire life cycle of the parasite is presented in Supplementary Note 1, Fig. S1. Cercariae of *D. pseudospathaceum* mainly infects fish by entering through the gills^31^. This parasite is not horizontally transmitted between individual fish. After infection, metacercariae grow and develop in the lenses of the fish’s eyes. Mature, infective metacercariae produce alterations in host behavior, making fish more vulnerable to predation by piscivorous birds, the final hosts of *D. pseudospathaceum*^18,32,75^. The rate of infection can be quantifiable by the number of metacercariae in the eye lenses, which will be positively correlated to an increase in mortality from predation^18,32,75^.

The fish specimens of rainbow trout used in our experiments were obtained from a commercial fish farm in Finland, where they were reared in indoor tanks supplied with groundwater and were free of *D. pseudospathaceum* infection. The mean ± s.d. fish fork length (FL) of Young-Of-the-Year (YOY) fish was 86.4 ± 9.2 mm. FL is the length of the fish measured from the tip of the snout to the end of the middle caudal fin rays. Prior to the experiments, about 400 fish were kept in a flow-through tank of 2.5 m^3^ on 15:9 L : D cycle at 15–16 °C; fed with commercial pelleted food (1.5 mm size, Nutra Parr LB, Norway).

Cercariae of *D. pseudospathaceum* were obtained from 14 naturally infected *Lymnaea stagnalis* snails (the first intermediate host of the parasite) collected from Lake Konnevesi. *D. pseudospathaceum* is the only diplostomid species found in this snail in Lake Konnevesi^76,77^. Snails were kept in a refrigerator at 7 °C and transferred to the laboratory conditions (at 18 °C, daylight) to induce cercariae production. We pooled all cercariae produced within 6 h and estimated their density from ten 1-ml subsamples of the suspension. The infectivity of *D. pseudospathaceum* cercariae did not decrease even 10 h after shedding at 20 °C^78^.

Before exposure to parasites, 120 fish were sorted according to their behavioral trait which we define as ‘reactivity’. Fish were placed individually into a novel compartmentalized tank, and the time before fish moved from one compartment to another was recorded. If the fish stayed longer than 30 min in the initial compartment, they were unused in further experiments. Fish were considered ‘fast’ (more reactive) if it took them 5 or fewer minutes to move to a different compartment, and ‘slow’ (less reactive) if they stayed longer. Overall, 55 fish were considered as ‘fast’, and 57 as ‘slow’. The frequency distribution of the reaction time, i.e. the time before a fish moved to the other compartment, across the experimental fish population, is presented in Supplementary Note 2, Table S1.

Experiments were conducted at the Konnevesi Research Station, University of Jyväskylä, in July-August 2012 (Exp. 1) and August 2013 (Exp. 2) to answer the questions below. Descriptions of the experimental settings are provided below. For the raw data in each experiment see Supplementary Note 3, Tables S2–S5.

(Exp.1) Infection rates in fast and slow fish. Fish from both groups were infected individually in 2-liter tanks and then kept in separate 10-L flow-through tanks. 40 fast and 40 slow fish were exposed to parasites at the concentration of 200 cercariae per liter for 15 min. To check whether individual vulnerability to parasites was consistent, we exposed fish to the same concentration of cercariae 12 days after the 1^st^ infection. 20 fast and 20 slow fish (different individuals from the previous experiment) were used. Parasites acquired during the 1^st^ and 2^nd^ infection were recognized by their size, morphology and motility. We used a structured habitat and fish in groups to better understand the differences in behavior, i.e. individual variation in their ability to avoid parasitized areas (Supplementary Note 3). We used a homogeneous environment to explore innate physiological (non-behavioral) differences of individual fish in terms of their vulnerability to infection.

(Exp.2) Variation of infection burden of fish under different anticipated threats. In all experiments, fish were threatened by the novelty of the environment, which is known as an efficient stressing factor^79–81^. To assess the vulnerability of fish to *D. pseudospathaceum*, we compared infection rates in fish demonstrating either territorial or grouping behavior with that of a solitary fish deprived of shelters and conspecifics. Solitary fish were tested over either a dark or light bottom. The white background is assumed to be more stressful than the dark one over which fish are cryptic^18^. We tested individual fish or a group of 5 fish in the open field arena and individual fish in a tank with a covered shelter on the bottom. The tests on solitary fish were carried out in 10 l light or dark plastic tanks and light tanks with cover shelter on the bottom. Groups of fish were tested in 50 l light plastic tanks. Fish were exposed to 100 ind l^−1^*D. pseudospathaceum* cercariae for 20 minutes after 30 minutes of acclimation. Twenty replicates of each of the trials were completed. In each experiment, we used random (unsorted) mixed groups of fish, in terms of their reaction time. (for details see Supplementary Note 3, Table S5)

The choice of the group size (4 to 5 fish) in the above experiments was related to the number of individuals necessary to initiate social relations^82^ in the tank of 0.51 m^2^ bottom and volume of 180 *l*. Such fish densities of YOY salmonids are typical of nursery grounds^83^ and experimental studies^84^. Too high or too low density could provoke abnormal behavior^82^. The chosen concentration of parasites is commonly used in experimental infections of salmonid fish^32^. This concentration is within the range reported in a natural environment^85,86^.

### Estimation of model parameters

Here we estimate model parameters for the considered biological system of rainbow trout and its parasites. Note that in the case where we could not find the accurate values of parameters for the above-mentioned system, we consider broader data ranges available for salmonids, or other fish families.

We consider a habitat with the area of 10^4^ m^2^ = 1 ha. For simplicity, we assume that the habitat has a square shape. This corresponds to the estimate for the carrying capacity to be *K* = 10^4^ *individuals* of fish (of all age categories) in absolute numbers within the considered area^87,88^. We allow *K* to vary within the range of 10^3^ < *K* < 2.5 ⋅ 10^4^ *individuals*.

The reproduction rate for salmonids can be estimated from the data on the recruitment/stock ratio^89–91^. Empirical observation provides the following range for the ratio between the number of age 1 rainbow trout individuals and the number of fish in the stock: 5–150 (measured at low stock density). Assuming that reproduction occurs annually, this signifies for the annual growth rate *b*_0_ to vary within 1.6 < *b*_0_ < 5 year^−1^.

The natural mortality *m*_0_ of the rainbow trout can be estimated to vary from 0.6% to 2% per month^92^. This gives the estimated mortality rates to be approximately 0.07 < *m*_0_ < 0.25 year^−1^. Note that similar mortality values are reported for the Atlantic salmon^92^.

The mortality due to parasites and predators is more variable, and its value largely depends on the abundance of natural enemies. For salmonids, recapture data estimate the ratio between the parasite/predator-induced mortality and the natural mortality to vary between 1.5 and 50^93–95^. This ratio is highly variable throughout the year, therefore a more correct estimation should include averaging over the period of observation, which reduces the upper bound to approximately 10. For the non-sheltering (shoaling) fish, this gives an approximate range of 0.1 < Δ_*S*_ < 2.5 ye*ar*^−1^. The mortality due to parasites and predators for the sheltering fish can be roughly estimated using the empirical results of the current study, showing that the parasite load for the sheltering fish is 1.5–2 times smaller as compared to the shoaling individuals. Also, we should keep in mind that sheltering individuals will have a smaller predation risk. This gives the following range 0.05 < Δ_*T*_ < 1.6 year^−1^.

The reduction in the cost of fighting, when defending a shelter, described by the dimensionless parameter *D* can be estimated as follows. It is known that residents (shelter owners) are less stressed and more familiar with surroundings than invaders, i.e. 0 < *D* < 1^66^. Residents are known to be more efficient combatants, which spend less energy and have lower ventilation rates. The latter allows them to decrease infection rate, compared to intruders by a factor ranging from 0.4 to 0.9^4^. By assuming mortality rates to be proportional to the infection load in the fish, we have *D* = 0.4. However, we allow the parameter *D* to vary in a broader range: 0.2 < *D* < 1.

The parameter *ϵ*, which accounts for the difference in mortality/parasite acquisition, is hard to estimate. Our empirical data, presented in this study (Fig. 6A, B), show that this parameter is positive for the considered experimental settings, signifying that parasite acquisition decreases with boldness. Unfortunately, the obtained data do not allow us to reveal the functional dependence between *ϵ* and the mortality rates, therefore, here we suggest the simplest linear relationship between the mortality and the boldness. The ratio of acquired parasites between the bold and the shy groups may largely vary from 0.075 to 0.6. We assume that within the considered ranges of parasite concentration, the mortality rate is proportional to the number of parasites in the fish, in this case, *ϵ* ∈ (0.075, 0.6). However, we also briefly consider negative values (see Supplementary Note 4), as it was reported that few systems (non-rainbow-parasite systems) show a positive correlation between mortality and boldness^14^. We also consider the case of *ϵ* = 0 to see how important is the dependence of the boldness on mortality for the generality of our modeling results (see Supplementary Note 4).

The dimensionless function *ν*(*B*), relating the boldness *B* and the rate of search of shelters, is not well documented not only for salmonids but for other fish families. Various measurements of fish boldness exist in the literature, for example, boldness is often measured based on the time needed to emerge from a cover in experiments^30,96^. It was reported that bolder fish individuals are more active and persistent in their search^97,98^ and this is related to the so-called behavioral syndrome^99^. Our measurements of the time of rainbow trout individuals staying undercover show a wide range of values, in particular, we can see examples of staying in the cover for a long time (see Supplementary Note 2, Fig. S2). This is also observed in other fish species^30^. Therefore, we can assume that for search rate by a very shy individual (*B* ≈ 0), we have *ν*(0) ≈ 0. On the other hand, common sense reasoning tells us that at very high values of boldness, the exploration efficiency should decelerate. This can occur for several reasons. For example, bold individuals may be extra persistent in staying for a longer time in a particular location and exploring nearby objects^100^, whereas a more efficient search strategy would be to continue moving across the entire habitat by following other (shyer) individuals. Finally, some studies show that the exploration propensity in fish shows acceleration within the intermediate range of boldness^30,97^. Using the above reasoning, we considered the following sigmoid function: $\nu (B)=\frac{{B}^{\mu }}{{B}^{\mu }+{B}_{0}^{\mu }}$, where *B*_0_ and *μ* > 1 are positive dimensionless parameters. For *B*_0_ we require to be close to the mid-point of the entire boldness range (*B*_0_ = 0.5); however, we also include other values: 0.2 < *B*_0_ < 0.8. In this case, we have *ν*(1) close to one. For *μ*, we are required to stay within the range 2 < *μ* < 8 to avoid a too sharp switch between the accelerating and the decelerating parts of the curve. Due to the mentioned uncertainty in the shape of the function *ν*(*B*), we also tested two other functional forms of *ν*(*B*), in particular, the linear dependence *ν*(*B*) = *B*, and the Monod-type hyperbolic function $\nu (B)=\frac{B(1+{B}_{0})}{B+{B}_{0}}$ (we have *ν*(1) = 1). Using those non-sigmoid parameterizations allows us to explore the generality of our results to other biological systems.

We proceed to estimating the key parameter *ν*_*μ*_, which quantifies the extra mortality due to fish fighting for a shelter. This parameter incorporates the search rate for a shelter as well as the amount of parasites acquired by individuals due to contesting shelters. We must say that it is hard to obtain an accurate value for *ν*_*μ*_, therefore, we are only able to provide some rough estimates. We assume that the mortality of infected fish is proportional to the amount of parasites acquired. For simplicity, we assume that each individual fish within the shoaling fraction has the same parasite load for the same value of boldness *B*. The same concerns the sheltering fraction of fish (note that the shoaling and the sheltering fractions may differ in terms of parasite loads). We assume (based some empirical facts, see below) that the parasite load in the shoaling or sheltering fish, measured as the number of metacercariae per individual, can be estimated using a simple differential equation

10 $$\frac{{{{{{\rm{d}}}}}}P}{{{{{{\rm{d}}}}}}t}=r+{r}_{1}-\alpha P,$$

where *P* is the number of metacercariae per individual, *r* is the maximal rate of acquisition of parasites by an individual in the absence of contests for shelters; *r*_1_ is the maximal rate of acquisition of parasites by an individual due to contesting shelters. The term *αP* describes the effects of saturation: it is known that an increase in the numbers of metacercariae inside a host reduces the rate of acquisition of new parasites^101,102^, *α* is a positive parameter. Note that for the shoaling and the sheltering fish, the parameters *r*, *r*_1_, *α* can differ. It is easy to show that at equilibrium (*dP*/*dt* = 0), the number of metacercariae per fish P^*^ is given by

$${P}^{* }=\frac{r+{r}_{1}}{\alpha }.$$

Empirical estimates provide the following values of *r* in an infected environment^4^: *r* = 5 *metacercariae/day* or *r* = 5 ⋅ 365 *metacercariae/year*. It is also known that the cost of a single fight is estimated to be about 10 or 15 metacercariae per fish^4^. To find *r*_1_, one needs to estimate the frequency of fighting for a single shelter by a single fish. We are unaware of direct empirical observation of the frequency of fighting of rainbow trout within the considered spatial area. Therefore, we use a combination of modeling and the existing data.

We first assume that the search can be described by a simple uncorrelated random walk, often used in ecological modeling^103^. More sophisticated patterns of spatial motion can be considered as well as an extension of our simple approach. The corresponding equations of spatial motion of fish are given by:

$$X({t}_{i+1})=X({t}_{i})+{\Delta }_{x}({t}_{i}),$$

$$Y({t}_{i+1})=Y({t}_{i})+{\Delta }_{y}({t}_{i}),$$

where *X*(*t*_*i*_) and *Y*(*t*_*i*_) are the spatial coordinates of the considered individual at time *t*_*i*_; Δ_*x*_(*t*_*i*_) and Δ_*y*_(*t*_*i*_) are the spatial increments of individual’s position, when moving from time *t*_*i*_ to *t*_*i*+1_. For the uncorrelated random walk, we have ${\Delta }_{x}({t}_{i})=L({t}_{i})\cos (\phi ({t}_{i}))$, ${\Delta }_{y}({t}_{i})=L({t}_{i})\sin (\phi ({t}_{i}))$, with *L*(*t*_*i*_) being a Gaussian random variable (the length of the spatial displacement) and *ϕ*(*t*_*i*_) being the angle of the direction of movement, which is uniformly distributed in the range of [0, 2*π*]. *L*(*t*_*i*_) is described by a normal law with a zero mean and the standard deviation *σ*.

We should stress that the above simple movement model accounts for the fact that a fish individual spends a large proportion of time in some shoal (or multiple shoals): fish can switch between shoals by leaving it during the daytime or joining a new shoal at dawn (since every evening, shoals disappear to re-emerge the next day with possibly different new members).

The value of *σ* can be estimated based on the experiments with the release of trout with a further recapture^104^: in 1-2 month time after a release of fish, the mean squared displacement can be estimated to very from 50 to 200 *m*. This gives an estimate for *σ* in the random walk simulations to be approximately 0.5*m* < *σ* < 3*m* (provided the time step between change of directions is approximately 20-30 min).

We further estimate the frequency of finding a (single) shelter by a fish when moving randomly within the considered spatial area with impenetrable boundaries. In our modeling, we set the maximal distance for the fish to see the shelter to be 1.5 − 2m, which has empirical evidence^87,101^. We put a single shelter at the center of the habitat, however, this is not crucial for our estimates. In our simulation, a fish starts its search from some randomly chosen starting point. We simulated our system for a long time (corresponding to several years) from 1000 different starting points. We found that the frequency of finding a single shelter varies from 4 to 28 times per year for the habitat of size of 10^4^*m*^2^. This also gives an estimate for the parameter *I*_0_ (the rate of encountering a single shelter by a single fish): 4 < *I*_0_ < 28 year^−1^.

We can now estimate the value of *r*_1_ (defined above). This value is given by the product between the number of metacercariae received by a fish in a single contest (Δ_*c*_ = 10 − 15 metacercariae) and the average number of the contests per year (estimated in the above paragraph), i.e. *r*_1_ = Δ_*c*_*I*_0_. We can evaluate *ν*_*μ*_ using the estimates for Δ_*S*_, based on the same assumption that the mortality is proportional to the parasite load P^*^. Since, in the absence of shelters, we have P^*^ = *r*/*α*, and this corresponds to empirically reported values 0.1 < Δ_*S*_ < 2.5 year^−1^ (see above estimates), adding an extra influx of parasites into the fish, modeled by *r*_1_, should be proportional to the increase of the corresponding mortality term. This reasoning gives an estimate

$${\nu }_{\mu }\approx {\Delta }_{S}\frac{{r}_{1}}{r}={\Delta }_{S}\frac{{\Delta }_{c}\cdot {I}_{0}}{r}.$$

Therefore, we have the range of 0.02 < *ν*_*μ*_/Δ_*S*_ < 0.2, and 0.002 < *ν*_*μ*_ < 0.5 year^−1^. We also allow for some smaller values for the lower bound for *ν*_*μ*_ to take into account the fact that a fish, which can potentially see a shelter (i.e. when passing within 1.5-2 *m* nearby), may miss that shelter or ignore it. Therefore, we consider a wider range 0.0005 < *ν*_*μ*_ < 0.5 year^−1^.

### Ethical note

We used 0+ Oncorhynchus mykiss, with equal number males and females. The level of experimental *D. pseudospathaceum* infection was maintained at a much lower level than the maximum values reported for naturally occurring infections (up to 200-500 individuals per fish), see^85,86^. The mortality of infected fish in the experiments was less than 1% and did not exceed that of the control fish. No visible damage was observed in any fish. We minimized the required number of animals that were killed and dissected. Experimental fish were killed at the end of the tests with an overdose of MS222 and dissected. In total, 240 experimentally infected fish were killed. The experiments were conducted with the permission of the National Animal Experiment Board, Center for Economic Development, Transport and Environment of South Finland (license number ESAVI/6759/04.10.03/2011). We have complied with all relevant ethical regulations for animal use.

### Statistics and reproducibility

The empirical data were analyzed using the Mann–Whitney U test (Exp. 1) and one-way ANOVA (Exp. 2). Prior to the analysis, the experimental data were checked for normality with Shapiro–Wilk’s W test. Tukey HSD test was applied for post-hoc pairwise comparisons (see details in Supplementary Notes 2 and 3). The sample sizes represent independent biological replicates. In experiments with individual fish, replicates were defined as the infection load of each individual (measured in the number of metacercariae); in experiments with a group of fish, replicates were defined as the mean infection load of a group. The number of replicates was 20 or 40 (when comparing the infection load of individually infected fast and slow fish). The sample size in the experiment comparing infection rate in fast and slow fish (Exp. 1) was 120 fish, in the experiment on the effect of anticipated threat (Exp. 2), the sample size was 160 fish. Other relevant information is provided in Fig. 6 legend as well as in Supplementary Notes 2 and 3.

### Reporting summary

Further information on research design is available in the Nature Portfolio Reporting Summary linked to this article.

### Supplementary information

Supplementary information

Reporting Summary

## Data Availability

Experimental data can be found in the file ‘Supplementary Information’, Supplementary Notes 2, 3.

## Code availability

The custom computer code can be found via 10.5281/zenodo.10376104.
