# Supplementary material for: Revisiting the role of behavior-mediated structuring in the survival of populations in hostile environments
Source: Commun Biol. 2024 Jan 12;7:93. doi: 10.1038/s42003-023-05731-z (PMC10786947; doi:10.1038/s42003-023-05731-z)
Supplement: Supplementary file 1 — Supplementary information [file 42003_2023_5731_MOESM1_ESM.pdf]

Supplementary Information: Revisiting the role of behavior-mediated structuring in the survival of populations in hostile environments

Simran Sandhu [1], Victor N. Mikheev [2], Anna F. Pasternak[3],
Jouni Taskinend[4], Andrew Morozov* [1,2]

[1]School of Computing and Mathematical Sciences, University of Leicester, Leicester LE1 7RH, UK

[2]Institute of Ecology and Evolution, Russian Academy of Sciences, Moscow, Russia

[3] Institute of Oceanology Russian Academy of Sciences, Russian Academy of Sciences, Moscow, Russia

[4] University of Jyväskylä, Jyväskylä, Finland

*To whom correspondence should be addressed; E-mail: am379@leicester.ac.uk

Contents

1 Supplementary Note 1. The life cycle of the eye fluke *Diplostomum pseudospathaceum*. 2

2 Supplementary Note 2. Experimental distribution of fish individuals according to their reaction time (slow-fast individuals). 3

3 Supplementary Note 3. Raw experimental data and statistical analysis related to interactions between rainbow trout *Oncorhynchus* and its parasite eye-fluke *D. pseudospathaceum*. 5

3.1 Figure 6A,B of the main text. Difference of infection loads in fast and slow fish . . . 5

3.2 Consistency in parasite acquisition in individual fish 7

3.3 Figure 6C of the main text. Variation of infection burden of fish under different anticipated threats. 11

4 Supplementary Note 4. Derivation of the Model for Monomorphic Population 12

5 Supplementary Note 5. Adaptive Dynamics Framework 13

6 Supplementary Note 6. Special Case: Dimorphic Population 14

7 Supplementary Note 7. Investigating the Stability of Fast System. 17

7.1 Monomorphic Population 17

7.2 Dimorphic Population 17

7.3 Polymorphic Population 17

8 Supplementary Note 8. Testing the robustness of the model to a change in parameters. 18

8.1 Monomorphic population: robustness of the initial drop in the population size as shelters are added 18

8.2 Monomorphic population: simulated examples of different parameters 19

8.3 Polymorphic population: simulated examples for different parameters 23

8.4 Impact of the parameter ϵ on the evolutionary dynamics 31

1 Supplementary Note 1. The life cycle of the eye fluke *Diplostomum pseudospathaceum.*

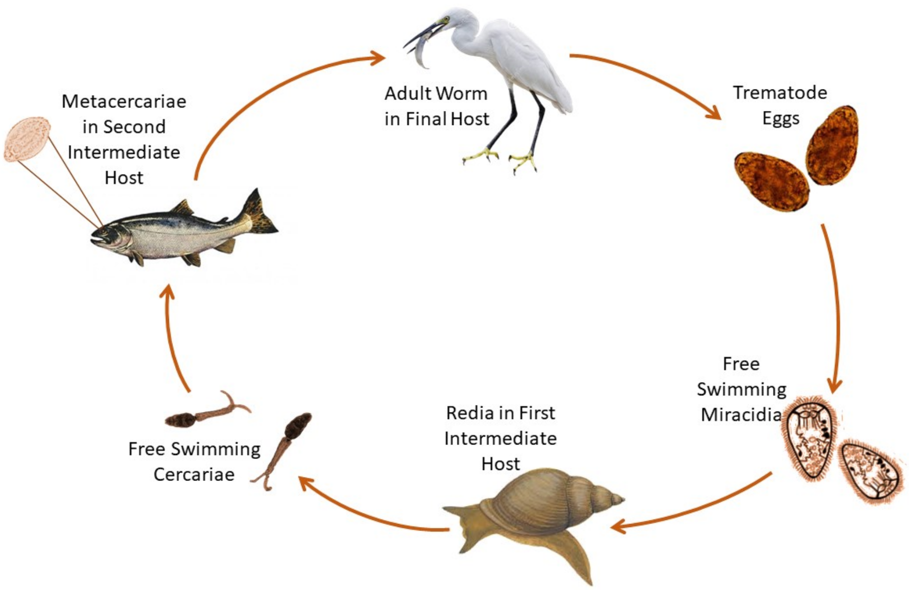

Figure S1: The life cycle of the eye fluke, *Diplostomum pseudospathaceum.* This common trematode lives in fish-eating birds (their final host) as mature sexually reproducing worms (marita). Eggs are evacuated with faeces in the water, where they develop into miracidia, free-swimming ciliated larvae which penetrates snails, the first intermediate host. There, miracidia asexually reproduce and the next larvae, cercariae, are released into the water. Next, cercariae penetrate their second intermediate host, fish, and develop there to metacercariae in the eye lenses of the host. Finally, trematodes develop into adult worms when the fish is eaten by a piscivorous bird.

2 Supplementary Note 2. Experimental distribution of fish individuals according to their reaction time (slow-fast individuals).

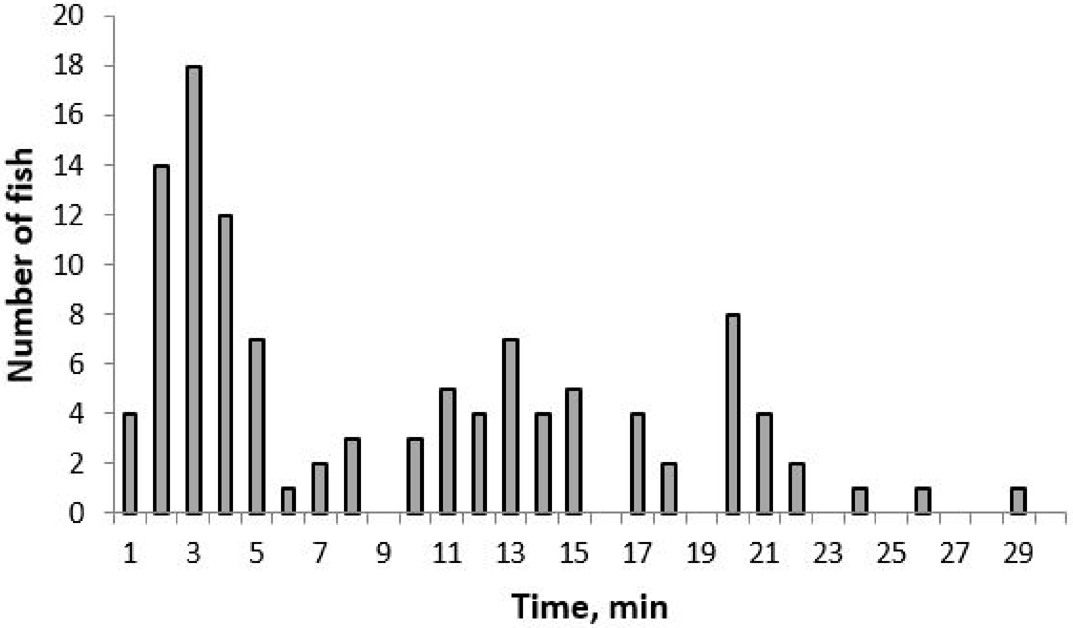

Figure S2: Experimental distribution of fish individuals according to their reaction time. The figure demonstrates a bimodal pattern in the distribution of the reaction time with a minimum of around 6 min. For more detail see the text below.

Prior to the experiment, approximately 400 fish were kept in a flow-through tank of 2.5 m^3 on 15:9 L : D cycle at 15-16 °C and fed with commercial pelleted food (1.5 mm size, Nutra Parr LB, Norway). Before scoring, fish were allowed to acclimate to the new housing conditions (filtered lake water with 2-3 degrees higher temperature than in the indoor tanks supplied with groundwater at the commercial fish farm) for 6-7 days. The food was the same as at the fish farm. After the scoring, the fish were kept individually in 10-l flow-through aquaria and fed daily with the same commercial pelleted food as before scoring.

In the behavioural scoring experiment, 120 fish were randomly chosen out of 400 and sorted according to their slow-fast reaction to a new environment. Fish were placed individually into a novel compartmentalised tank, and the time before fish moved from the initial compartment to the other one was recorded (reaction time). We used six dark brown two-compartment tanks (total length × width × height: 140 × 30 × 40 cm, volume 168 L). Each tank was separated into two compartments (70 × 30 × 40 cm each) by a divider with a rectangular hole of 5 × 3 cm near the bottom. This hole could be closed and opened by raising the door to control the passage of fish from one compartment to another. The water in the experimental tanks was filtered water pumped from the nearby lake at the environmental temperature (15-16°C). The oxygen saturation in the water was 90-94%.

Fish were considered as 'Fast' (more reactive) if it took them ≤ 5 min to move to the other compartment, and 'slow' (less reactive) if they stayed for a longer time. The resultant histogram of the reaction time is shown in Fig.S2. If the fish stayed longer than 30 min in the initial compartment, they were not used in subsequent experiments (such individuals were not included in the presented diagram in Fig.S2).

Fish were not fed for a day before each trial; the water was changed with tanks being thoroughly rinsed after each trial. There was a 10-min acclimation period in the scoring tank before opening the door. The scoring experiments took place within two consecutive days. After the scoring, the fish were kept individually in 10-l flow-through aquaria and fed daily with the same commercial pelleted food as before scoring.

40 fish were randomly chosen out of each group (fast and slow) and their Fork Length (FL) was compared. The results and statistical analysis using the Mann-Whitney test are presented below.

Table S1: Experimental distribution of fish individuals according to their reaction time (measured in minutes).

# of fish specimen	Fork length of Fast and Slow, cm	
	Fast - 1	Slow - 2
1	10.5	8.8
2	9.3	7.7
3	8.8	10.3
4	7.2	9.9
5	9.3	7.8
6	9	8.3
7	8.2	9.4
8	7.6	9.1
9	10.2	8.1
10	9.8	8.6
11	8	8.9
12	7.8	9.5
13	9.7	8.1
14	9	7.2
15	8.5	8.5
16	7.5	9.3
17	10.2	7.4
18	9	8.6
19	7.6	10.2
20	6.7	8.9
21	9.8	9.1
22	7.8	9
23	8.5	8.6
24	8.5	7.3
25	8.9	9.2
26	8.5	9
27	8.1	7.4
28	8.8	8.7
29	8.5	9.1
30	8.3	8.4
31	8.1	9.1
32	7.4	9.3
33	7.8	8.9
34	9.8	9.1
35	9.6	8.4
36	9.6	7.9
37	8.3	6.8
38	7.6	8.5
39	9.4	7.9
40	9.3	8.3
Mean	8.6625	8.615
SD	0.923396	0.797287

Mann-Whitney U Test
By variable Var2
Marked tests are significant at p <0.05000

Rank Sum Group 1	Rank Sum Group 2	U	Z	p-value	Z adjusted	p-value	Valid N Group 1	Valid N Group 2	2*1sided exact p
1630.000	1610.000	790.0000	0.091414	0.927164	0.091513	0.927085	40	40	0.927574

3 Supplementary Note 3. Raw experimental data and statistical analysis related to interactions between rainbow trout *Oncorhynchus* and its parasite eye-fluke *D. pseudospathaceum.*

In this supplementary material, we provide the raw experimental data regarding interactions between rainbow trout *Oncorhynchus* and its parasite eye-fluke *Diplostomum pseudospathaceum.* We also provide details on the statistical analysis of the data conducted in Figures 6A,B,C. Data analysis was performed using EXCEL and Statistica software.

3.1 Figure 6A,B of the main text. Difference of infection loads in fast and slow fish

Figure 6A. Infection load of isolated fish (fast and slow). Raw data and statistics are presented below. Details on the experimental method used are provided in the main text.

Table S2: Experimental infection load, in fast and slow fish (isolated fish), measured in number metacercariae per fish at the end of experiment.

# of fish specimen	Infection load, number metacercariae per fish at the end of experiment	
	Fast	Slow
1	36	49
2	39	72
3	50	51
4	55	60
5	45	52
6	26	76
7	32	44
8	70	45
9	77	66
10	45	61
11	38	43
12	44	62
13	72	59
14	46	83
15	91	72
16	69	38
17	32	39
18	18	58
19	83	72
20	26	72
21	60	36
22	30	57
23	45	86
24	50	74
25	60	54
26	36	32
27	45	40
28	73	36
29	41	46
30	34	50
31	21	49
32	48	80
33	49	118
34	66	46
35	32	50
36	55	72
37	72	66
38	44	58
39	55	72
40	62	72
Mean	49,3	59,2
SD	17,53268	17.20495

Mann-Whitney U Test
By variable "reactivity"
Marked tests are significant at $p < 0.05000$

Rank Sum Bold	Rank Sum Shy	U	Z	p-value	Z adjusted	p-value	Valid N Bold	Valid N Shy
1361.50	1878.50	541.500	-2.48261	0.01304	-2.48552	0.01293	40	40

Figure 6B. Group infection of fish (2 fast and 2 slow fish in a compartmentalised tank). Raw data and statistical analysis are presented below. Infection rate (number of metacercariae per fish) in fast and slow fish.

Table S3: Experimental infection load, in fast and slow fish (group experiment), measured in number metacercariae per fish at the end of experiment.

# of fish specimen	Infection load, number metacercariae per fish at the end of experiment	
	Fast	Slow
1	22	54
2	7	24
3	11	146
4	4	48
5	53	53
6	51	140
7	71	108
8	38	56
9	58	49
10	58	55
11	19	24
12	21	27
13	26	22
14	16	40
15	34	66
16	38	101
17	28	26
18	49	51
19	2	46
20	67	93
Mean	33.65	61.45
SD	21.3474	37.1433

Mann-Whitney U Test
By variable "reactivity"
Marked tests are significant at $p < 0.05000$

Rank Sum Fast	Rank Sum Slow	U	Z	p-value	Z adjusted	p-value	Valid N Fast	Valid N Slow
296.500	333.500	86.5000	-2.10000	0.03573	-2.10088	0.03565	20	20

3.2 Consistency in parasite acquisition in individual fish

Here we provide details on the consistency in parasite acquisition in individual fish in the first and the second exposure to parasites.

The outcome (intensity of parasitism) of the first and the second rounds of infection of each particular individual fish used in the experiments (Fig. S3). This result demonstrates an overall consistency of the vulnerability of individuals to parasite infection: there is a positive correlation between the number of parasites received in the first and second infection of the experiment. The figure also shows consistency in parasite acquisition by an individual fish.

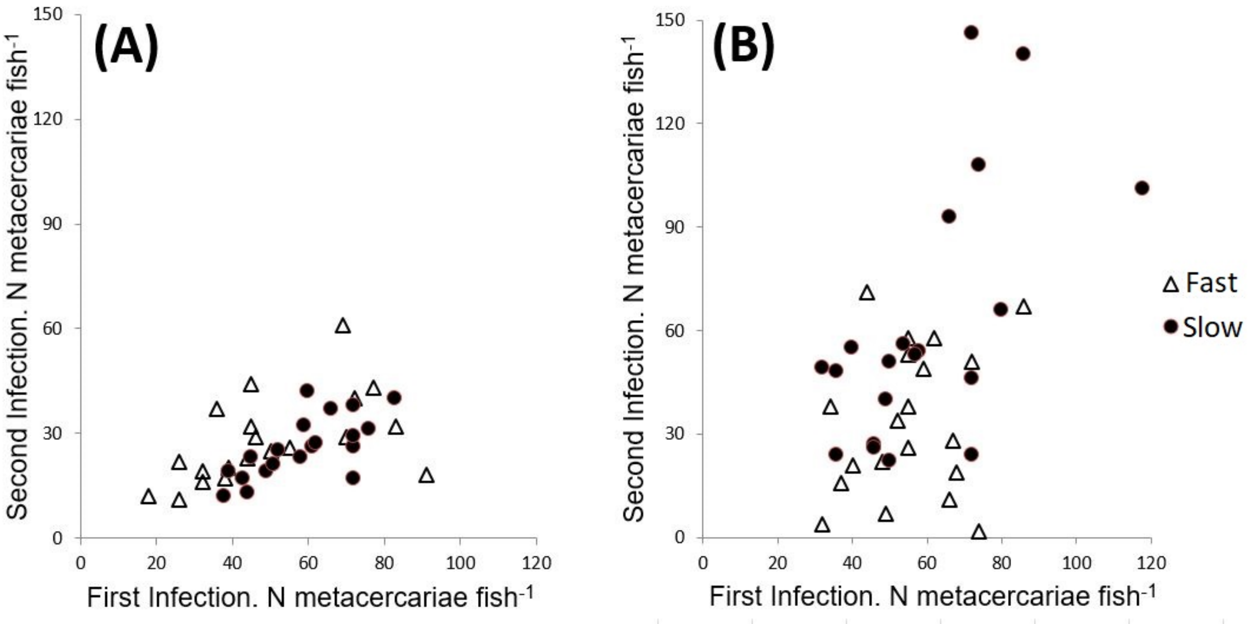

Figure S3: Consistency in parasite acquisition. Effects of reactivity, grouping and parasite distribution. (A) When fish were infected individually (first infection - all fish), the second infection (half of the fish infected individually, half - in groups of 4) resulted in a lower parasite load than the first one in both fast and slow fish. (B) When fish were infected in groups in a heterogeneous (structured) habitat, the second infection resulted in increased variability in parasite load. In the ‘structured’ habitat, parasites were distributed unevenly (one compartment containing parasites, another free of them), but the average concentration in the whole tank was the same as in individual infections. A total of 80 fish were tested with triangles and filled circles representing fast and slow fish, respectively. Details on statistical analysis are provided in the supplementary material.

Raw data and statistical analysis are presented below emphasising the role of reactivity, grouping and parasite distribution. The infection load is measured by the number of metacercariae per fish.

Table S4: Experimental infection load for fast and slow fish (for group and isolated fish), after the 1st and the 2nd infection events.

	Number	1st inf, individual	2nd inf, individual	1st inf, individual	2nd inf, group
Fast	1	36	37	48	22
	2	39	20	49	7
	3	50	25	66	11
	4	55	26	32	4
	5	45	32	55	53
	6	26	22	72	51
	7	32	16	44	71
	8	70	29	55	38
	9	77	43	62	58
	10	45	44	55	58
	11	38	17	68	19
	12	44	23	40	21
	13	72	40	55	26
	14	46	29	37	16
	15	91	18	52	34
	16	69	61	34	38
	17	32	19	67	28
	18	18	12	59	49
	19	83	32	74	2
	20	26	11	86	67
Slow	1	49	19	58	54
	2	72	38	72	24
	3	51	21	72	146
	4	60	42	36	48
	5	52	25	57	53
	6	76	31	86	140
	7	44	13	74	108
	8	45	23	54	56
	9	66	37	32	49
	10	61	26	40	55
	11	43	17	36	24
	12	62	27	46	27
	13	59	32	50	22
	14	83	40	49	40
	15	72	26	80	66
	16	38	12	118	101
	17	39	19	46	26
	18	58	23	50	51
	19	72	29	72	46
	20	72	17	66	93

Correlation between 1st and 2nd individual infection
Fast and Slow combined
Spearman Rank Order Correlations (Spreadsheet1)
MD pairwise deleted
Marked correlations are significant at p <,05000

Valid	Spearman	t(N-2)	p-value
N	R		
35	0.616221	4.494720	0.000081

Correlation between 1st individual and 2nd group infection
Fast and Slow combined
Spearman Rank Order Correlations (Spreadsheet1)
MD pairwise deleted
Marked correlations are significant at p <,05000

Valid	Spearman	t(N-2)	p-value
N	R		
35	0.358847	2.208513	0.034264

Correlation between 1st and 2nd individual infection
Fast
Spearman Rank Order Correlations (Spreadsheet1)
MD pairwise deleted
Marked correlations are significant at p <,05000

Valid	Spearman	t(N-2)	p-value
N	R		
20	0.595480	3.144764	0.005602

Correlation between 1st and 2nd individual infection
Slow
Spearman Rank Order Correlations (Spreadsheet1)
MD pairwise deleted
Marked correlations are significant at p <,05000

Valid	Spearman	t(N-2)	p-value
N	R		
20	0.713532	4.320810	0.000412

Correlation between 1st individual and 2nd group infection
Fast
Spearman Rank Order Correlations (Spreadsheet1)
MD pairwise deleted
Marked correlations are significant at p <,05000

Valid	Spearman	t(N-2)	p-value
N	R		
20	0.177493	0.765190	0.454075

Correlation between 1st individual and 2nd group infection
Slow
Spearman Rank Order Correlations (Spreadsheet1)
MD pairwise deleted
Marked correlations are significant at p <,05000

Valid	Spearman	t(N-2)	p-value
N	R		
20	0.602943	3.206467	0.004892

3.3 Figure 6C of the main text. Variation of infection burden of fish under different anticipated threats.

To assess the vulnerability of fish to *D. pseudospathaceum*, we compared infection rates in fish demonstrating either territorial or grouping behaviour with that of a solitary fish deprived of shelters and conspecifics. Solitary fish were tested over either the dark or light bottom. The white background is assumed to be more stressful than the dark one over which fish are cryptic.

Four experimental situations with different levels of anticipated threat were compared: (a) the least dangerous situation (the least infection) for an individual fish was related to a cover shelter in a light bottom tank; (b) more stressed (more infected) were fish in a group (shoal) in the light tank; (c) almost the same level of infection as in (b) was in individual fish over the dark bottom - young salmonid fish are cryptic over the dark substrate; (d) position of an individual fish over the light bottom was the most stressful (highest infection rate).

All the tests on individual (solitary) fish were performed in light plastic tanks of 10 l of filtered lake water. Groups of fish were tested in 50 l light plastic tanks. As a cover shelter, we used a dark rectangular plate of 15×15 cm mounted on four small stones. For experiment (c), the bottom was covered with dark plastic. Fish were exposed to 60 ind l^{-1} *D. pseudospathaceum* cercariae for 15 min after 30 min of acclimation. Twenty replicates of each of the experimental trials were performed with new fish used for each trial.

The infection load (measured through the number of metacercariae per fish) of solitary and grouping fish at different levels of the anticipated threat are shown in the table below. Description of statistical method is provided after the table.

Table S5: Experimental infection load (measured through the number of metacercariae per fish) of solitary and grouping fish at different levels of the anticipated threat.

# of fish	Sheltering	Grouping	Cryptic habitat	Dangerous habitat
1	30	51	36	51
2	25	34	32	32
3	28	27	49	30
4	35	34	53	64
5	26	44	51	45
6	22	44	30	53
7	26	31	28	81
8	25	24	32	49
9	11	54	55	34
10	18	10	23	61
11	15	34	36	51
12	22	65	32	32
13	15	24	49	30
14	15	27	53	64
15	29	24	51	45
16	28	20	30	53
17	18	27	28	81
18	8	82	32	49
19	21	34	55	34
20	31	48	23	61
Mean	22.40	36.89	38.80	49.82
SD	7.18	16.776	11.55	15.54

The experimental data were checked for normality with the Shapiro-Wilk's W test. We found that the data met the criteria.

SS	Degr. of Freedom	MS	F	p
109380,	1	109380.	618.044	0.00000
7615,0	3	2538.3	14.3425	0.00000
13450.4	76	177.0		

Tukey HSD test

Approximate Probabilities for Post Hoc Tests

Error: Between MS = 176,98, df = 76,000

		{1}	{2}	{3}	{4}
		49.820	38.796	36.890	22.400
1	Dangerous hab.		0.05087	0.01533	0.00014
2	Cryptic	0.0508		0.96889	0.00127
3	Shoaling	0.0153	0.96889		0.00516
4	Shelter	0.0001	0.00127	0.00516	

4 Supplementary Note 4. Derivation of the Model for Monomorphic Population

Here we show how one can directly derive the equation for the population dynamics in the case of a monomorphic population, i.e. under the scenario without structuring individuals in terms of their boldness. We denote by T and S the number of fish using the territorial and shoaling behavioral tactics, respectively. The total population is given by $F = T + S$. We assume that the shelters are wholly occupied, i.e. a vacant shelter is immediately invaded by an individual from the shoal, i.e. $T = N$ (for $F > N$). We consider that exchange between the compartments T and S occurs on a faster time scale compared to the slow demographic process, meaning a particular individual can change its tactics several times. In this case, $T = N$ and $S = F - T$ are understood as the number of individuals who, on average, use territorial or shoaling tactics, respectively. The equation for the population dynamics for the total number of fish F on the slow demographic scale is given by

$$\frac{dF}{dt} = b(F)F - (m_0F + \Delta m_S S + \Delta m_T T) - G(S, N). \tag{S1}$$

In the above equation, $b(F)$ is the per capita reproduction rate which is the same for each individual (we assume it to be linear $b = b_0(1 - F/K)$, where K is the carrying capacity, b_0 is the maximal growth rate); m_0 is the background mortality through natural causes; Δm_S is the increase in mortality due to predation/parasites in the case an individual use the shoaling tactics; Δm_T is the increase in mortality due to predation/parasites in the case an individual uses the territorial tactics. The function $G(S, N)$ describes the extra mortality due to fighting for the shelters between individuals from the shoal and the current occupiers of the shelters.

We further substitute $S = F - N$ and $T = N$. The function $G(S, N)$ is constructed by assuming that an increase in mortality is proportional to the number of contests for shelters, which, in turn, is proportional to the number of occupied shelters and the number of animals in the shoal (S) which sporadically try to contest these shelters.

$$G(S, N) = (F - N)Nm_p(1 + D)\nu\omega, \tag{S2}$$

where m_p is the parameter characterizing the intensity of the extra mortality as the result of fighting; ν describes the role of boldness in efficiency of the search; ω gives the conditional probability that the invader and the occupier would start contest of the shelter (to be consistent with the polymorphic model we set $\omega = 0.5$) The multiplier $(1 + D)$, accounts for the fact that the fighting-induced mortality of the shelter's

occupier is reduced by the factor D, compared to that of the invader. This parameter varies from 0 (the occupier of the shelter does not suffer extra mortality due to the fight) to 1 (the occupier of the shelter exerts identical extra mortality to that of the invader). Note that this assumption is based on some previous empirical observations. The equation for the population dynamics for F on a slow scale becomes

$$\frac{dF}{dt} = b(F)F - (m_0 F + \Delta m_S(F-N) + \Delta m_T N) - m_p\nu(F-N)N(1+D)/2. \tag{S3}$$

Equation (S3) is valid under the condition that $F > N$. The parameters $\Delta m_S, \Delta m_T, m_p, \nu$ may depend on boldness.

5 Supplementary Note 5. Adaptive Dynamics Framework

In the main paper, we also studied the evolution of boldness using the adaptive dynamics framework (Geritz, Metz, Kisdi, & Meszéna, 1997; Brännström, Johansson, & Von Festenberg, 2013), which considers the long-term evolutionary outcome of the invasion of a rare mutant with boldness B_m into the environment formed by a resident (with boldness B_r) at its ecological equilibrium, F_r^*. The outcome is characterized by invasion fitness (defined as the long-term average growth rate of a rare invading mutant), where positive fitness indicates a successful invasion, with the mutant displacing the resident. This process occurs iteratively, with successive mutant invasions which, when successful, exclude the resident (Eshel, 1983; Taylor, 1989; Christiansen, 1991; Abrams, Matsuda, & Harada, 1993). Following a large number of such invasions and substitutions, the species will evolve until an evolutionary singular point is reached, at which the selection gradient (defined as the derivative of the invasion fitness) vanishes. The analytical expression for the invasion fitness is given in SM4.

Pairwise Invasibility Plots (Kisdi & Meszéna, 1993) (PIPs) are graphical illustrations of the invasion success of potential mutants, displaying all the mutant traits for which the invasion fitness is positive, i.e. a successful invasion, for each resident. These PIPs suggest the subsequent evolutionary behavior of an evolutionary singularity (Taylor, 1989; Abrams et al., 1993). The singularities can either be stable (an evolutionary attractor), unstable (an evolutionary repellor) or a branching point. An evolutionary attractor occurs when a singular point is both a convergent and evolutionary stable strategy (ESS). These joint conditions ensure that an ESS can be attained and maintained (Eshel, 1983). If the singularity is convergent stable but not an ESS then it is a branching point, meaning an initially monomorphic population becomes dimorphic (Doebeli, 2011; Dieckmann & Doebeli, 1999).

Here we derive the analytical expression for invasion fitness. We apply the adaptive dynamics framework and consider the invasion of a rare mutant strain of boldness B_m (for the population density, we have $F_m = T_m + S_m$). The boldness of the resident is denoted by B_r and the population density of the resident at equilibrium is given by $F_r^* = T_r^* + S_r^* = N + S_r^*$. The equation for fast transitions for the mutant is as follows

$$M_m = \left(S_m\nu(B_m)\omega(B_m,B_r)N - S_r^* T_m\nu(B_r)\omega(B_r,B_m)\right) I_0. \tag{S4}$$

Using $F_m = T_m + S_m$, the above equation ($M_m = 0$) can be solved as

$$T_m = \frac{F_m N\nu(B_m)\omega(B_m,B_r)}{(F_r^* - N)\nu(B_r)\omega(B_r,B_m) + N\nu(B_m)\omega(B_m,B_r)}, \tag{S5}$$

which we use in the slow demographic model;

$$\begin{aligned}\frac{dF_m}{dt} = b(F_r^*)F_m - m_0F_m - \Delta m_S(B_m)(F_m - T_m) - \Delta m_T(B_m)T_m \\ - m_p(B_m)\nu(B_m)(F_m - T_m)\omega(B_m,B_r)N(1+D),\end{aligned} \tag{S6}$$

where $m_p(B_m) = (1-\epsilon B_m)\nu_\mu$.

From the above we can derive the invasion fitness as ($dF_m/dt = \lambda_m F_m$)

$$\lambda_m = b(F_r^*) - m_0 - \Delta m_S(B_m) + (\Delta m_S(B_m) - \Delta m_T(B_m))\frac{T_m}{F_m} - m_p(B_m)\nu(B_m)\omega(B_r,B_m)(1+D)N(1-\frac{T_m}{F_m}). \tag{S7}$$

Here the ratio T_m/F_m can be found from (S5). The stationary population density of the resident F_r^* can be found by solving the following quadratic equation which comes from (S3) when only a single strain B_r is present

$$\begin{aligned}\frac{-b_0}{K}(F_r^*)^2 + (b_0 - m_0 - \Delta m_S(B_r) - (1+D)m_p(B_r)\nu(B_r)N/2)\,F_r^* \\ +(\Delta m_S(B_r) - \Delta m_T(B_r))N + (1+D)m_p(B_r)\nu(B_r)N^2/2 = 0.\end{aligned} \tag{S8}$$

6 Supplementary Note 6. Special Case: Dimorphic Population

Here we analytically explore the stationary states of the model from the main text in the case where only two boldness strains are present, denoted by B_1 and B_2 ($B_2 > B_1$). The instantaneous distribution of strains between the shoal and shelters is found by solving the following fast dynamics equations for M_i. As before, we assume that all shelters are filled.

$$M_i = S_i I(B_i, B_j, T_j) - S_j I(B_j, B_i, T_i) = 0, \quad i = 1, 2. \tag{S9}$$

As $M_1 = -M_2$ we can just solve $M \equiv M_1 = 0$;

$$M = S_1 I(B_1, B_2, T_2) - S_2 I(B_2, B_1, T_1) = 0. \tag{S10}$$

As $T_1 + T_2 = N$ and $F_i = S_i + T_i$, $i = 1, 2$, are fixed on the fast time scale, we have

$$(F_1 - T_1)I(B_1, B_2, N - T_1) - (F_2 - N + T_1)I(B_2, B_1, T_1) = 0. \tag{S11}$$

The rate of invasion of shelters is parameterized as $I(B_i, B_j, T_j) = 2\nu(B_i)\omega(B_i, B_j)T_j I_0$. In the case the difference in boldness is large, we can approximate $\omega(B_i, B_j)$ by a step-wise function, such that a shy strain never attempts to invade a bolder strain (i.e. if $B_1 < B_2$ then $I(B_1, B_2, T_2) = 0$). Thus

$$M = -(F_2 - N + T_1)I(B_2, B_1, T_1) = 0, \tag{S12}$$

which is only satisfied when $T_1 = 0$, and, therefore $T_2 = N$. The bolder strain fully occupies the shelters, however, note that this strain is still present in the shoal. Giving the following model equations;

$$\frac{dF_1}{dt} = b(F_1 + F_2)R(B_1, \mathbf{F}) - (m_0 F_1 + \Delta m_S(B_1)F_1)\,, \tag{S13}$$

$$\begin{aligned}\frac{dF_2}{dt} = b(F_1 + F_2)R(B_2, \mathbf{F}) - (m_0 F_2 + (\Delta m_S(B_2)(F_2 - N) + \Delta m_T(B_2)N)) \\ -m_p(B_2)\nu(B_2)(F_2 - N)N\,(1 + D)\,.\end{aligned} \tag{S14}$$

Below we will consider the two following cases: (i) clonal reproduction and (ii) uniform genetic mixing.

(i) Clonal reproduction; $R(B_i, F_i) = F_i$. The stationary states are determined by following system ($F_2 > N$)

$$0 = \frac{dF_1}{dt} = b(F_1^* + F_2^*)F_1^* - (m + \Delta m_S(B_1))\,F_1^*, \tag{S15}$$

$$\begin{aligned}0 = \frac{dF_2}{dt} = b(F_1^* + F_2^*)F_2^* - (m_0 F_2^* + (\Delta m_S(B_2)(F_2^* - N) + \Delta m_T(B_2)N)) \\ -m_p(B_2)\nu(B_2)(F_2^* - N)N\,(1 + D)\,.\end{aligned} \tag{S16}$$

From equation (S15) we can derive the following;

$$0 = b(F_1^* + F_2^*) - (m_0 + \Delta m_S(B_1))\,. \tag{S17}$$

As $b(F) = b_0(1 - \frac{F}{K})$, we can conclude the following;

$$F^* = F_1^* + F_2^* = \frac{K}{b_0}(b_0 - (m_0 + \Delta m_S(B_1))). \tag{S18}$$

One can see that the stationary total number of fish at equilibrium F^* is independent of N.

Note that the population density depending only on $\Delta m_s(B_1)$) does not necessarily mean that there are no bold fish in the shoal. Indeed, let us assume $F_2^* = N$ i.e. there are no bold fish in the shoal. Then

$$0 = b(F_1^* + N)N - (m_0 N + \Delta m_T(B_2)N))\,. \tag{S19}$$

As $0 = b(F_1^* + F_2^*) - (m_0 + \Delta m_S(B_1))$ we can simplify this to

$$0 = (m_0 + \Delta m_S(B_1))N - (m_0 N + \Delta m_T(B_2)N)) = \Delta m_S(B_1)N - \Delta m_T(B_2)N. \tag{S20}$$

This directly implies that $m_S(B_1) = m_T(B_2)$, which is not always true.

(ii) Uniform reproduction; $R(B_i, F_i) = \frac{F_1+F_2}{2}$. For the stationary states we have the following system;

$$0 = \frac{dF_1}{dt} = b(F_1^* + F_2^*)\frac{F_1^* + F_2^*}{2} - (m_0 + \Delta m_S(B_1))\,F_1^*, \tag{S21}$$

$$\begin{aligned} 0 = \frac{dF_2}{dt} = b(F_1^* + F_2^*)\frac{F_1^* + F_2^*}{2} - (m_0 F_2^* + (\Delta m_S(B_2)(F_2^* - N) + \Delta m_T(B_2)N)) \\ - m_p(B_2)\nu(B_2)(F_2^* - N)N\,(1 + D)\,. \end{aligned} \tag{S22}$$

From equation (S21) we can derive the following;

$$F_2^* = \frac{K}{2}\left(1 \pm \sqrt{1 - \frac{8}{b_0 K}(m_0 + \Delta m_S(B_1))F_1^*}\right) - F_1^*. \tag{S23}$$

Combining (S21) and (S22) we obtain

$$0 = \Delta m_S(B_1)F_1^* - (m(F_2^* - F_1^*) + \Delta m_S(B_2)(F_2^* - N) + \Delta m_T(B_2)N) - m_p(B_2)\nu(B_2)(F_2^* - N)N\,(1 + D)\,. \tag{S24}$$

From which the following can be derived

$$F_2^* = \frac{(\Delta m_S(B_1) + m_0)F_1^* + (\Delta m_S(B_2) - \Delta m_T(B_2) + m_p(B_2)\nu(B_2)N\,(1 + D))N}{m_0 + \Delta m_S(B_2) + m_p(B_2)\nu(B_2)N(1 + D)}. \tag{S25}$$

The stationary values of F_i^* $(i = 1, 2)$ can be found by solving the above system of equations given by (S25) and (S23), which can be reduced to a quadratic equation. Some solutions to system (S25) and (S23) are shown graphically in Fig. S4. On the other hand, from (S25) one can see that a gradual increase the number of shelters N will eventually increase the value of F_2^* up to the level, where $F_2^* < N$. Note that for $F_2^* < N$, model equations (S13)-(S14) should be modified to include the possibility to have some proportion of the shy strain in shelters (for brevity, we do not show here the corresponding modified equations). We find the critical value N^* such that $F_2^* = N^*$, we use equations (S25) and (S15). In particular, based on (S25) we have the following expression for F_1^*;

$$F_1^* = \frac{m_0 + \Delta m_T(B_2)}{m_0 + \Delta m_S(B_1)}N^*. \tag{S26}$$

Therefore

$$F_1^* + F_2^* = F_1^* + N^* = \frac{2m_0 + \Delta m_S(B_1) + \Delta m_T(B_2)}{m_0 + \Delta m_S(B_1)}N^*. \tag{S27}$$

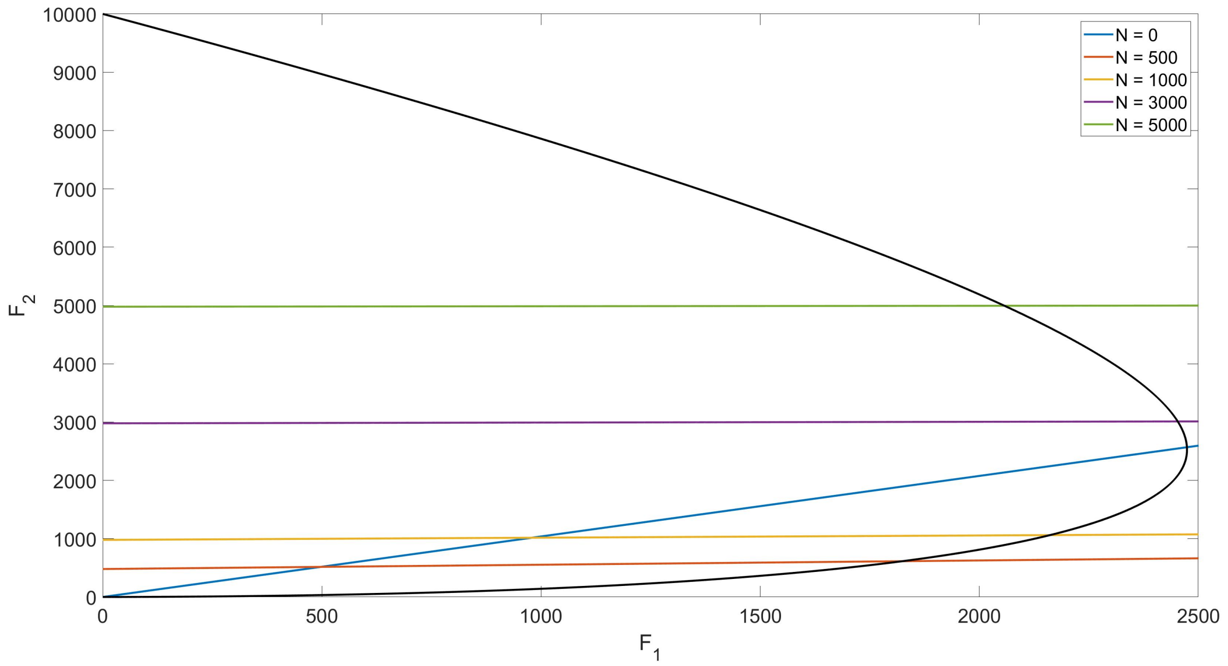

Figure S4: Locating stationary states from the intersection of analytically derived expressions (S23) and (S25) for varying values of shelters N. Two strains of individuals F_1 and F_2 were considered with boldness B_1 and B_2, respectively, where $B_2 > B_1$. The black curve describes equation (S23), which is independent of N, the remaining curves are defined by equation (S25). All other parameters are as given in Table 1 from the main text.

Substituting this into equation (S21) we obtain the following;

$$\frac{b_0}{2}\left(1-\frac{F^*(N^*)}{K}\right)\frac{2m_0+\Delta m_S(B_1)+\Delta m_T(B_2)}{m_0+\Delta m_S(B_1)}N-(m_0+\Delta m_T(B_2))N=0, \tag{S28}$$

which can be solved for F^*

$$F^*(N^*)=K\left(1-2\frac{(m_0+\Delta m_T(B_2))(m_0+\Delta m_S(B_1))}{b_0(2m_0+\Delta m_S(B_1)+\Delta m_T(B_2))}\right). \tag{S29}$$

Next, we compare the difference in the equilibrium population density in the absence of shelters ($N = 0$) and when their number becomes large such that $F_2^* = N$. For $N = 0$, the expression for the stationary density is a lengthy equation, so for simplicity, we assume that $\Delta m_S(B_1) \approx \Delta m_S(B_2)$ however, this assumption is not essential. In this case, the expression for $F^*(0)$ simplifies to

$$F^*(0)=K\left(1-\frac{(m_0+\Delta m_S(B_1))}{b_0}\right). \tag{S30}$$

In order to determine if sufficient sheltering can be beneficial to the system, opposed to no sheltering, we can consider the difference $F^*(N)-F^*(0)$. We obtain

$$F^*(N)-F^*(0)=\frac{K(m_0+\Delta m_S(B_1))}{b_0(2m_0+\Delta m_S(B_1)+\Delta m_T(B_2))}\left(\Delta m_S(B_1)-\Delta m_T(B_2)\right). \tag{S31}$$

It can be easily seen that provided $\Delta m_S(B_1) = \Delta m_S(B_2) > \Delta m_T(B_2)$ (the mortality rate for staying in shelters is smaller than that of staying in the shoal), then $F^*(N) - F^*(0) > 0$. This means that for uniform genetic mixing, adding a large number of shelters will be more beneficial than no sheltering at all.

7 Supplementary Note 7. Investigating the Stability of Fast System.

7.1 Monomorphic Population

Due to the underlying assumption of the model that all shelters are fully occupied at any given time we have the simplified monomorphic model;

$$\frac{dF}{dt} = b(F)F - (m_0F + \Delta m_S(F-N) + \Delta m_T N) - m_p(F-N)N(1+D)\nu/2. \tag{S32}$$

This underlying assumption tells us that $T = N$ for any given time therefore defining a state stationary state of the fast actions.

7.2 Dimorphic Population

In Appendix C of the main text we show that for two strains (B_1, B_2) the fast actions will reach the stationary states of $(T_1, T_2) = (0, N)$. Here, we show that these stationary states, of the fast actions, will always be stable.

$$M_i = (F_i - T_i)I(B_i, B_j, T_j) - (F_j - T_j)I(B_j, B_i, T_i) = 0, \quad i = 1, 2. \tag{S33}$$

which was simplified (since $M_1 = M_2 \equiv M$) to

$$M = (F_1 - T_1)I(B_1, B_2, N - T_1) - (F_2 - N + T_1)I(B_2, B_1, T_1) = 0. \tag{S34}$$

or which was simplified to

$$M = ((F_1 - T_1)\nu(B_1)\omega(B_1, B_2)(N - T_1) - (F_2 - N + T_1)\nu(B_2)\omega(B_2, B_1)T_1)I_0 = 0. \tag{S35}$$

From which we can derive the following;

$$\frac{\delta M}{\delta T_1} = -T_1\nu(B_1)\omega(B_1,B_2)(N-T_1)I_0 - (F_1-T_1)\nu(B_1)\omega(B_1,B_2)I_0 - \nu(B_2)\omega(B_2,B_1)T_1 - (F_2-N+T_1)\nu(B_2)\omega(B_2,B_1)I_0. \tag{S36}$$

At the stationary state $(T_1, T_2) = (0, N)$ can be simplified to the following;

$$\frac{\delta M}{\delta T_1} = -F_1\nu(B_1)\omega(B_1, B_2)I_0 - (F_2 - N)\nu(B_2)\omega(B_2, B_1)I_0. \tag{S37}$$

As $F_2 - N = F_2 - T_2 = S_2 > 0$ we have that $\frac{\delta M}{\delta T_1} < 0$ at $(T_1, T_2) = (0, N)$ and therefore the stationary state of the fast actions is stable.

7.3 Polymorphic Population

For the case of the generic polymorphic population the stationary state of the fast actions are described by

$$\begin{aligned} M_i = 0 = (F_i - T_i(F_i)) \left(\sum_{j=1}^{n-1} I(B_i, B_j, T_j(F_j)) + I(B_i, B_n, N - \sum_{j=1}^{n-1} T_j(F_j)) \right) \\ - \sum_{j=1}^{n-1} (F_j - T_j(F_j))I(B_j, B_i, T_i(F_i)) - (F_n - (N - \sum_{j=1}^{n-1} T_j(F_j)))I(B_n, B_i, T_i(F_i)) \end{aligned} \tag{S38}$$

for $i = 1 : n-1$ when $T_n(F_n) = N - \sum_{j=1}^{n-1} T_j(F_j)$. From the above equation we are able to numerically determine the Jacobian and corresponding eigenvalues for a polymorphic population model. Below we present an example of the dynamics of the eigenvalue having the maximal real part computed at the stationary state of the fast system. The maximal real part of the eigenvalue is always negative, thus the fast system is stable.

The figure shows that for any given slow demographic time, the stationary distribution of the sheltered individuals (as governed by the fast actions) is always stable.

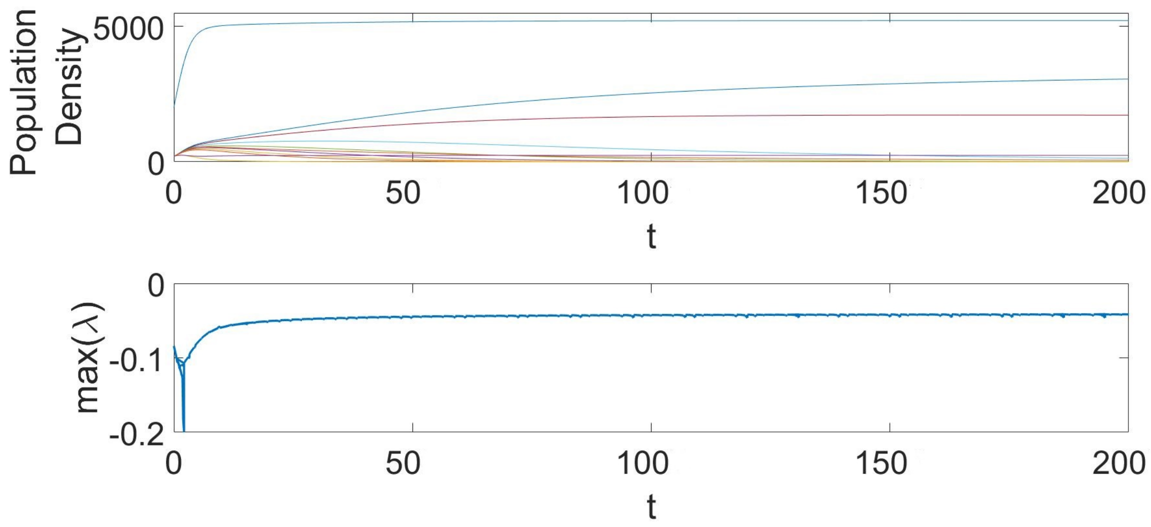

Figure S5: Stability of polymorphic fast transitional actions, time t is measured in years. The upper panel displays the simulated demographic actions for each strain of boldness with the corresponding maximal eigenvalue (the real part of the eigenvalue) of the stationary state of the fast transitions for each given time displayed in the lower panel. Here $N = 200$, we use $n = 10$ equally spaced values of boldness, and all other parameters are as given in Table 1 in the main text.

8 Supplementary Note 8. Testing the robustness of the model to a change in parameters.

8.1 Monomorphic population: robustness of the initial drop in the population size as shelters are added

We can determine the stationary state of the monomorphic population by solving the following;

$$b(F)F - (m_0F + \Delta m_S(B)(F - N) + \Delta m_T(B)N) - m_p(B)\nu(B)(F - N)N(1 + D)/2 = 0. \tag{S39}$$

Without loss of generality, let us fix the value of boldness B and therefore for brevity we can drop the notation of B to give the following;

$$b(F)F - (m_0F + \Delta m_S(F - N) + \Delta m_TN) - m_p(F - N)N(1 + D)\nu(B)/2 = 0. \tag{S40}$$

As $b(\sum_{j=1}^{n} F_j) = b_0\left(1 - \frac{\sum_{j=1}^{n} F_j}{K}\right)$ we can simply this to

$$\frac{b_0F^2}{K} - (b_0 - m_0 - \Delta m_S - m_p\nu N(1 + D))F/2 + \Delta m_TN - \Delta m_SN - m_p\nu N^2(1 + D)/2 = 0. \tag{S41}$$

Note that when $N = 0$

$$F = K\frac{b_0 - m_0 - \Delta m_S}{b_0}. \tag{S42}$$

We can then take the derivative with respect to N

$$\frac{b_0(2FF')}{K} - (b_0 - m_0 - \Delta m_S - m_pN(1+D))F'\nu/2 + m_p(1+D)F\nu/2 + \Delta m_T - \Delta m_S - 2m_pN(1+D)\nu/2 = 0. \tag{S43}$$

Now let us consider $N = 0$

$$\frac{b_0(2\left(K\frac{b_0-m_0-\Delta m_S}{b_0}\right)F')}{K} - (b_0 - m_0 - \Delta m_S)F' + m_p(1+D)\left(K\frac{b_0 - m_0 - \Delta m_S}{b_0}\right)\nu/2 + \Delta m_T - \Delta m_S = 0. \tag{S44}$$

We can solve for F':

$$F' = \frac{\Delta m_S - \Delta m_T - m_p(1+D)\left(K\frac{b_0-m_0-\Delta m_S}{b_0}\right)\nu/2}{b_0 - m_0 - \Delta m_S}, \tag{S45}$$

as $b_0 \gg m$ and $b_0 \gg \Delta m_S$ for most realistic parameter choices, let us assume that $b_0 - m_0 - \Delta m_S > 0$. Then

$$F' = \frac{\Delta m_S - \Delta m_T}{b_0 - m_0 - \Delta m_S} - m_p(1+D)\frac{K}{b_0} < 0, \tag{S46}$$

in the case where the carrying capacity K is large or the cost of contesting a shelter (proportional to the product m_p) is high, one can easily see that $F'(0) < 0$. Thus adding shelters in the initially spatially homogeneous environment would result in a decrease of the overall population size.

8.2 Monomorphic population: simulated examples of different parameters

Here, we show that the key results of the main text are robust to a change in key parameters and that stability is maintained. We consider a monomorphic population with $B = 0.5$, despite a change in key parameters we still observe the dramatic drop in population densities as we increase the number of shelters N. Below, in Figs. S6 -S12 we show some examples of typical patterns of variation of the population density $F^*(N)$.

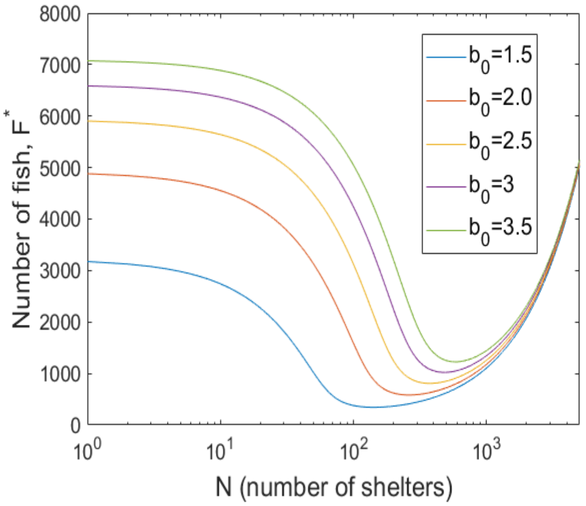

Figure S6: Dependence of the total population number F^* (measured in individuals) on the number of shelters for differing values of b_0 (the maximal per capita reproduction rate of individuals, measured in $1/year$). For the monomorphic population we fix $B = 0.5$ and unless stated otherwise all parameters are as given in Table 1 of the main text. The considered spatial area is $1ha$.

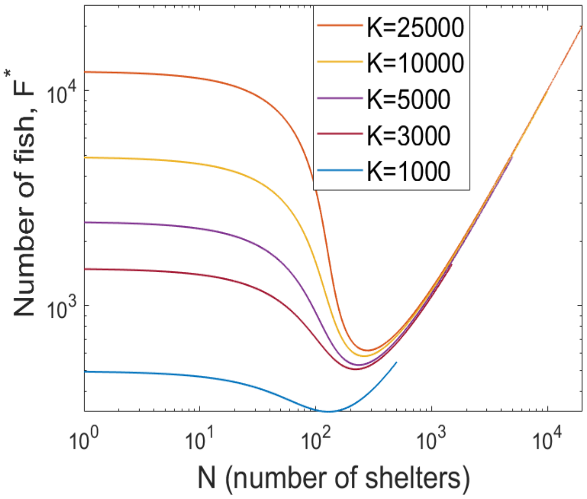

Figure S7: Dependence of the total population number F^* (measured in individuals) on the number of shelters for differing values of K (the carrying capacity, measured in fish numbers). For the monomorphic population we fix $B = 0.5$ and unless stated otherwise all parameters are as given in Table 1 of the main text. The considered spatial area is $1ha$.

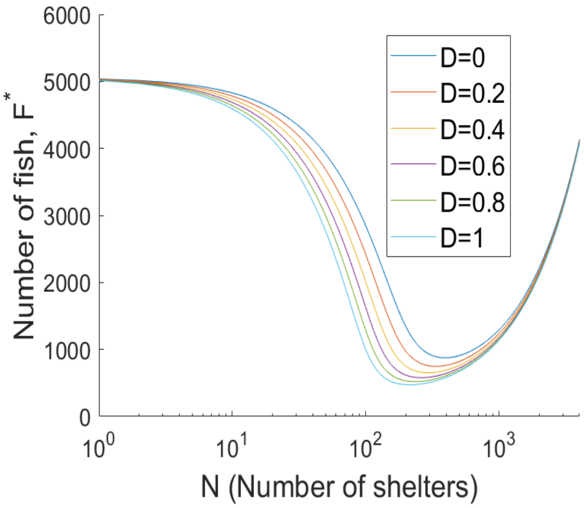

Figure S8: Dependence of the total population number F^* (measured in individuals) on the number of shelters for differing values of D (reduction in the cost of fighting when defending a shelter compared to invading, dimensionless parameter). For the monomorphic population we fix $B = 0.5$ and unless stated otherwise all parameters are as given in Table 1 of the main text. The considered spatial area is $1ha$.

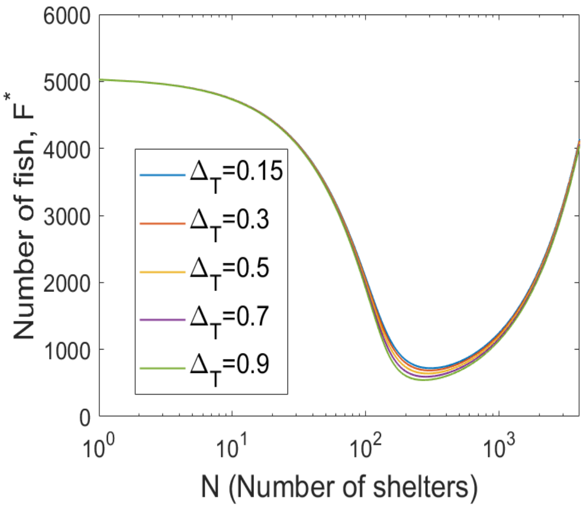

Figure S9: Dependence of the total population number F^* (measured in individuals) on the number of shelters for differing values of Δ_T (the parasite/predator mortality for sheltering individuals, measured in $1/year$). Here $\nu_\mu = 0.02 \quad 1/year$. For the monomorphic population we fix $B = 0.5$ and unless stated otherwise all parameters are as given in Table 1 of the main text. The considered spatial area is $1ha$.

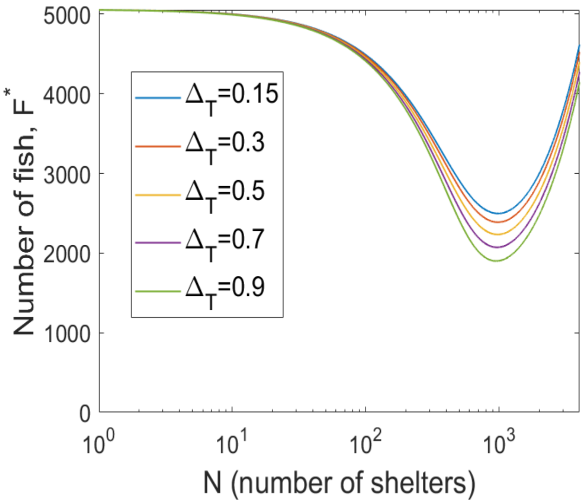

Figure S10: Dependence of the total population number F^* (measured in individuals) on the number of shelters for differing values of Δ_T (the parasite/predator mortality for sheltering individuals, measured in $1/year$). Here $\nu_\mu = 0.004 \quad 1/year$. For the monomorphic population we fix $B = 0.5$ and unless stated otherwise all parameters are as given in Table 1 of the main text. The considered spatial area is $1ha$.

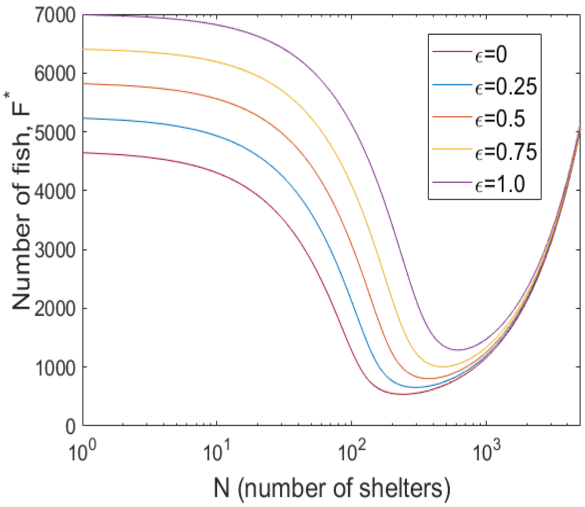

Figure S11: Dependence of the total population number F^* (measured in individuals) on the number of shelters for differing values of ϵ (the impact of the boldness of the severity of all parasite and predation induced mortalities, dimensionless parameter). For the monomorphic population we fix $B = 0.5$ and unless stated otherwise all parameters are as given in Table 1 of the main text. The considered spatial area is $1ha$.

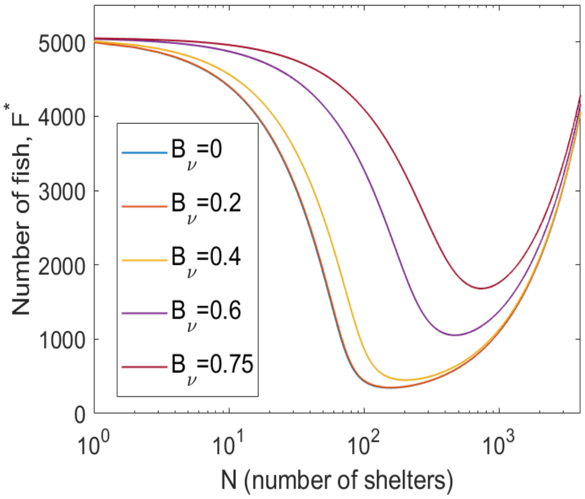

Figure S12: Dependence of the total population number F^* (measured in individuals) on the number of shelters for differing values of B_ν (this dimensionless parameter is included in the shelter search rate function $\nu(B)$). For the monomorphic population we fix $B = 0.5$ and unless stated otherwise all parameters are as given in Table 1 of the main text. The considered spatial area is $1ha$.

8.3 Polymorphic population: simulated examples for different parameters

We have fulfilled sensitivity analysis of the model for the case, where the population is polymorphic. The results are presented in Figs. S13-S21. As before, we are interested in plotting the dependence of the equilibrium population density F^* on the number of shelters N. We explored different scenarios of inheritance described the the coefficient D_w, in particular, clonal inheritance, mutations with uniform genetic mixing, and mixing with small mutations. In our study, we varied several model parameters, and, for most combinations of parameters, we found that the total population density changes only slightly with the number of shelters. For the sake of brevity, here we present the results for variation of parameters b_0, D, Δ_S, ν_μ and ϵ.

Our comparison of the population densities constructed for monomorphic and polymorphic populations shows that in the latter case the density F^* varies much less than in the case of a monomorphic population. Interestingly, this observation holds for the case of positive and negative trade-offs between the mortality and boldness described by parameters ϵ, in particular, when $\epsilon = 0$, i.e. in the absence of a trade-off between the boldness and the mortality.

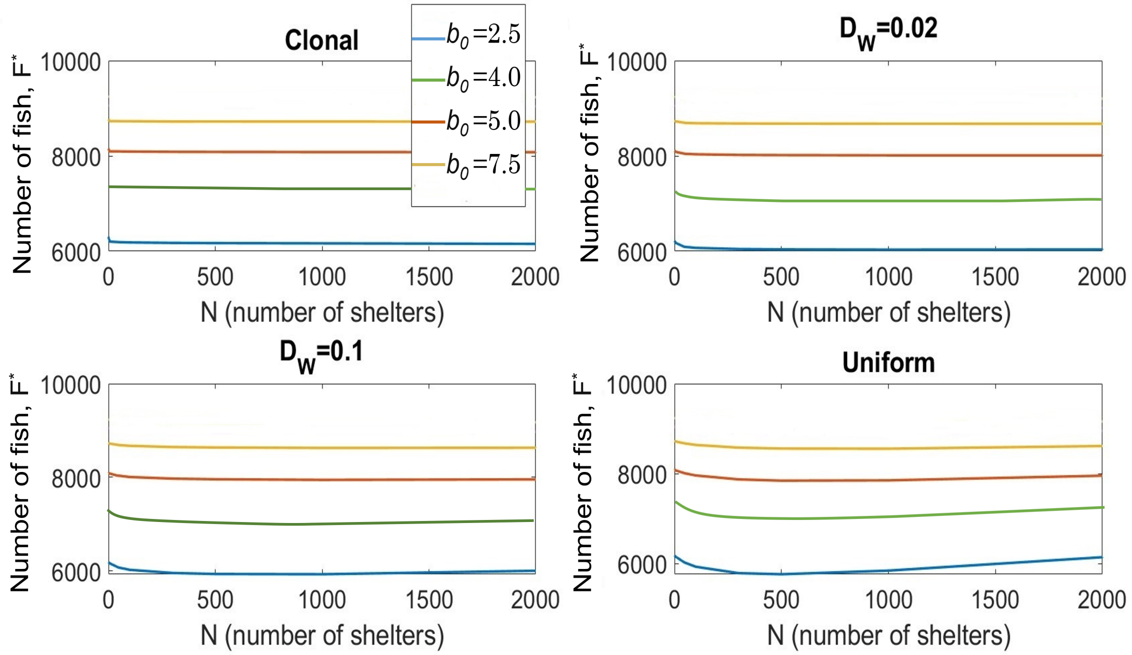

Figure S13: Dependence of the total population number F^* (measured in individuals) on the number of shelters for differing values of b_0 (the maximal per capita birth rate, measured in $1/year$) and D_w (the kernel determining the strength of heredity of boldness, dimensionless). All parameters are as given in Table 1 of the main text. The considered spatial area is $1ha$.

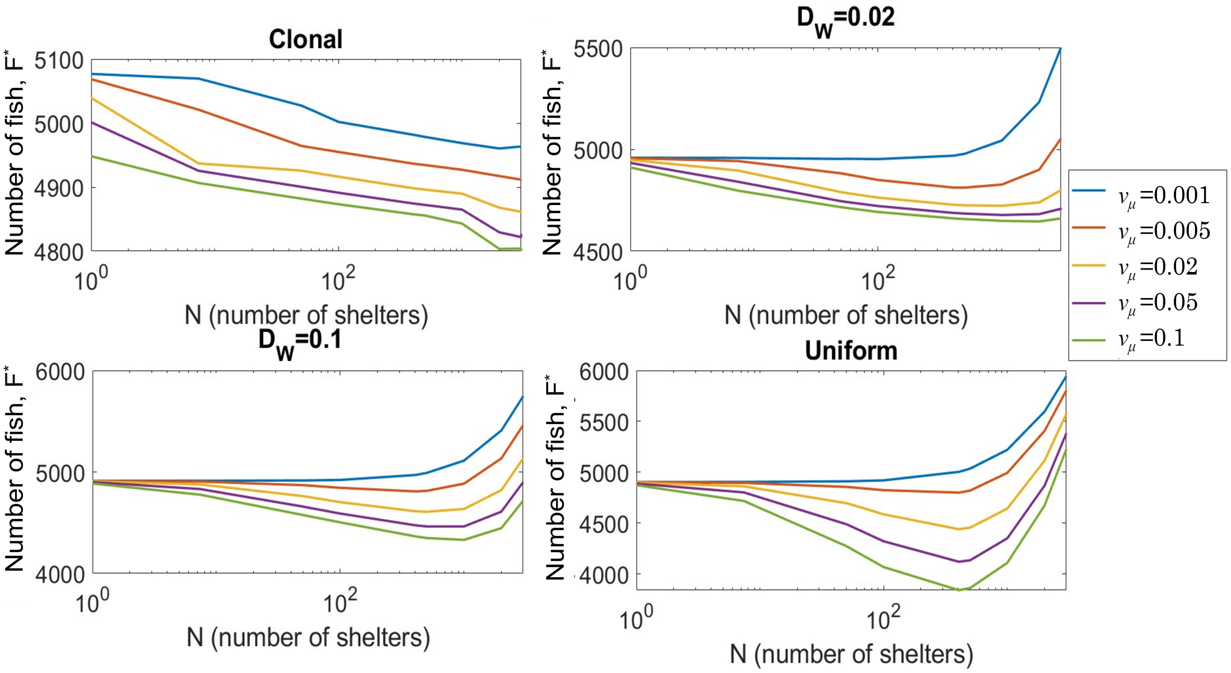

Figure S14: Dependence of the total population number F^* (measured in individuals) on the number of shelters for differing values of ν_μ (maximal mortality rate due to contests for shelters (per shelter), measured in $1/year$) and D_w (the kernel determining the strength of heredity of boldness, dimensionless). All parameters are as given in Table 1 of the main text. The considered spatial area is $1ha$.

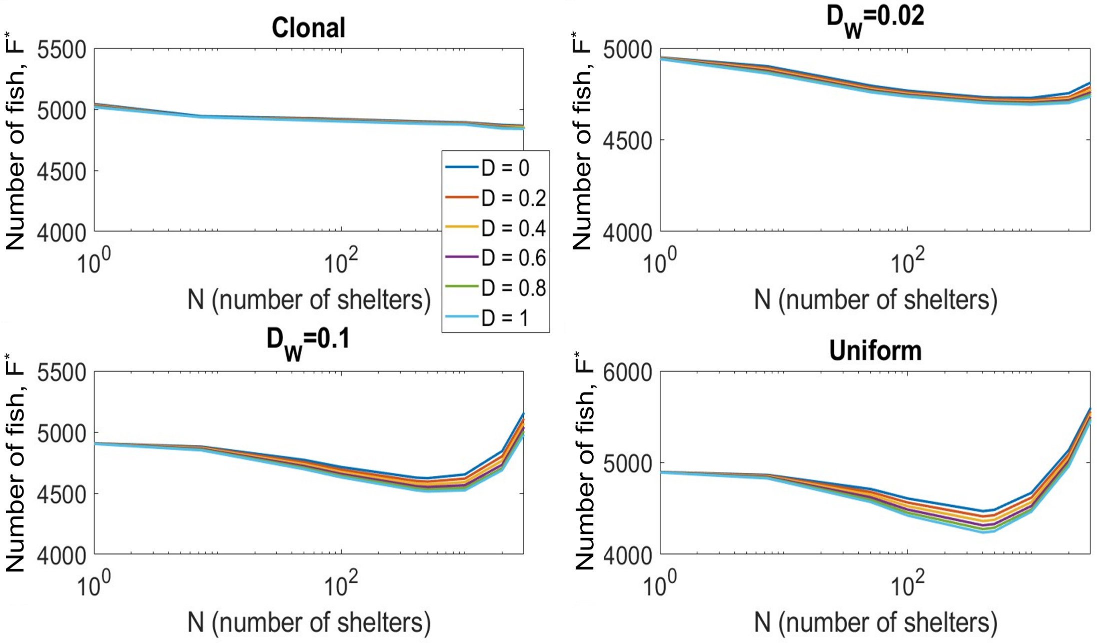

Figure S15: Dependence of the total population density F^* of fish (measured in individuals) on the number of shelters for differing values of D (reduction in the cost of fighting when defending a shelter compared to invading, dimensionless parameter) and D_w (the kernel determining the strength of heredity of boldness, dimensionless parameter). All parameters are as given in Table 1 of the main text. The considered spatial area is $1ha$.

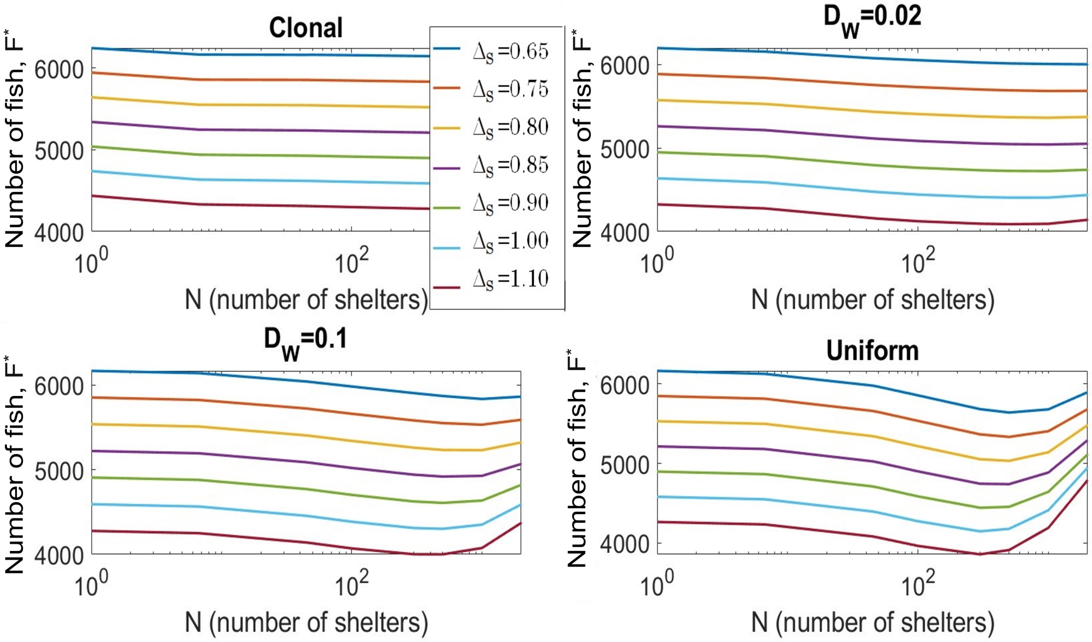

Figure S16: Dependence of total population density of the number of shelters for differing values of Δ_S (the parasite/predator mortality for shoaling individuals, measured in $1/year$) and D_w (the kernel determining the strength of heredity of boldness). All parameters are as given in Table 1 of the main text. The considered spatial area is $1ha$.

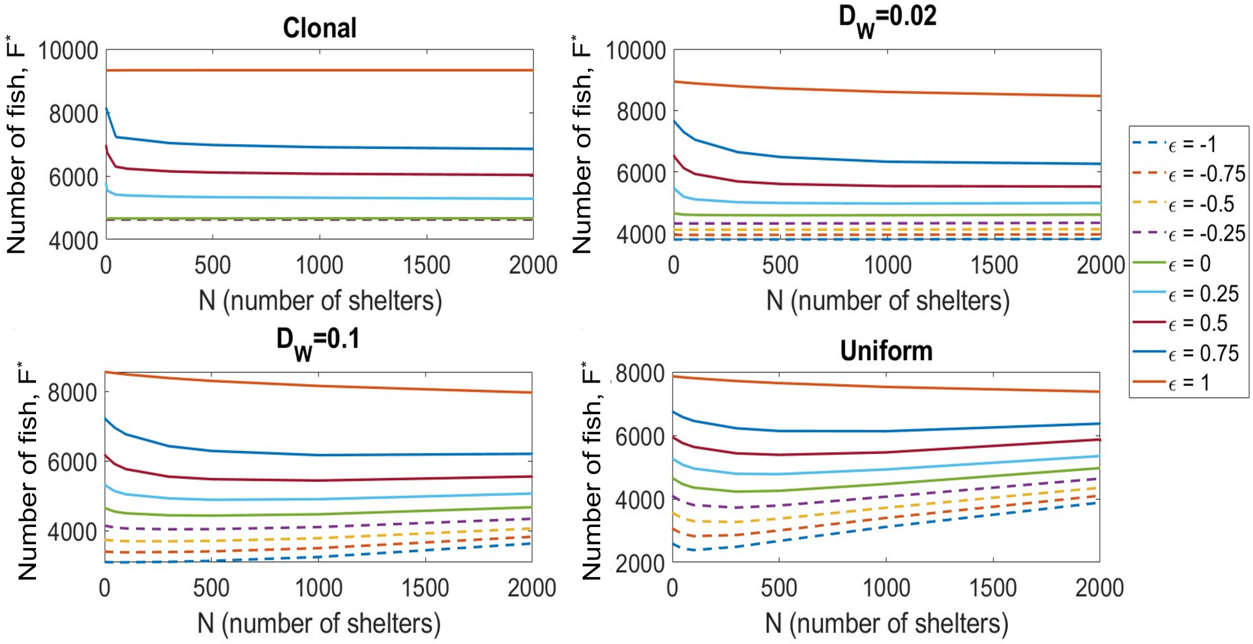

Figure S17: Dependence of the total population density F^* of fish (measured in individuals) on the number of shelters for differing values of ϵ (impact of the boldness of the severity of all parasite and predation induced mortality rates.) and D_w (the kernel determining the strength of heredity of boldness, dimensionless parameter). Here we allow negative and a zero values of ϵ to cover other scenarios beyond the rainbow trout-parasite system. Other parameters are as given in Table 1 of the main text. The considered spatial area is $1ha$.

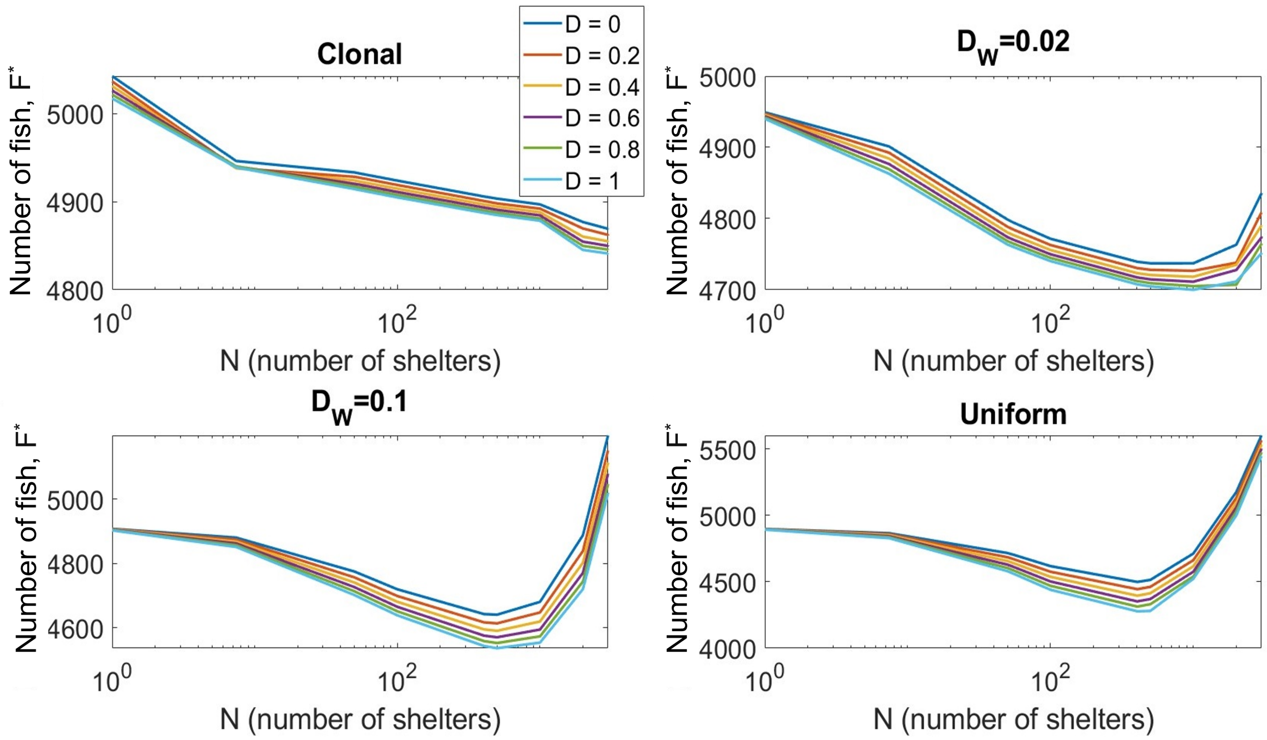

Figure S18: Dependence of the total population density of fish (measured in individuals) on the number of shelters for differing values of D (reduction in the cost of fighting when defending a shelter compared to invading, dimensionless parameter) and D_w (the kernel determining the strength of heredity of boldness, dimensionless parameter). Here the function $\nu(B)$ is parameterised by the linear function $\nu(B) = B$. Other parameters and functions are as given in Table 1 of the main text. The considered spatial area is $1ha$.

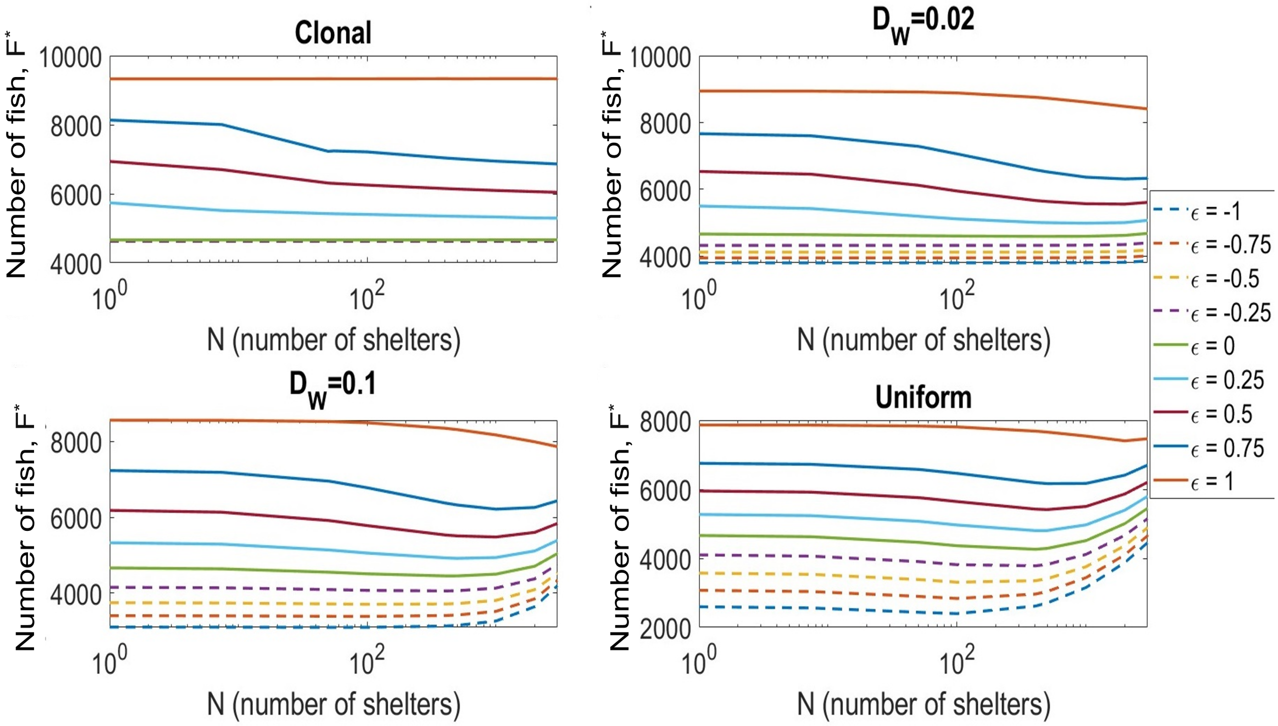

Figure S19: Dependence of the total population density F^* of fish (measured in individuals) on the number of shelters for differing values of ϵ (impact of the boldness of the severity of all parasite and predation induced mortality rates, dimensionless parameter) and D_w (the kernel determining the strength of heredity of boldness, dimensionless parameter). Here $\nu(B)$ is parameterised by the linear function $\nu(B) = B$. We allow negative and a zero values of ϵ to cover other scenarios beyond the rainbow trout-parasite system. Other parameters and functions are as given in Table 1 of the main text. The considered spatial area is $1ha$.

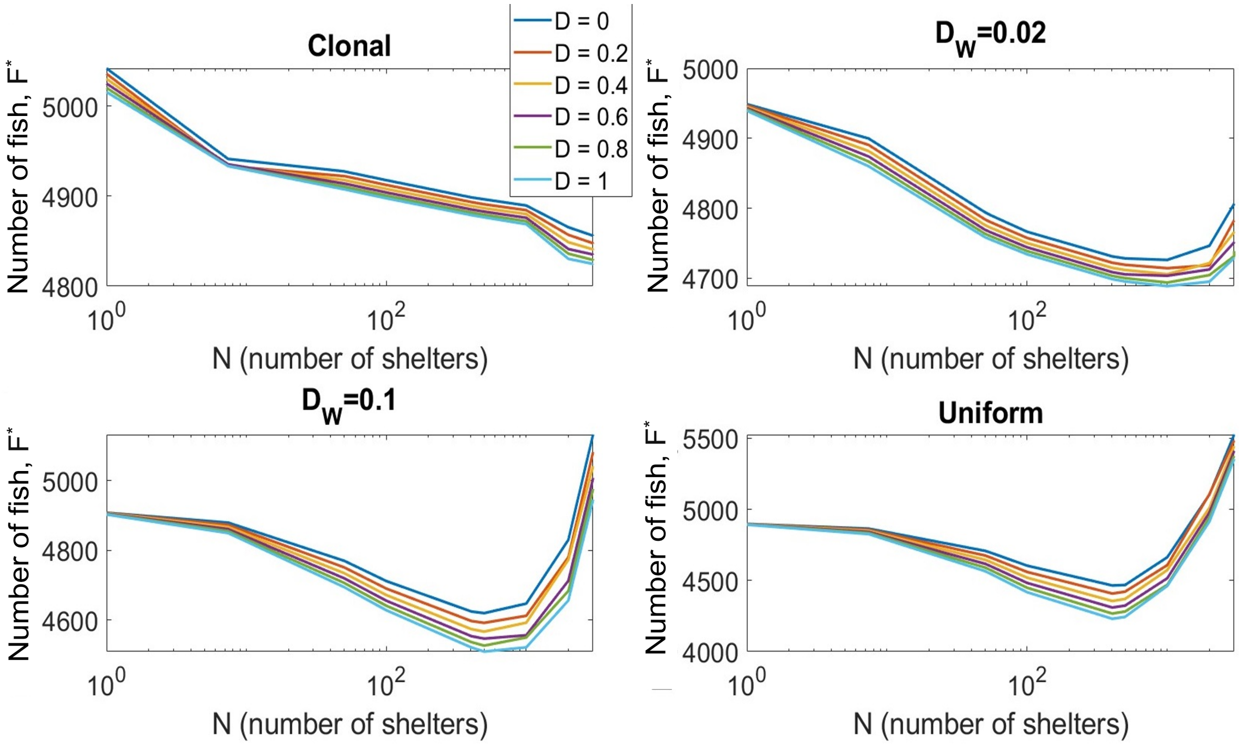

Figure S20: Dependence of the total population density of fish (measured in individuals) of the number of shelters for differing values of D (reduction in the cost of fighting when defending a shelter compared to invading, dimensionless parameter) and D_w (the kernel determining the strength of heredity of boldness, dimensionless parameter). Here $\nu(B)$ is parameterised by the function $\nu(B) = \frac{B(B_\nu+1)}{(B_\nu+B)}$. Other parameters and functions are as given in Table 1 of the main text. The considered spatial area is $1ha$.

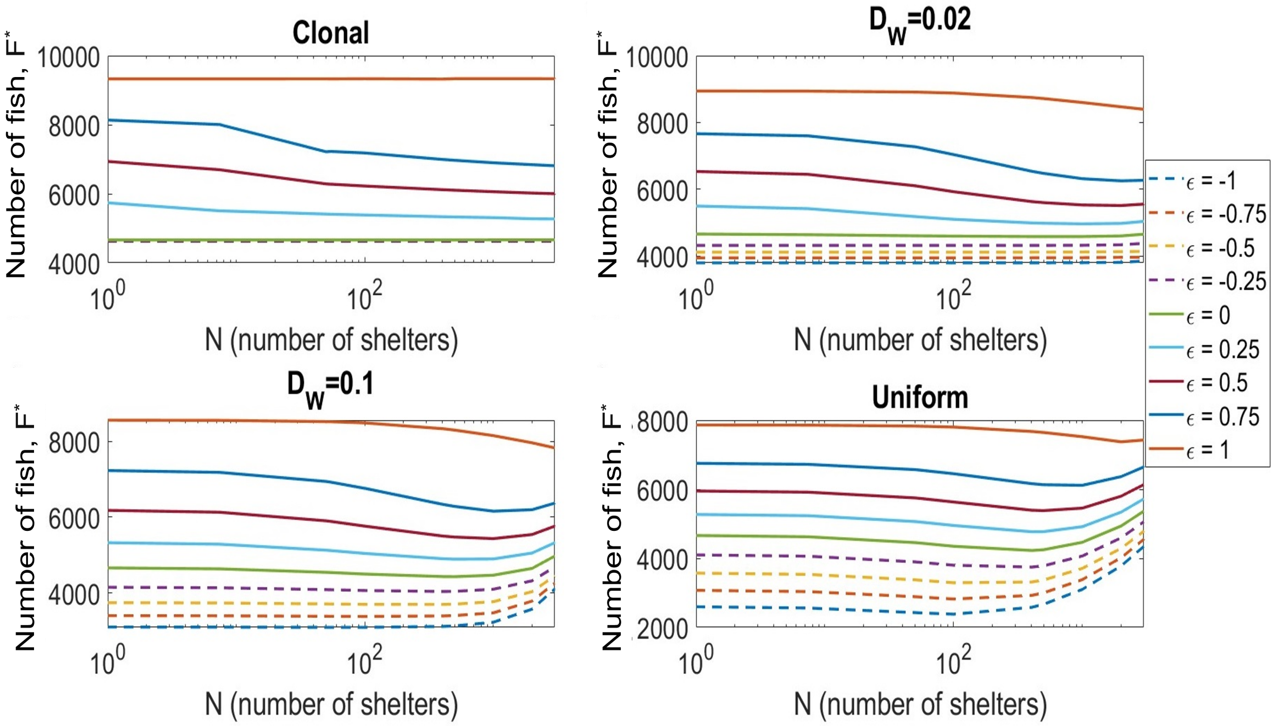

Figure S21: Dependence of the total population density F^* of fish (measured in individuals) on the number of shelters for differing values of ϵ (impact of the boldness of the severity of all parasite and predation induced mortality rates, dimensionless parameter) and D_w (the kernel determining the strength of heredity of boldness, dimensionless parameter). Here $\nu(B)$ is parameterised by the linear function $\nu(B) = \frac{B(B_\nu+1)}{(B_\nu+B)}$. We allow negative and a zero values of ϵ to cover other scenarios beyond the rainbow trout-parasite system. Other parameters and functions are as given in Table 1 of the main text. The considered spatial area is $1ha$.

8.4 Impact of the parameter ϵ on the evolutionary dynamics

We also investigated how the sign and the amplitude of trade-off between the boldness and the mortality (described by the parameter ϵ) affect the evolutionary dynamics in the system. The results of our direct simulations of evolution ($\epsilon \neq 0$) are presented in Fig. S22, whereas Fig. S23 shows possible configurations of the Pairwise Invasibility Plots (PIPs) constructed for $\epsilon = -1, -0.5, -0.1, 0.1, 0.5, 1$. Here we allow negative and a zero values of ϵ to cover other scenarios beyond the rainbow trout-parasite system. As previously, we applied the Adaptive Dynamics framework to model evolutionary behaviour. One can see from these figures that evolutionary branching is observed for both positive and negative values of parameter $\epsilon \neq 0$. The main difference, however, between the scenarios with $\epsilon < 0$ is that after branching the system evolves to the state, where the only remaining boldness types are the extreme ones ($B = 0$, $B = 1$). Another interesting observation for $\epsilon = 0.5$ (as well as some other values of ϵ, which are not shown here) is that instead of two final strains of boldness, there are three strains ($B_{1,2,3}$) which can survive.

We also explored the particular case, where there is no trade-off between the boldness and the mortality, i.e. $\epsilon = 0$. Fig. S24 shows the evolutionary dynamics and the corresponding PIP for $\epsilon = 0$. One can see from this figure that setting $\epsilon = 0$ preserves the branching behavior showing a qualitatively similar pattern as the one presented in the main text. This demonstrates the generic nature of branching behaviour within the polymorphic population.

Fig. S25 shows the role of parameters B_ν and μ (in the function $\nu(B) = B^\mu/(B^\mu + B_\nu^\mu)$) in the shape of PIPs.

Finally, we explored the PIPs for other two functional forms of $\nu(B)$, the results are presented in Figs. S26, S27, S28

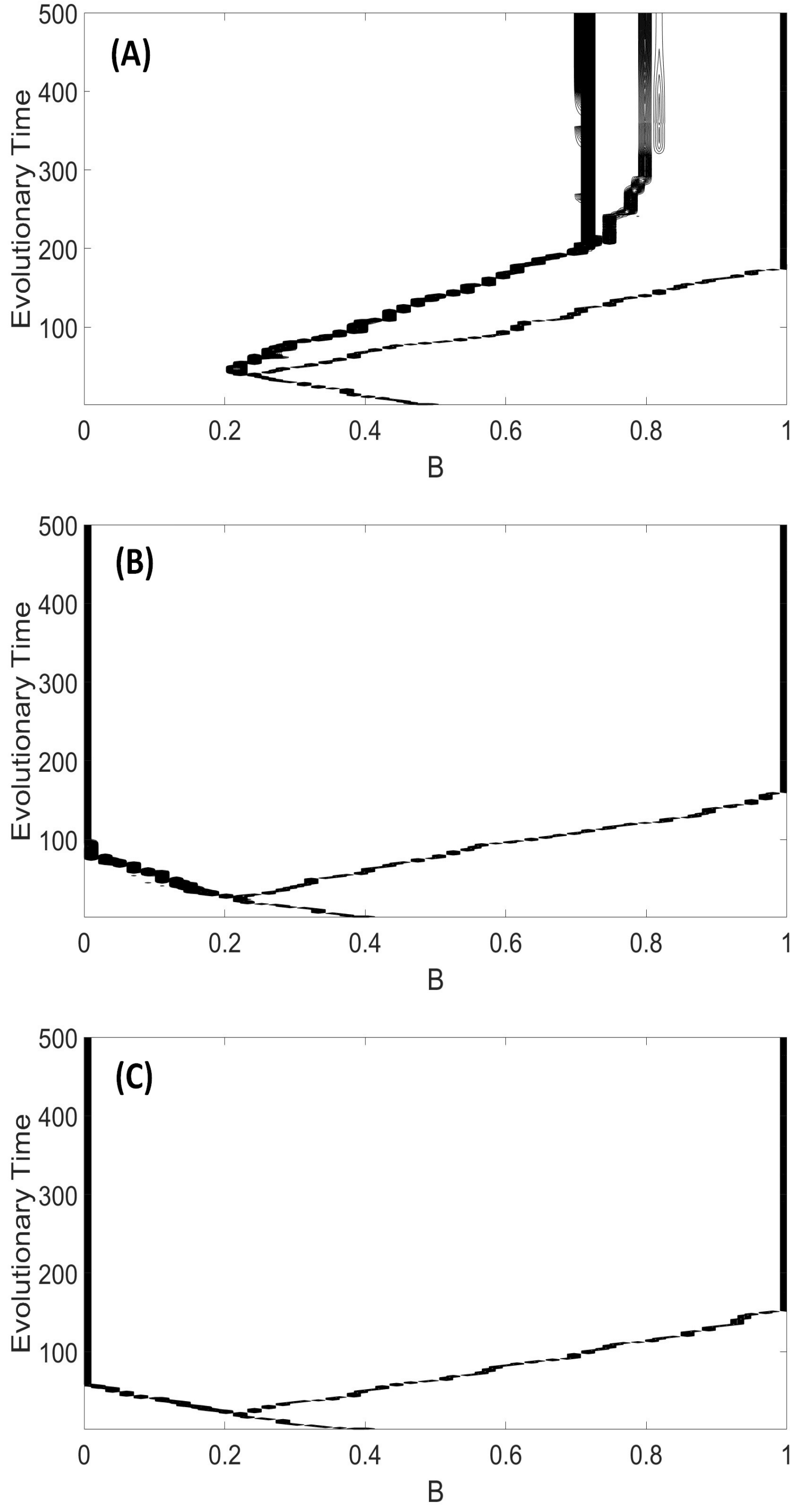

Figure S22: Direct numerical simulations demonstrate evolutionary branching in the system obtained for various values of $\epsilon \neq 0$. Panels (A),(B),(C) correspond to $\epsilon = 0.5, -0.1, -0.5$, respectively. The population sizes of various strains were modelled using equation (4) along with the solutions to the system given by (3), from the main text. We start with a single strain of boldness with $B = 0.4$. Here $N = 200$, and all other parameters are as given in Table 1 in the main text.

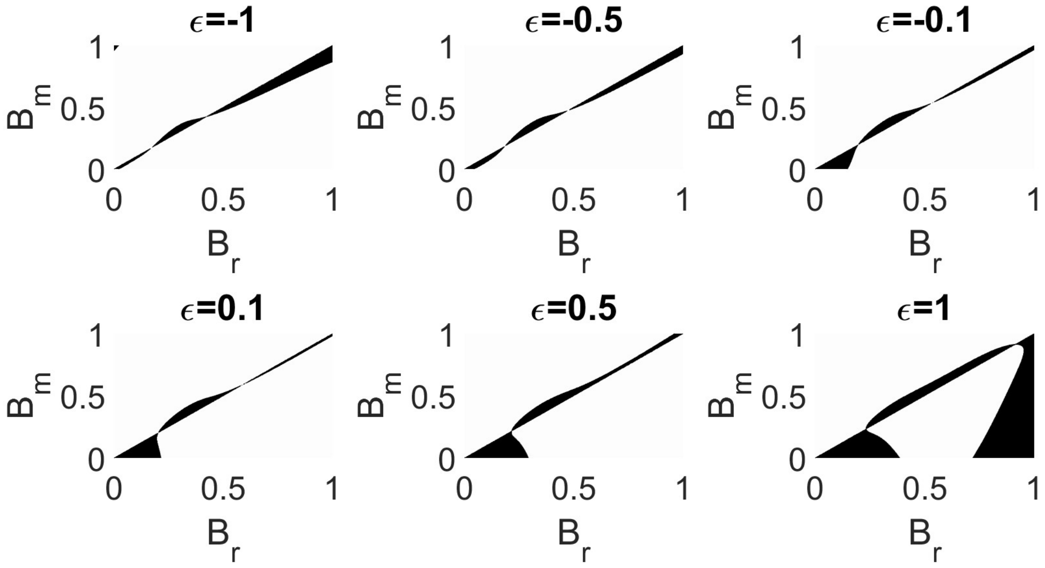

Figure S23: Pairwise Invasibility Plot (PIP) describing the invasion fitness of a rare mutant (B_m) into the population of a resident strain (B_r). The shown PIPs are constructed for various values of ϵ. The white regions represent a positive invasion fitness ($\lambda > 0$) and therefore a successful invasion, whereas the black regions represent a negative invasion fitness ($\lambda < 0$) and an unsuccessful invasion. We allow negative values of ϵ to cover other scenarios beyond the rainbow trout-parasite system. Here $N = 200$, and all other parameters are as given in Table 1.

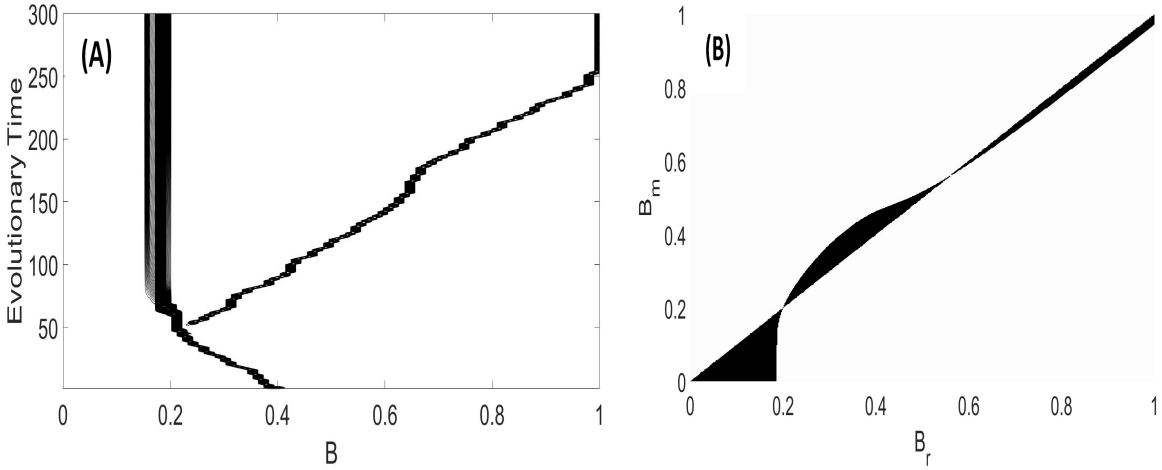

Figure S24: Evolutionary branching observed for $\epsilon = 0$. (A) Direct numerical simulations demonstrating evolutionary branching. The population sizes of various strains were modelled using equation (4) along with the solutions to the system given by (3), from the main text. We start with a single strain of boldness with $B = 0.4$. (B) The Pairwise Invasibility Plot (PIP) describing the invasion fitness of a rare mutant (B_m) into the population of a resident strain (B_r). The white and the black regions represent, respectively, a positive invasion fitness ($\lambda > 0$) and a negative invasion fitness ($\lambda < 0$). Here $N = 200$, and all other parameters are as given in Table 1 in the main text.

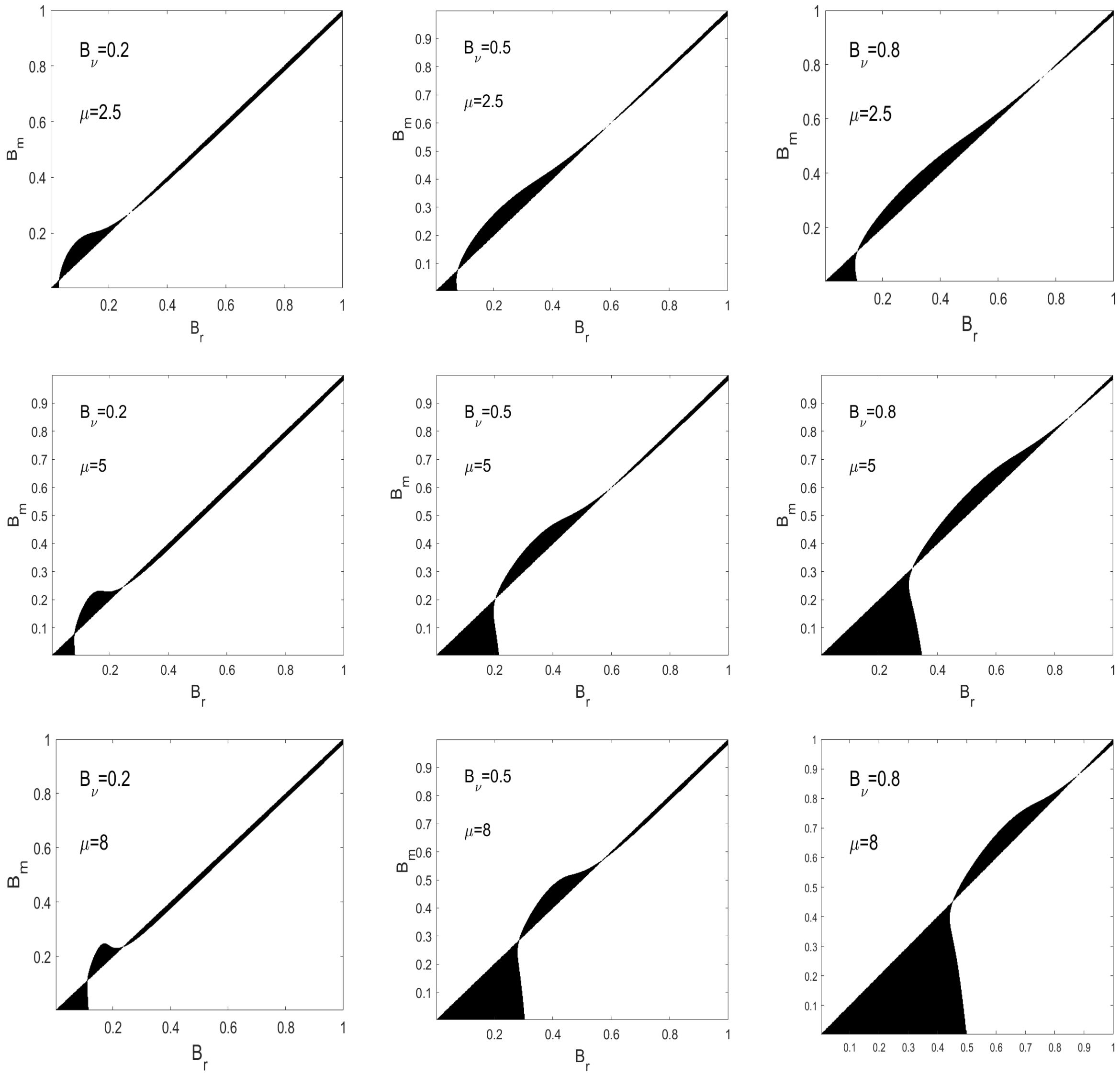

Figure S25: Pairwise Invasibility Plot (PIP) describing the invasion fitness of a rare mutant (B_m) into the population of a resident strain (B_r). The shown PIPs are constructed for various values of B_ν and μ in the function $\nu(B)$. The white regions represent a positive invasion fitness ($\lambda > 0$) and therefore a successful invasion, whereas the black regions represent a negative invasion fitness ($\lambda < 0$) and an unsuccessful invasion. Here $N = 200$, $\epsilon = 0.1$, and all other parameters are as given in Table 1. One can see that variation of model parameters does not affect the qualitative structure of the diagram, in particular, the branching point remains in the system.

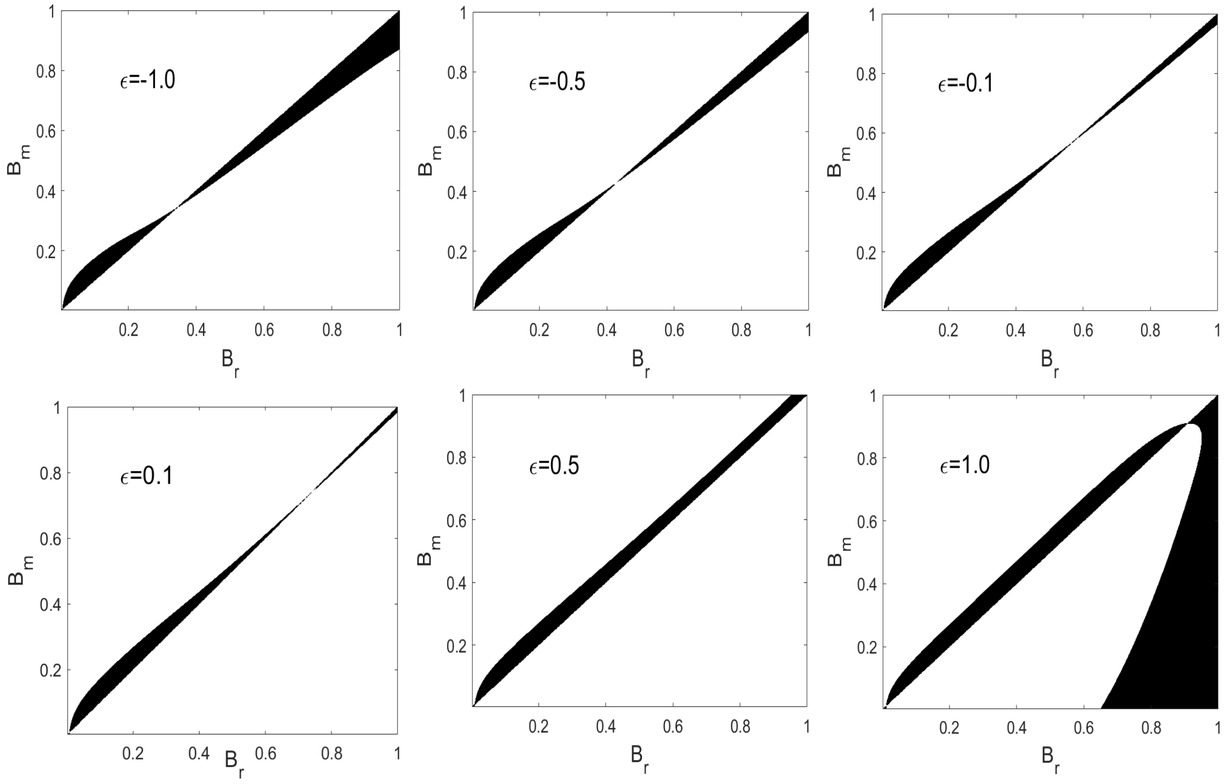

Figure S26: Pairwise Invasibility Plot (PIP) describing the invasion fitness of a rare mutant (B_m) into the population of a resident strain (B_r). The function $\nu(B)$ is parameterised by the linear function $\nu(B) = B$. We allow negative values of ϵ to cover other scenarios beyond the rainbow trout-parasite system. Here $N = 200$, and all other parameters and the functions are as given in Table 1. An evolutionary singular point corresponding to branching is located near zero.

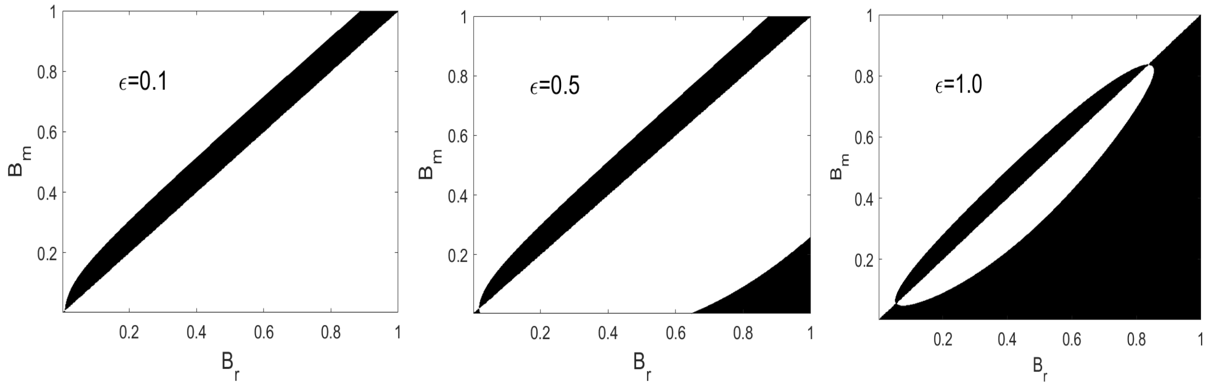

Figure S27: Pairwise Invasibility Plot (PIP) describing the invasion fitness of a rare mutant (B_m) into the population of a resident strain (B_r). The function $\nu(B)$ is parameterised by the linear function $\nu(B) = B$. We allow negative values of ϵ to cover other scenarios beyond the rainbow trout-parasite system. Here $N = 50$, and all other parameters and the functions are as given in Table 1.

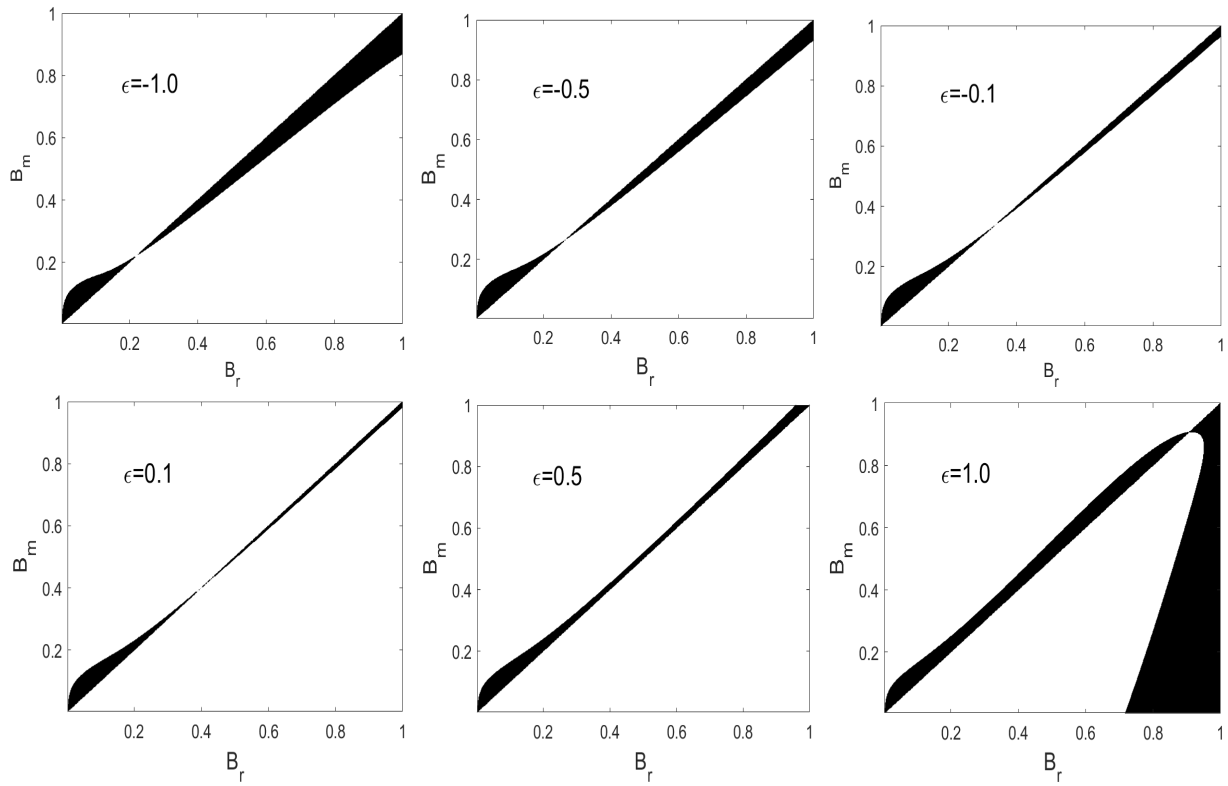

Figure S28: Pairwise Invasibility Plot (PIP) describing the invasion fitness of a rare mutant (B_m) into the population of a resident strain (B_r). The function $\nu(B)$ is parameterised by the linear function $\nu(B) = \frac{B(B_\nu+1)}{(B_\nu+B)}$. We allow negative values of ϵ to cover other scenarios beyond the rainbow trout-parasite system. Here $N = 200$, and all other parameters and the functions are as given in Table 1.

References

Abrams, P. A., Matsuda, H., & Harada, Y. (1993). Evolutionarily unstable fitness maxima and stable fitness minima of continuous traits. *Evolutionary Ecology*, *7*(5), 465–487.

Brännström, Å., Johansson, J., & Von Festenberg, N. (2013). The hitchhiker's guide to adaptive dynamics. *Games*, *4*(3), 304–328.

Christiansen, F. B. (1991). On conditions for evolutionary stability for a continuously varying character. *The American Naturalist*, *138*(1), 37–50.

Dieckmann, U., & Doebeli, M. (1999). On the origin of species by sympatric speciation. *Nature*, *400*(6742), 354–357.

Doebeli, M. (2011). *Adaptive diversification (mpb-48)* (Vol. 48). Princeton University Press.

Eshel, I. (1983). Evolutionary and continuous stability. *Journal of theoretical Biology*, *103*(1), 99–111.

Geritz, S. A., Metz, J. A., Kisdi, É., & Meszéna, G. (1997). Dynamics of adaptation and evolutionary branching. *Physical Review Letters*, *78*(10), 2024.

Kisdi, É., & Meszéna, G. (1993). Density dependent life history evolution in fluctuating environments. In *Adaptation in stochastic environments* (pp. 26–62). Springer.

Taylor, P. D. (1989). Evolutionary stability in one-parameter models under weak selection. *Theoretical population biology*, *36*(2), 125–143.
