# Supplementary material for: Revisiting the role of behavior-mediated structuring in the survival of populations in hostile environments
Source: Commun Biol. 2024 Jan 12;7:93. doi: 10.1038/s42003-023-05731-z (PMC10786947; doi:10.1038/s42003-023-05731-z)
Supplement: Supplementary file 2 — Reporting Summary [file 42003_2023_5731_MOESM2_ESM.pdf]

nature portfolio

Corresponding author(s): Andrew Morozov

Last updated by author(s): Dec 14, 2023

Reporting Summary

Nature Portfolio wishes to improve the reproducibility of the work that we publish. This form provides structure for consistency and transparency in reporting. For further information on Nature Portfolio policies, see our Editorial Policies and the Editorial Policy Checklist.

Statistics

For all statistical analyses, confirm that the following items are present in the figure legend, table legend, main text, or Methods section.

n/a	Confirmed	
☐	☒	The exact sample size (*n*) for each experimental group/condition, given as a discrete number and unit of measurement
☐	☒	A statement on whether measurements were taken from distinct samples or whether the same sample was measured repeatedly
☐	☒	The statistical test(s) used AND whether they are one- or two-sided<br>*Only common tests should be described solely by name; describe more complex techniques in the Methods section.*
☒	☐	A description of all covariates tested
☐	☒	A description of any assumptions or corrections, such as tests of normality and adjustment for multiple comparisons
☐	☒	A full description of the statistical parameters including central tendency (e.g. means) or other basic estimates (e.g. regression coefficient) AND variation (e.g. standard deviation) or associated estimates of uncertainty (e.g. confidence intervals)
☐	☒	For null hypothesis testing, the test statistic (e.g. *F*, *t*, *r*) with confidence intervals, effect sizes, degrees of freedom and *P* value noted<br>*Give P values as exact values whenever suitable.*
☒	☐	For Bayesian analysis, information on the choice of priors and Markov chain Monte Carlo settings
☒	☐	For hierarchical and complex designs, identification of the appropriate level for tests and full reporting of outcomes
☒	☐	Estimates of effect sizes (e.g. Cohen's *d*, Pearson's *r*), indicating how they were calculated

Our web collection on statistics for biologists contains articles on many of the points above.

Software and code

Policy information about availability of computer code

Data collection	No software was used
Data analysis	Excel 2016 (Microsoft office), Statistica

For manuscripts utilizing custom algorithms or software that are central to the research but not yet described in published literature, software must be made available to editors and reviewers. We strongly encourage code deposition in a community repository (e.g. GitHub). See the Nature Portfolio guidelines for submitting code & software for further information.

Data

Policy information about availability of data

All manuscripts must include a data availability statement. This statement should provide the following information, where applicable:

- Accession codes, unique identifiers, or web links for publicly available datasets
- A description of any restrictions on data availability
- For clinical datasets or third party data, please ensure that the statement adheres to our policy

Data are included in the supplementary material

Research involving human participants, their data, or biological material

Policy information about studies with human participants or human data. See also policy information about sex, gender (identity/presentation), and sexual orientation and race, ethnicity and racism.

Reporting on sex and gender	n/a
Reporting on race, ethnicity, or other socially relevant groupings	n/a
Population characteristics	n/a
Recruitment	n/a
Ethics oversight	n/a

Note that full information on the approval of the study protocol must also be provided in the manuscript.

Field-specific reporting

Please select the one below that is the best fit for your research. If you are not sure, read the appropriate sections before making your selection.

☒ Life sciences ☐ Behavioural & social sciences ☐ Ecological, evolutionary & environmental sciences

For a reference copy of the document with all sections, see nature.com/documents/nr-reporting-summary-flat.pdf

Life sciences study design

All studies must disclose on these points even when the disclosure is negative.

Sample size	We tested 7 independent datasets grouped in 3 experiments:<br>A. Fish reaction time dataset: 120 fish (55 fast, 57 slow, 8 excluded)<br>B. Infection rates in fish dataset: 40 fast, 40 slow fish (exposed individually and in groups of 4)<br>C. Infection vs Anticipated threats datasets:<br>- With shelters – 20 fish;<br>- Without shelters, dark bottom – 20 fish;<br>- Without shelters, light bottom – 20 fish;<br>- Fish in groups – 100 fish<br><br>The number of datasets was chosen to include the behavioral traits (fast and slow fish) and environmental conditions (shelters, substrate coloration, conspecifics) as factors influencing infection.
Data exclusions	Fish reaction time dataset: We excluded 8 fish because they did not emerge from a start chamber within a standard time.
Replication	All effects were replicated in B and C independent datasets.
Randomization	Fish for testing their reaction time were taken randomly from a holding tank. For dataset B, fish were taken randomly from either a "fast", or a "slow" pool. For dataset C, fish were taken randomly from a holding tank.
Blinding	For datasets B and C, quantification of parasites in the fish eyes was performed without experimenter knowing what behavioral category fish belonged to and which experimental environment (in terms of anticipated threat) they were exposed to.

Reporting for specific materials, systems and methods

We require information from authors about some types of materials, experimental systems and methods used in many studies. Here, indicate whether each material, system or method listed is relevant to your study. If you are not sure if a list item applies to your research, read the appropriate section before selecting a response.

Materials & experimental systems

n/a	Involved in the study
☒	☐ Antibodies
☒	☐ Eukaryotic cell lines
☒	☐ Palaeontology and archaeology
☐	☒ Animals and other organisms
☒	☐ Clinical data
☒	☐ Dual use research of concern
☒	☐ Plants

Methods

n/a	Involved in the study
☒	☐ ChIP-seq
☒	☐ Flow cytometry
☒	☐ MRI-based neuroimaging

Animals and other research organisms

Policy information about studies involving animals; ARRIVE guidelines recommended for reporting animal research, and Sex and Gender in Research

Laboratory animals	The study did not involve laboratory animals
Wild animals	We used 0+ Oncorhynchus mykiss. The fish specimens of rainbow trout used in our experiments were obtained from a commercial fish farm in Finland. Prior to the experiments, approximately 400 fish were kept in a flow-through tank of 2.5 m3 on 15:9 L : D cycle at 15-16 degrees C and fed with commercial pelleted food (1.5 mm size, Nutra Parr LB, Norway). The food was the same as at the fish farm.
Reporting on sex	Sex-based analysis has not performed since previous literature has not reported dependence the parasite acquisition for young salmonids
Field-collected samples	The study has not involved collecting fish from fields.
Ethics oversight	The level of experimental D. pseudospathaceum infection was maintained at a much lower level than the maximum values reported for naturally occurring infections (up to 200-500 individuals per fish). The mortality of infected fish in the experiments was less than 1% and did not exceed that of the control sh. No visible damage was observed in any fish. We minimized the required number of animals that were killed and dissected. Experimental fish were killed at the end of the tests with an overdose of MS222 and dissected. In total, 240 experimentally infected fish were killed. The experiments were conducted with the permission of the National Animal Experiment Board, Centre for Economic Development, Transport and Environment of South Finland (license number ESAVI/6759/04.10.03/2011).

Note that full information on the approval of the study protocol must also be provided in the manuscript.
